# CellTree: an R/bioconductor package to infer the hierarchical structure of cell populations from single-cell RNA-seq data

**DOI:** 10.1186/s12859-016-1175-6

**Published:** 2016-09-13

**Authors:** David A. duVerle, Sohiya Yotsukura, Seitaro Nomura, Hiroyuki Aburatani, Koji Tsuda

**Affiliations:** 1 Graduate School of Frontier Sciences at the University of Tokyo, 5-1-5 Kashiwa-no-ha, Kashiwa, Japan; 2 Bioinformatics Center, Institute for Chemical Research, Kyoto University, Gokasho, Uji, Japan; 3 Genome Science Division, Laboratory of Systems Biology and Medicine, Research Center for Advanced Science and Technology, The University of Tokyo, 4-6-1 Komaba, Tokyo, Japan; 4 Center for Materials Research by Information Integration, National Institute for Materials Science, 1-2-1 Sengen, Tsukuba, Japan; 5 Biotechnology Research Institute for Drug Discovery, National Institute of Advanced Industrial Science and Technology, 2-4-7 Aomi, Koto-ku, Tokyo, Japan

**Keywords:** Single-cell RNA-seq, Cell differentiation, Cell heterogeneity, Human stem cell

## Abstract

**Background:**

Single-cell RNA sequencing is fast becoming one the standard method for gene expression measurement, providing unique insights into cellular processes. A number of methods, based on general dimensionality reduction techniques, have been suggested to help infer and visualise the underlying structure of cell populations from single-cell expression levels, yet their models generally lack proper biological grounding and struggle at identifying complex differentiation paths.

**Results:**

Here we introduce cellTree: an R/Bioconductor package that uses a novel statistical approach, based on document analysis techniques, to produce tree structures outlining the hierarchical relationship between single-cell samples, while identifying latent groups of genes that can provide biological insights.

**Conclusions:**

With cellTree, we provide experimentalists with an easy-to-use tool, based on statistically and biologically-sound algorithms, to efficiently explore and visualise single-cell RNA data. The cellTree package is publicly available in the online Bionconductor repository at: http://bioconductor.org/packages/cellTree/.

**Electronic supplementary material:**

The online version of this article (doi:10.1186/s12859-016-1175-6) contains supplementary material, which is available to authorized users.

## Background

Single-cell RNA sequencing, one of the most significant advances in recent genomics [1], is fast becoming the norm in whole-transcriptome expression profiling, providing unique insights into the exact state of individual cells throughout biological processes, such as cell differentiation or tumorigenesis. In opposition to traditional batch sequencing, single-cell expression measurements are not affected by cell heterogeneity within the sample and give an exact snapshot of gene activity at a specific time. The very low noise level and virtual absence of sample variance opens the door to more exact statistical modelling of gene regulatory activity and might be the key to successful regulatory network inference [2, 3].

To fulfil these promises, many challenges specific to single-cell expression data analysis must first be solved [4], such as the difficulty to infer the true hierarchy (or chronological order) of individual cells sampled in the same conditions (temporal or spatial). For instance, due to the destructive nature of RNA-seq measurements, time-series analysis is approximated by repeated sampling at intervals, introducing new confounding factors tied to the biological specificities of each cell sampled, as well as the risk of “pollution” by unrelated cell lines and difficulties in identifying multiple sub-differentiation branches. Analysing the similarities between cells’ expression profiles seems the key to inferring the true structure of the cell population but is made especially complex by the very high dimensionality of gene expression measurements.

When cell populations can be assumed to belong to a temporal continuum, the standard approach is to assign each cell a biological “pseudotime” along which they can be ordered. In the absence of known subsets of marker genes [5], the vast majority of existing methods for pseudotime estimation crucially rely on classical dimension-reduction techniques to produce an embedding where pairwise cell distances can be more easily computed [6]: Independent Component Analysis (ICA) [7], Principal Component Analysis (PCA) [8, 9] or Multidimensional Scaling (MDS) [10].

Such dimension-reduction methods can be applied with no knowledge of the underlying structure of the data. But this versatility comes at the cost of clarity in the embedding: it is difficult, often impossible, to find a plausible biological representation of the lower-dimensional components obtained [11]. Furthermore, the aggressive pre-treatment thresholding commonly required (reducing the initial input to a few hundred high-variance genes) runs the risk of over-simplifying the model by discarding low-variance genes that may play a role in some aspects of the process studied. In the case of methods like ICA (used by Monocle), the assumption of statistical independence between components is highly questionable: due to the heavy overlap between regulatory pathways, gene expression levels would presumably show correlation between different stages of a cell process. While these approaches can typically give good results on straightforward cell differentiation along a single path (where a main component representing time is sufficient to separate and order the cells), they show their limits when multiple lineages are mixed together. Not only can they fail to recognise the heterogeneity of differentiating subtypes, but they also cannot easily assign any biological interpretation to the model used.

By contrast, our proposed method uses a Bayesian model better adapted to known models of gene regulation, and can produce an embedding that uses a larger number of input features without such stringent thresholding step:

When considering a number of single-cell expression measurements taken over time (e.g during cell differentiation) or space (e.g. across tissues), we expect specific metabolic pathways, and the genes that compose them, to be activated according to the current biological state of the cell sampled. We can therefore hypothesise the existence of groups of genes, and their respective regulatory subnetworks, that broadly characterise each of the steps in the cellular process studied. Because of the nature of regulatory networks, such groups could potentially involve hundreds or even thousands of genes (albeit at differing levels of importance), with a lot of potential overlap between groups.

To identify and utilise this group structure, our suggested method adapts a new statistical approach, borrowed from natural language processing, known as Latent Dirichlet Allocation (LDA; [12]). LDA assumes the existence of a number of underlying “topics” that contribute, as a mixture, to explain each cell’s transcriptional activity. By comparing the different per-cell topic histograms, we can evaluate their similarity and infer complex hierarchical structures. By looking at the topics themselves, we can obtain useful biological insights on the gene sets characterising the different stages of that hierarchy (see Implementation).

Much like other methods, an important step in our approach is the construction of a visual representation of the cell population based on this lower-dimensional model. To better help this visualisation, we introduced “backbone trees”: a new type of tree structure specifically designed to easily visualise cells along complex differentiation paths (see Implementation). In contrast with existing methods, however, we are able to analyse the latent groups of genes, called “topics” in the LDA model, that are used to model the cell population. An overview of these topics can directly be obtained as a list of genes ranked by their probability in each per-topic distribution, making it easy to verify if certain genes are particularly attached to a stage. For a more in-depth analysis, we use gene ontology (GO) terms [13]: statistical testing allows us to select gene ontology terms that are enriched for a topic. Looking at these terms, as a list or as a subgraph of the overall GO graph, gives a quick overview of the cellular components, biological processes and molecular functions associated with each topic and provide a helpful narrative for the results obtained.

## Implementation

### Using latent Dirichlet allocation

Latent Dirichlet Allocation (LDA) is a Bayesian mixture model, initially developed for the analysis of text documents, that allows sets of observations to be explained by unobserved groups. In text analysis, the model assumes that each document is a mixture of topics (represented as a probability distribution with a Dirichlet prior) and each word is the result of one of the document’s topic.

In the context of single-cell data analysis, documents become cells and discretised gene expression levels replace word frequencies. The fitted LDA model for our data is therefore composed of a set of topic distributions for each cell, and per-topic gene distributions. Per-cell topic histograms can then be used as a low-dimensional embedding to evaluate cell similarity and infer hierarchical relationship, while analysis of the topics themselves can provide useful biological insights on the sets of genes driving the different stages of the process studied.

Given *M* cells, *V* expressed genes and a choice of *K* topics, the model is therefore made up of two sets of Dirichlet distributions:

$$\begin{array}{@{}rcl@{}} \boldsymbol{\phi}_{k} \sim \text{Dirichlet}_{V}(\boldsymbol{\beta}), k = 1 \dots K  \\ \boldsymbol{\theta}_{d} \sim \text{Dirichlet}_{K}(\boldsymbol{\alpha}), d = 1 \dots M  \end{array} $$

where ***α*** and ***β*** are vectors of length *K* and *V* representing the prior weights of per-cell topics and per-topic genes, respectively. The use of smaller values of ***α*** and ***β*** makes it possible to control the sparsity of the model (i.e. the number of topics per cell and number of genes per topic).

The parameters to the posterior distributions that make the LDA model are learnt from the data (a matrix of gene expression levels for each cell) using approximate inference techniques [14]. Initially solved with variational inference [12], this problem is now more efficiently tackled using Gibbs Sampling (including the LDA implementation used by cellTree): a type of Markov Chain Monte Carlo algorithm that converges iteratively toward a stationary distribution that satisfyingly approximates the target joint distribution. In the particular case of LDA, the implementation of Gibbs Sampling makes use of some of the features of the model to greatly reduce the size of the joint distribution that must be evaluated, in a method called *Collapsed* Gibbs Sampling.

For an in-depth explanation of the mathematics behind the general LDA model, we recommend consulting David Blei’s original paper [12] along with more recent work on LDA inference methods [15, 16].

Among the many advantages of LDA as a dimension reduction method, its ability to handle very large-dimensional data and control model sparsity (through the priors of the Dirichlet distributions) make it easy to handle unknown data with relatively little pre-treatment. Generally, it is sufficient to log-transform expression values and removes genes with low standard-deviation, without more advanced method of gene set selection (these pre-treatments are done automatically by the default cellTree pipeline).

### Choosing number of topics

The main parameter to the LDA fitting procedure is the desired number of topics: *K* (best values for other hyper-parameters, such as ***α*** and ***β*** are automatically picked by the different fitting methods). As often with such statistical methods, a large number of topics (and therefore a more complex statistical model) can lead to overfitting, and it is therefore preferable to use the smallest possible number that provides a good explanation of the data. It must be noted, however, that while very large number of topics (leading to a very dense statistical model) would likely adversely affect performances, the population structure inferred by cellTree is relatively resistant to small variations in the number of topics used.

Because of the loose significance of the concept of ‘topics’ in the context of gene expression in a cell, it is difficult to reliably pick an exact number, based on biological knowledge alone. The standard method is to use cross-validation and likelihood maximisation, however the computation time for such an approach can be prohibitive on large data sets. A more time-efficient approach was suggested by Matthew Taddy [16], that uses model selection through joint Maximum-a-Posteriori (MAP) estimation and iteratively fits models of increasing complexity (using the previous fit’s residuals as a basis for the next one) to exhaustively look at a large range of topic numbers in a relatively small amount of time.

It is nonetheless possible to evaluate the sparsity of a fitted model associated to a chosen number of topics, by examining the gene ontology terms enriched for each topic (see Implementation): a lot of redundancy between enriched sets is a good indicator that the model could be made sparser.

### Extracting hierarchical structures

Extracting a hierarchical structure of the cell population from the lower-dimensional model follows the same general idea as other methods that rely on PCA or ICA for dimensionality reduction: by first computing a matrix of pairwise distance. We use the chi-square distance [17] to compare the topic histograms assigned to two cells *x* and *y*:

$$ \chi(\mathbf{x},\mathbf{y}) = \sqrt{\sum\limits_{k = 1 \dots K}{\frac{(x_{k} - y_{k})^{2}}{x_{k}+y_{k}}}} $$

This distance matrix obtained can be used with methods such as hierarchical clustering, or with various tree-building algorithms, to identify the underlying tree structure of the cells.

In the general case, the cell population is measured in batches of samples obtained in similar conditions (e.g. at specific time-points) that spread along a continuum between the different stages. One natural way to visualise such a structure is using a minimum spanning tree (MST).

Although many efficient algorithms exist to produce a minimum spanning tree from a distance metric, rooting such a tree is non-trivial and different choices for the root node can lead to very different structures. In some cases, sample labels can be used to identify the group of cells where the root should logically be (e.g. time 0 in a timeseries experiment). In that case, cellTree can use this information to pick the most central cell in the initial group using one of two main approaches:

- By first identifying the longest shortest path in the MST (a path whose length is the diameter of the tree) and picking the correct end, based on knowledge of the starting group. This approach would be particularly indicated if the dataset is known to represent a linear continuum of cells (with no branching).
- In cases where branching in the cell population is expected, it may be preferable to pick one of the starting group cells, based on the structure of the group, i.e. pick the cell that is most central (in terms of average squared chi-square distances) to the rest of the group.

If no ordering of the groups can be inferred from experimental labelling of the samples, cellTree first attempts to identify the starting group with the lowest average intra-group distance, based on the biological assumption that intra-group variance would increase as the experiment progresses. Not only is this variance hypothesis confirmed in labelled datasets such as Trapnell et al.’s [7], but also in studies of embryonic cells presented here, where cellTree correctly identified initial stages of development with no further biological input past gene expression and cell grouping (see Results).

However, the MST approach relies to some extent on the assumption that cell distances are uniformly distributed, whereas in fact, we can expect cells inside a batch to have much lower variance than across batches.

The “ideal” structure of a series of cell observations in a differentiation experiment would look like a single path connecting all cells or, in the case of subtype differentiation, a tree with a very small number of branches (one terminal for each differentiated sub-type). Because the samples are in fact physically different, rather than the evolution of a single cell, we must expect small variations around such a theorised continuum. Our suggested approach is to identify cells that are most representative (at the gene expression level) of the biological continuum: a “backbone”, such that all remaining cells in the experiments are similar enough to a representative in that backbone. Hence the following definition:

Given a set of vertices *V* and a pairwise-distance function $d:V\times V\to {\mathbb{R}}^{+}$, we call *backbone tree* a tree *T*, such that:

- *T* is a tree with vertices *V* and edges *E*.
- Its backbone *B* is a subtree of *T* with vertices *V*_*B*_⊆*V* and edges *E*_*B*_⊆*E*.
- All vertices of *T* in *V*∖*V*_*B*_ (the ‘vertebrae’) are less than distance *δ* to at least one vertex in the backbone tree *B*: ∀*v*∈*V*∖*V*_*B*_,∃*v*_*B*_∈*V*_*B*_ such that *d*(*v*,*v*_*b*_)≤*δ*.
- All ‘vertebrae’ vertices of *T* (*v*∈*V*∖*V*_*B*_) are connected by a single edge to the closest vertex in the backbone tree: $\forall v \in V \setminus V_{B}, \forall v' \in V: (v, v') \in E \iff v' = \text {argmin}_{v' \in V_{B}} d(v, v')\phantom {\dot {i}\!}$.

The choice of the parameter *δ* (the backbone tree “width”) of course greatly affects the resulting backbone tree optimisation, and may require adjustment depending on expectations over the structure of the cells (e.g. as a single linear path, or a tree with multiple branches). In order to find a good estimate for *δ*, we look at the probability density function of pairwise distances (using a kernel density estimation), and select the first mode of the distance distribution if it exists.

Additionally, it is generally desirable to relax the last condition of the definition by allowing a proportions of outliers that are at distance >*δ* from any vertices in *V*_*B*_.

Using the above definition, we can define an optimal backbone tree, *T*^∗^, as a backbone tree that minimises the sum of weighted edges in its backbone subtree:

$$ T^{*} = \text{argmin}_{T} \sum\limits_{e \in E_{B}} d(e) $$

Such an optimal backbone tree aims to give a clear hierarchical representation of the cells relationship: the objective function puts pressure on finding a (small) group of prominent cells (the backbone) that are good representatives of the major stages in the cell’s biological process (over time or space), while redundant cells that closely resemble a chosen representative are ignored.

Finding an optimal solution to this problem is unfortunately NP-Complete (shown, for example, by reduction to the Vertex Cover problem or rectilinear Steiner tree problem [18]), but we propose a fast heuristic relying on the MST that produces a close approximation (see Additional file 1 for algorithm in pseudocode).

### Analysing topics with gene ontology terms enrichment

Because of their Bayesian mixture nature, ‘topics’ obtained through LDA fitting do not always match clear and coherent groupings (biological or otherwise), subject to the sparsity of the model and complexity of the input data. In particular, less sparse models (with higher number of topics) may lead to better cell distance computation, but be harder to interpret.

In most cases, however, enrichment analysis of per-topic gene distribution can help characterise a given topic and its role in the cell’s process, and even provide potential biological insight, by outlining the general processes most active in specific sections of the cell tree.

Topic analysis is conducted using Gene Ontologies: testing for terms that are significantly enriched within a topic. First, cellTree orders genes for each topic by their per-topic probability, then applies a Kolmogorov-Smirnov test to compute a p-value for each of the GO terms associated with the ordering, using the *weight* algorithm presented in [19] to account for graph relationship between terms. Bonferonni-corrected significant p-values can then be used a tool for identifying the biological meaning of each topic.

Because of the statistical nature of LDA models, a fair amount of overlap exists between the genes assigned to each topic (in particular for genes at the lower end of the probability distribution). In order to identify the biological specificity of each topic, it is therefore helpful to study GO terms that are either unique to a given topic or appear in a minority of topics (cellTree presents both exhaustive and topic-specific lists of GO term for each topic).

## Results

A major obstacle in obtaining quantitative comparison metrics for single-cell ordering methods, is the difficult to establish a gold standard for cell ordering annotation: the large number of misattributed cells in standard time-series experiments is one of the driving factor behind the need for such tools in the first place. To address this issue, we used a set of single-cell measurement taken during the embryonic development of *Mus musculus* [20]: the ability to assess development stages visually in such instance provides some level of guarantee on chronological labelling.

In order to further evaluate the performance of cellTree and demonstrate its ability to infer biologically-motivated dynamic models of cell populations out of gene expression data, we applied it to a wide range of publicly available single-cell RNA-seq datasets, showing our results alongside existing tools when possible.

Although all results are presented here using visual backbone tree plots, a more detailed tabular format, with full lists of ordered cells for each branch, is available as Additional file 2.

### Comparison to other methods

For comparison, we selected two of the most prominent tools currently publicly available to treat high-dimensional single-cell data, each representative of a different dimension-reduction approach: Monocle [7], which uses ICA, and TSCAN [21], which relies on PCA.

Other single-cell data analysis tools which focus exclusively on low-dimensional mass cytometry data (such as Wanderlust [22]) fell outside the scope of our method and were not considered. The Sincell package [6], which presents a general framework to treat single-cell data, but mainly relies on ICA or PCA for dimension reduction, was also excluded from this comparison.

To provide a baseline comparison, we ran a naive approach using a Travelling Salesman Problem (TSP) algorithm to compute a cell ordering that approximatively minimises the total euclidean distances between each vector of gene expressions. Once a tour has been found, the starting group, provided as a parameter, is used to shift the tour as needed, and the ordering is reversed if necessary, much in the same way that Monocle and TSCAN function (cellTree does not require such manual input and tries to infer them from the data itself).

Results were measured in terms of accuracy over all pairwise combination of cells between the candidate ordering and perfect ordering and are shown in Table 1. From a data set of 90 cells with 20,214 gene levels each, all methods start with a coarse thresholding (removing genes with low levels of expression), resulting in a smaller effective number of genes for use by the statistical model (“Input size after thresholding”). For the TSP-based approach, results were averaged over 100 iterations.

**Table 1:** Comparison of single-cell gene expression ordering tools, using mouse embryo data

Tool	Naive	Monocle	TSCAN	cellTree
Method	TSP	ICA	PCA	LDA
Input size after thresholding	20,214	10,452	225	12,903
Accuracy in %	90.6	94.8	95.3	96.5
CPU time in seconds	0.015	5308.6	0.08	18.5

As can be seen, not only does cellTree outperform all other methods on this task, but it is many orders of magnitude faster than Monocle, which requires upward of an hour to proceed with its dimension-reduction step: this step could be greatly sped-up by providing a set of known marker genes, but it is assumed here that an experimentalist might not have such knowledge about the data. It is also worth noticing that, while TSCAN performs data-reduction much faster, it starts with a much-reduced input of 225 genes (obtained by thresholding the initial set), before proceeding with PCA. This drastic thresholding may result in a loss of information, especially in genes that are only active in a small subset of the cells.

Although matters of visualisation techniques are essentially subjective and difficult to evaluate quantatively, we surmise that our suggested visualisation as a backbone tree provides the clearest overview of the cell hierarchy.

Importantly, as illustrated in the next sections, cellTree’s superior accuracy is not obtained at the expense of model legibility: unlike other methods, cellTree’s models can be readily analysed to produce useful biological insights about the process studied.

### Myoblast differentiation

Skeletal myoblasts are known to undergo a well-established sequence of morphological and transcriptional changes during differentiation. In their introductory paper for ICA-based single-cell analysis tool Monocle, Trapnell et al. [7] studied the trajectory of 271 single-cell RNA-seq measurements of human myoblasts taken 0, 24, 48 and 72 hours into the differentiating process.

We ran cellTree on the provided dataset, with no further pre-treatment, other than log-normalising the expression values and removing genes with a standard deviation below 0.5 (selecting about 13,500 expression values per cell out of the initial 47,192) and using the 5 topic-model automatically selected by the LDA inference method (see Implementation for a discussion of model selection techniques).

**Fig. 1:**
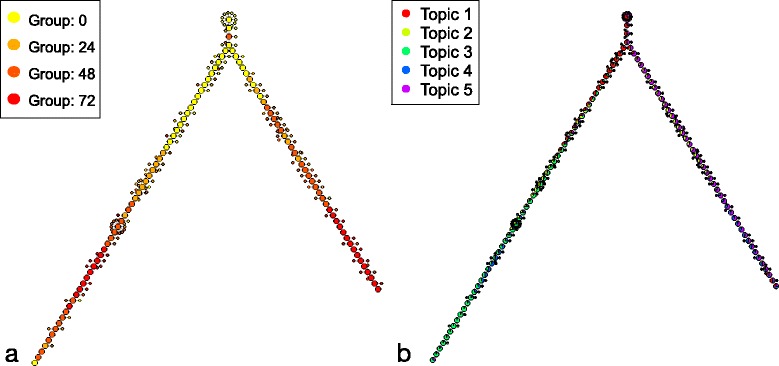
Myoblast Backbone Tree. Backbone tree obtained from the dataset of differentiating myoblasts with cellTree. Larger nodes indicate backbone cells (selected representatives), whereas smaller nodes represent cells that only show slight variations from the backbone cell they are attached to. Cell nodes have been coloured according to **a** their sampling time (in hours from the start of the differentiation process) and **b** their distribution over topics

The backbone tree generated from the results shows a clear two-phase trajectory (see Fig. 1), with a small branch of non-differentiating cells. The ordering of cells along the tree also follows the expected chronological order from time 0 to 72, although we can observe the expanding variance at later stages, previously noted by the authors of the Sincell package in their own analysis of this data [6].

**Fig. 2:**
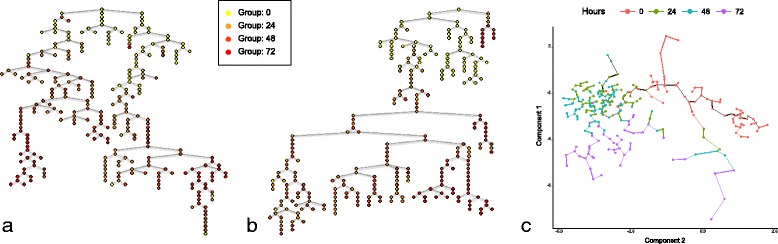
Myoblast MST. Comparison of minimum spanning trees obtained from the dataset of differentiating myoblasts by cellTree (**a**) and Monocle (**b**). Cell nodes have been coloured according to their sampling time (in hours). **c** shows the final tree structure obtained directly by Monocle

The results obtained are qualitatively similar to that of Monocle (see comparison in Fig. 2), although it could be argued that the visualisation as backbone tree used by cellTree is easier to interpret than the type of MST used by Monocle. In stark contrast to other methods, however, not only does cellTree not require any pre-existing knowledge about the cell population (such as the number of expected branches or the position of the tree root), it can in fact attach biological information to the different parts of the cell tree through analysis of the gene expression data.

As can be observed on the version of the tree annotated with topic distributions, undifferentiated cells are dominated by topic 1. A look at GO terms uniquely enriched for that topic (see Tables 2 and 3), reveals a large number of terms indicative of highly mitotic conditions, such as cell division (BP:GO:0051301), mitotic nuclear division (BP:GO:0007067), transcription-coupled excision repair (BP:GO:0006283) and GTPase mediated signal transduction (BP:GO:0007264). Within the first 24 h (topic 2), there is a shift of the myocyte differentiation to more transcriptional processes (CC:GO:005665). At 48 h (topic 4), the myocytes undergo intracellular protein transport (BP:GO:006886). At the 72 h time-point (topic 3) the myocytes undergo protein translational processes, while developing structural component of Z-disks (CC:GO:0030018) and stress fibers (CC:GO:001725) allowing filament sliding (BP:GO:0030049) and MAPKK activity (BP:GO:0000186) for cellular fusion in high-mitogenic conditions. Furthermore, although not unique to one topic (see extended table of significantly enriched GO terms in Additional files 3 and 4), Wnt signalling pathway (BP:GO:0090263), which regulates crucial aspects of cell fate determination [23], appears significantly enriched for topic 3 (*p* = 5.8e-10) and 5 (*p* = 2.5e-11).

**Table 2:** List of biological process go terms significantly enriched and uniquely appearing in each topic for myoblast differentiation

GO.ID	Term	*p*-Value
Topic 1		
GO:0051301	cell division	4.7e-12
GO:0007067	mitotic nuclear division	1.4e-10
GO:0019083	viral transcription	1.7e-10
GO:0006283	transcription-coupled nucleotide-excision repair	5.7e-10
GO:0007264	small GTPase mediated signal transduction	1.7e-09
GO:0007077	mitotic nuclear envelop disassembly	7.8e-09
GO:0008380	RNA splicing	1.0e-08
GO:0016925	protein SUMOylation	1.1e-08
G O:0000086	G2/M transition of mitotic cell cycle	2.7e-08
GO:0007059	chromosome segregation	4.5e-08
GO:0010827	regulation of glucose transport	4.6e-08
GO:0000082	G1/S transition of mitotic cell cycle	6.0e-08
GO:0006369	termination of RNA polymerase II transcription	1.1e-07
GO:0042769	DNA damage response, detection of DNA damage	1.3e-07
GO:1900034	regulation of cellular response to heat	1.4e-07
GO:0006271	DNA strand elongation involved in DNA replication	1.6e-06
GO:0006626	protein targeting to mitochondrion	1.7e-06
Topic 2		
GO:0006367	transcription initiation from RNA polymerase II promoter	9.9e-08
GO:0006376	mRNA splice site selection	1.1e-06
Topic 3		
GO:0030049	muscle filament sliding	2.5e-08
GO:0017148	negative regulation of translation	1.1e-06
GO:0000186	activation of MAPKK activity	1.8e-06
Topic 4		
GO:0006886	intracellular protein transport	9.6e-07
Topic 5		
GO:0050434	positive regulation of viral transcription	3.0e-07
GO:0006370	7-methylguanosine mRNA capping	4.3e-07
GO:0044267	cellular protein metabolic process	4.7e-07
GO:0006457	protein folding	1.6e-06

**Table 3:** List of cellular components go terms significantly enriched and uniquely appearing in each topic for myoblast differentiation

GO.ID	Term	*p*-Value
Topic 1		
GO:0000777	condensed chromosome kinetochore	1.1e-10
GO:0005813	centrosome	1.4e-06
GO:0000922	spindle pole	2.2e-06
GO:0005876	spindle microtubule	3.4e-06
GO:0005689	U12-type spliceosomal complex	4.8e-06
GO:0005688	U6 snRNP	5.7e-06
GO:0000940	condensed chromosome outer kinetochore	9.5e-06
GO:0005759	mitochondrial matrix	1.1e-05
GO:0000784	nuclear chromosome, telomeric region	1.2e-05
GO:0046540	U4/U6 x U5 tri-snRNP complex	1.3e-05
Topic 2		
GO:0005665	DNA-directed RNA polymerase II, core complex	5.1e-06
Topic 3		
GO:0001725	stress fiber	1.7e-07
GO:0030018	Z disc	3.1e-07
GO:0098800	inner mitochondrial membrane protein complex	1.3e-06
GO:0000932	cytoplasmic mRNA processing body	2.9e-06
Topic 4		
GO:0005604	basement membrane	1.0e-05
Topic 5		
GO:0005789	endoplasmic reticulum membrane	2.4e-06
GO:0005885	Arp2/3 protein complex	1.3e-05

The branch of non-differentiating cells is dominated by topic 5, which shows strong enrichment for cellular protein metabolic process (BP:GO:0044267) and 7-me mRNA capping (BP:GO:0006370), but also viral transcriptional regulation (BP:GO:0050434), which may suggest a virus coevolution in the myocytes [24, 25]. This non-differentiated set shows expression of the actin-related Arp2/3 protein complex (CC:GO:0005885), suggesting the potential to further mature to either skeletal or cardiac myocyte. It also regulates the endoplasmic reticulum and plasma membrane junction with proteins such as STIM [EMBL:ENSMUSG00000027498], to control signalling and metabolic processes for the proper regulation of calcium within the striated muscle [25].

**Fig. 3:**
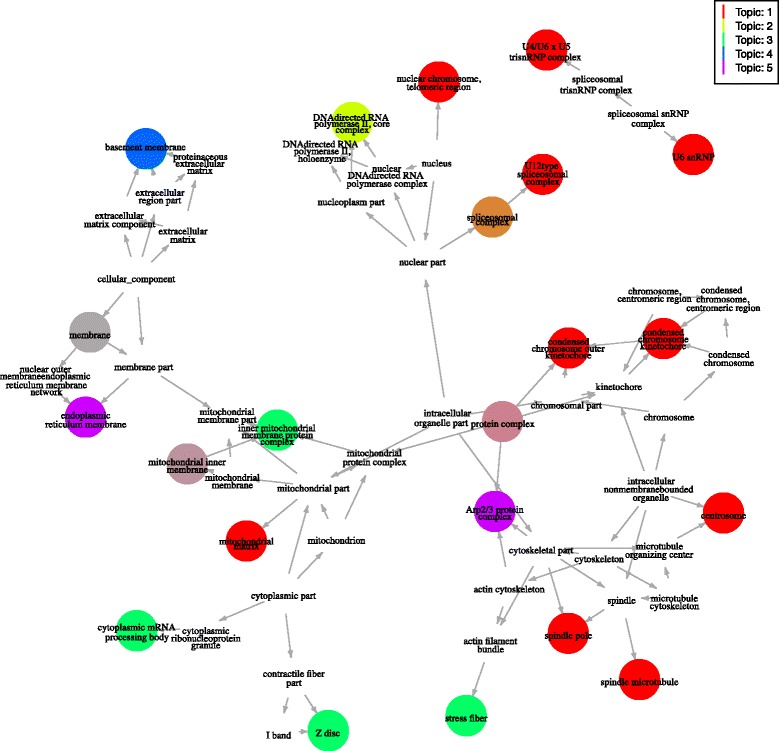
Cellular Component GO Terms for Myoblasts. Subgraph of the Cellular Component GO terms DAG showing terms significantly enriched for each topic. Darker colours indicated lower p-values and terms shared between topics use a combination of the topics colours (terms that are enriched in more than half the topics have been removed)

Significantly enriched GO terms for all topics can be conveniently visualised as a subgraph of the directed acyclic graph (DAG) for Cellular Component GO terms (see Fig. 3).

Without the need for further manual analysis or expert input, cellTree was able to infer and label major stages of the myoblast differentiation process, along with the biological specificities of the set of undifferentiated cells, in line with existing biological knowledge on myoblast differentiation [7].

### Study of embryonic development

In order to highlight the robustness of our approach on highly heterogeneous cell population, we analysed two data sets following embryonic cells at different stages of differentiation: a study profiling lncRNAs of human-induced embryonic stem cells (hESC) [5] and a study of autosomal monoallelic genes from oocyte to blastocytes in *Mus musculus* [20]. We show that cellTree facilitates the identification of physiologically meaningful subpopulations that clearly define the continuum along the process of differentiation.

Both datasets were analysed with four topics, privileging a sparse model with clearly delineated topics, over a more complex model with more topics, which may better explain the cells relationship but present more functional overlap between topics (note that because the two models were fitted and analysed independently, there is no correspondence between topic numbers in either model).

**Fig. 4:**
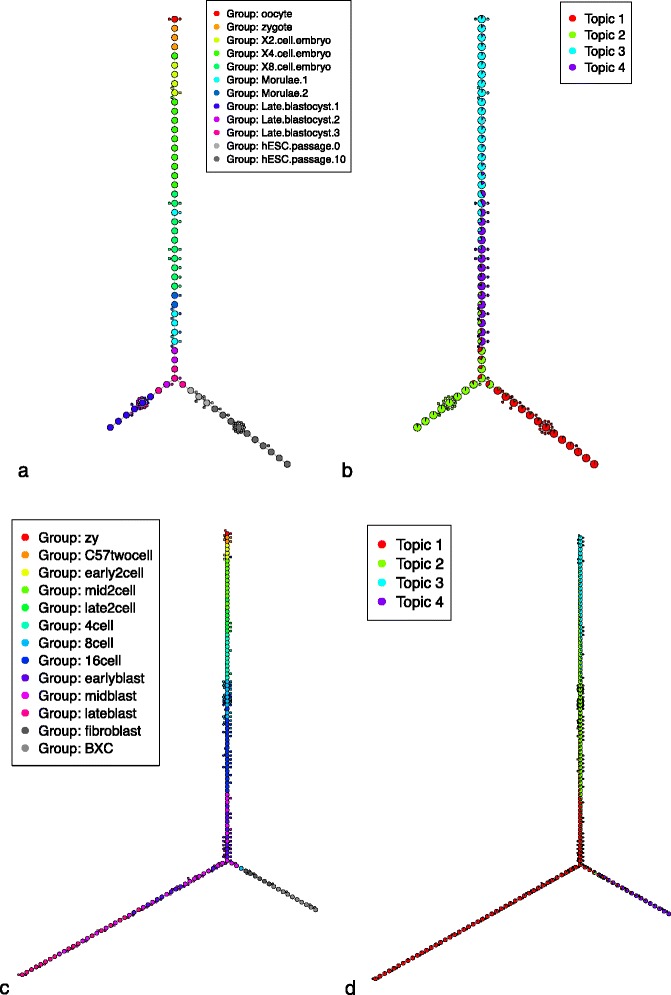
Embryonic cell differentiation. Backbone trees obtained by cellTree for hESC (**a** and **b**) and mouse embryo cell differentiation (**c** and **d**). Both trees are shown annotated with cell type (**a** and **c**) and topic distributions (**b** and **d**)

In both cases, we observe that the differentiation continuum was overall consistent with general biological knowledge and published analysis of the data: the trees (see Fig. 4) show a sequential ordering of cellular stages by their progress through embryonic cell development in a linear fashion (a full tabular list of the cells ordered by cellTree can be found in Additional file 5).

For the hESC data [5], the authors noted that stage-specific lncRNA expression patterns emphasise the critical role it plays in development. In other words, maternal-inherited lncRNA dominates early stages, before decreasing as the embryo develops, which suggests the critical role hESC-specific lncRNAs play in pluripotency maintenance till the morulae stage [26]. In agreement with this analysis [5], our model shows oocytes and zygotes clustered together in the same development stage (dominated by topic 3), while X2, X4, and X8 cells are clustered under topic 4.

By looking at the GO analysis for the different topics using terms for Biological Processes (BP, see Tables 4 and 5 and Additional file 6 for extended table), Cellular Components (CC, see Table 6 and Additional file 7) and Molecular Function (MF, see Tables 7 and 8) and Additional file 8), we can identify with very good accuracy the successive stages of differentiation at a whole-genome scale:

**Table 4:** List of biological process GO terms significantly enriched and uniquely appearing in each topic for hESC differentiation

GO.ID	Term	*p*-Value
Topic 1		
GO:0042769	DNA damage response, detection of DNA	3.5e-08
	damage	
GO:0090263	positive regulation of canonical Wnt	3.0e-07
	signaling pathway	
GO:0045814	negative regulation of gene expression,	4.1e-07
	epigenetic	
GO:0051084	*de novo* posttranslational protein folding	1.3e-06
Topic 2		
GO:1900034	regulation of cellular response to heat	7.0e-08
GO:0051301	cell division	1.3e-07
GO:0044743	intracellular protein transmembrane import	2.1e-07
GO:0007077	mitotic nuclear envelope disassembly	7.2e-07
GO:1990542	mitochondrial transmembrane transport	9.7e-07
Topic 3		
GO:0000281	mitotic cytokinesis	1.3e-07
Topic 4		
GO:0010501	RNA secondary structure unwinding	1.5e-09
GO:0045596	negative regulation of cell differentiation	9.5e-09
GO:0048387	negative regulation of retinoic acid receptor	1.9e-08
	signaling pathway	
GO:0043066	negative regulation of apoptotic process	1.4e-07
GO:0006368	transcription elongation from RNA	2.0e-07
	polymerase II promoter

**Table 5:** List of cellular components GO terms significantly enriched and uniquely appearing in each topic for embryonic mouse cell differentiation

GO.ID	Term	*p*-Value
Topic 1		
GO:0033178	proton-transporting two-sector ATPase complex, catalytic domain	7.8e-07
GO:0031597	cytosolic proteasome complex	2.0e-06
GO:0005680	anaphase-promoting complex	2.5e-06
GO:0008540	proteasome regulatory particle, base subcomplex	3.7e-06
Topic 2		
GO:0005736	DNA-directed RNA polymerase I complex	4.8e-07
GO:0005665	DNA-directed RNA polymerase II, core complex	5.0e-07
GO:0005844	polysome	1.2e-06
GO:0005689	U12-type spliceosomal complex	1.8e-06
GO:0005838	proteasome regulatory particle	3.1e-06
GO:0008023	transcription elongation factor complex	8.1e-06
GO:0000346	transcription export complex	9.5e-06
GO:0015030	Cajal body	1.5e-05
Topic 3		
GO:0005813	centrosome	4.1e-08
GO:0000932	cytoplasmic mRNA processing body	3.1e-06
GO:0016592	mediator complex	6.2e-06
Topic 4		
GO:0072562	blood microparticle	7.4e-29
GO:0005783	endoplasmic reticulum	9.3e-27
GO:0005615	extracellular space	2.5e-25
GO:0005743	mitochondrial inner membrane	3.7e-22
GO:0005829	cytosol	2.2e-17
GO:0005759	mitochondrial matrix	2.0e-11
GO:0016021	integral component of membrane	3.2e-10
GO:0034364	high-density lipoprotein particle	3.7e-09
GO:0031012	extracellular matrix	4.1e-09
GO:0030176	integral component of endoplasmic reticulum membrane	4.2e-09
GO:0005777	peroxisome	3.3e-08
GO:0005789	endoplasmic reticulum membrane	5.4e-08
GO:0005788	endoplasmic reticulum lumen	6.5e-08
GO:0034361	very-low-density lipoprotein particle	1.3e-07
GO:0005764	lysosome	1.4e-07
GO:0005791	rough endoplasmic reticulum	3.6e-07
GO:0005778	peroxisomal membrane	1.2e-06
GO:0005793	endoplasmic reticulum-Golgi intermediate compartment	1.3e-06
GO:0070069	cytochrome complex	3.8e-06
GO:0009986	cell surface	5.1e-06
GO:0030867	rough endoplasmic reticulum membrane	8.9e-06
GO:0005782	peroxisomal matrix	9.8e-06
GO:0009897	external side of plasma membrane	1.0e-05
GO:0005790	smooth endoplasmic reticulum	1.1e-05
GO:0005765	lysosomal membrane	1.1e-05

**Table 6:** List of cellular components GO terms significantly enriched and uniquely appearing in each topic for hESC differentiation

GO.ID	Term	*p*-Value
Topic 1		
GO:0044297	cell body	2.7e-06
GO:0045263	proton-transporting ATP synthase complex, coupling factor F(o)	4.2e-06
GO:0005819	spindle	1.3e-05
Topic 2		
GO:0005789	endoplasmic reticulum membrane	7.6e-08
GO:0098796	membrane protein complex	3.6e-07
GO:0005746	mitochondrial respiratory chain	5.6e-07
GO:0000785	chromatin	5.5e-06
GO:0042645	mitochondrial nucleoid	1.2e-05
Topic 3		
GO:0005813	centrosome	2.8e-06
Topic 4		
GO:0005634	nucleus	3.9e-15
GO:0071339	MLL1 complex	4.5e-06
GO:0005689	U12-type spliceosomal complex	9.1e-06
GO:0005732	small nucleolar ribonucleoprotein complex	1.1e-05

**Table 7:** List of molecular function GO terms significantly enriched and uniquely appearing in each topic for hESC differentiation

GO.ID	Term	*p*-Value
Topic 1		
GO:0004129	cytochrome-c oxidase activity	7.5e-06
Topic 2		
GO:0008536	Ran GTPase binding	3.2e-06
Topic 4		
GO:0003677	DNA binding	4.4e-09
GO:0030515	snoRNA binding	1.5e-07
GO:0004004	ATP-dependent RNA helicase activity	3.0e-07
GO:0042974	retinoic acid receptor binding	1.1e-06
GO:0004402	histone acetyltransferase activity	6.6e-06
GO:0043022	ribosome binding	6.9e-06

**Table 8:** List of biological process GO terms significantly enriched and uniquely appearing in each topic for embryonic mouse cell differentiation

GO.ID	Term	*p*-Value
Topic 1		
GO:0032543	mitochondrial translation	2.2e-07
GO:0015031	protein transport	2.7e-07
GO:0015986	ATP synthesis coupled proton transport	1.8e-06
Topic 2		
GO:0042254	ribosome biogenesis	7.1e-10
GO:0000462	maturation of SSU-rRNA from tricistronic rRNA transcript (SSU-rRNA, 5.8S rRNA, LSU-rRNA)	7.2e-10
GO:0042273	ribosomal large subunit biogenesis	4.1e-08
Topic 3		
GO:0007049	cell cycle	1.6e-09
GO:0045893	positive regulation of transcription, DNA-templated	1.5e-08
GO:0006355	regulation of transcription, DNA-templated	1.2e-07
GO:0000122	negative regulation of transcription from RNA polymerase II promoter	3.2e-07
GO:0043161	proteasome-mediated ubiquitin-dependent protein catabolic process	5.5e-07
GO:0000281	mitotic cytokinesis	1.5e-06
Topic 4		
GO:0055114	oxidation-reduction process	< 1e-30
GO:0006805	xenobiotic metabolic process	3.5e-13
GO:0042738	exogenous drug catabolic process	1.9e-12
GO:0006749	glutathione metabolic process	4.3e-12
GO:1901606	alpha-amino acid catabolic process	6.9e-10
GO:0046700	heterocycle catabolic process	9.8e-10
GO:0008203	cholesterol metabolic process	1.0e-09
GO:0019373	epoxygenase P450 pathway	7.6e-09
GO:0044270	cellular nitrogen compound catabolic process	1.0e-08
GO:0006958	complement activation, classical pathway	1.2e-08
GO:0006641	triglyceride metabolic process	4.2e-08
GO:0045454	cell redox homeostasis	4.4e-08
GO:0019439	aromatic compound catabolic process	6.0e-08
GO:1901361	organic cyclic compound catabolic process	7.3e-08
GO:0030433	ER-associated ubiquitin-dependent protein catabolic process	1.2e-07
GO:0006953	acute-phase response	1.4e-07
GO:0010951	negative regulation of endopeptidase activity	2.7e-07
GO:0042493	response to drug	5.4e-07
GO:0006103	2-oxoglutarate metabolic process	5.8e-07
GO:0042537	benzene-containing compound metabolic process	6.2e-07
GO:0010466	negative regulation of peptidase activity	9.0e-07
GO:0019748	secondary metabolic process	9.8e-07
GO:0006120	mitochondrial electron transport, NADH to ubiquinone	1.0e-06
GO:0042744	hydrogen peroxide catabolic process	1.0e-06
GO:0009813	flavonoid biosynthetic process	1.4e-06
GO:0052696	flavonoid glucuronidation	1.4e-06
GO:0046688	response to copper ion	1.5e-06

Oocytes progress into stages of differentiated cell types in a process that requires cellular components to regulate gene expression patterns appropriately [27]. GO terms for topic 3, indeed show mitosis (BP:GO:0000281) as its dominating biological process.

Looking directly at the top genes in the distribution for topic 3 (see Additional file 9) also highlights oocytes’ unique ability to remodel the chromatin to closely coordinate the cellular and chromosomal events of oogenesis: ACTB [EMBL:ENSG00000075624], PTMA [EMBL:ENSG00000187514], RPS8 [EMBL:ENSG00000142937], RPL19 [EMBL:ENSG00000108298], RPS7 [EMBL:ENSG00000171863], SPL41 [EMBL:ENST00000552314] and RPL23 [EMBL:ENSG00000125691] are all involved in several transcription regulatory factors that are regulators of ribosome biogenesis and protein synthesis.

As development progresses to the morulae stage (topic 4), we see a strong enrichment for nuclear ribonucleoproteins (CC:GO:0005732) such as the MLL1 complex (CC:GO:0071339) which activate spliceosomes (CC:GO:0005689) [28].

In agreement with the published analysis, cellTree properly segregates the blastocyst 1 trophectoderm layer (TE, in the late blastocyst branch) from the inner cell mass (ICM, in the hESC passage branch), emphasising the critical differentiation stage of the morulae 1 embryo.

Looking at the topic distributions around the point of separation between TE and ICM, we see the contribution of topic 1: initiating self-renewal and pluripotency in blastocysts 1 by developing the endogenous extracellular matrix [29]. Due to the intensive restructuring, the critical demand that drives mitochondrial activity is met by ATP synthases (GO:0045263). FBXO5 [EMBL:ENSG00000112029], DIDO1 [EMBL:ENSG00000101191], PSRC1 [EMBL:ENSG00000134222], PPP2R3C [EMBL:ENSG00000092020] are responsible for the nucleosome structure of the chromosomal spindles (GO:0005819) to remodel the nucleosome. STAU1 transports and localizes mRNA to different subcellular compartments [30, 31], while RPL28 [EMBL:ENSMODG00000000275] is required for the regulation of transcription within the cell body (CC:GO:0044297) to regulate stem cell pluripotency and neoplastic progression [32].

In agreement with the published analysis, passage 0 hESC cells are closest to the blastocysts, emphasising the gene expression landscape of hESC derivation, defined by the different stages of blastocyte 3 epiblasts (EPI) within the outgrowth of hESCs [33], highlighting the continuum of hESC development in contrast to simple hierarchical clustering. Although passage 0 and 10 of hESC outgrowth are in the same branch of the tree, as could be expected since only 4.6 % (861/18383) of the genes show differential expression between the two outgrowth passages, cellTree correctly identifies the two groups where the initial publication was unable to obtain that level of clustering [34].

The initial publication’s authors report the possibility of misclassification within the inner mass portion (ICM) of the blastocysts, which can result in some late blastocyte stage cells labeled as TE cells, which accounts for the lack of uniformity within these groups in the structure inferred by cellTree. The similarity between the ICM lineages of PE and EPI, as indicated by blastocytes 2 and 3 respectively, can be observed within the tree structure. Throughout the development process, MTRNR2L2, GAPDH, MTRNR2L9, and PTMA are all neuroprotective proteins that stabilise the cell during its reconstructive development.

The trees obtained for mouse embryonic cell development [20] present very similar topic enrichment:

The maternal-zygotic transition dominates at the 2-cell stage (topic 3), indicated by the initiation of the mitotic processes (BP:GO:0006355) that leads to the gradual of maternal DNA. Cell cycle processes (BP:GO:0007049) include ubiquitinone-specific proteases (MF:GO:0004843) and the mediator complex (CC:GO:0016592) and inhibit apoptotic processes [26] for the maternal-zygotic transition.

In the mid and late 2-cell stage (topic 2), the decline of high-mitogen conditions is coupled with an increase in ribosome biogenesis (BP:GO:0042254) which mitigates snoRNA assembly (MF:GO:0030515), and transcriptional regulation of spliceosomes (CC:GO:0005689) and proteosomes (CC:GO:0005838).

Finally, topic 1 is mainly associated with the translation processes (BP:GO:0006412) and transport processes (BP:GO:0015986) of the blastocytes, while the maturity of fibroblast and liver cell controls (BXC) is clearly visible in topic 4’s association to metabolic and catabolic processes, such as xenobiotic metabolic process (BP:GO:0006805) and glutathione metabolic process (BP:GO:0006749), DNA methylation patterns (MF:GO:000839) and receptor ligand binding (MF:GO:0020037).

As we can see from this GO analysis of the gene models used, the sequential developmental process of the cells is perfectly aligned with the topics found by cellTree, and regions that are susceptible to perturbation during embryo differentiation are highlighted by the topic distributions.

**Fig. 5:**
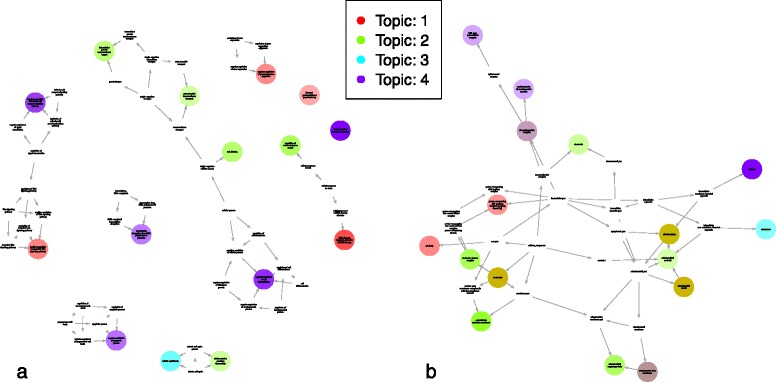
Enriched GO terms subgraph for hESC. Subgraph of the GO DAG showing terms significantly enriched for each topic in hESC differentiation: **a** using BP GO terms. **b** using CC GO terms

Although focussing on terms uniquely enriched for each topic is generally sufficient to link each of the model’s topic to a clearly delineated type of cellular or molecular activity, especially when the general subtype of cell is already known, it is also possible to look at a more comprehensive list of enriched terms that allow overlap between topics to gain new biological insights (see table of rare, but non-unique, significantly enriched GO terms for hESC in Additional files 6, 7 and 8). There too, a good overview of each topic’s role in the differentiation can be obtained by looking at GO subgraphs (see Fig. 5).

### Cortical cells

Although cellTree is typically best suited to handle cell differentiation data over time, we also show that it can reveal interesting insights about latent subtypes in heterogeneous cell populations.

Deciphering the cellular taxonomy of brain cells is still a work in progress [35]. Neuronal diversity is supported by functional complexity, as overlapping characteristics of interneuron subtypes would otherwise be difficult to explain, such as is the case with interneuron diversity within the rostrocaudal axis in *Mus musculus* [36].

We applied cellTree to single-cell RNA-seq measurements of mouse cortical cells [37] to see if it could help deciphering cells subtypes based on genomic profiles alone.

**Fig. 6:**
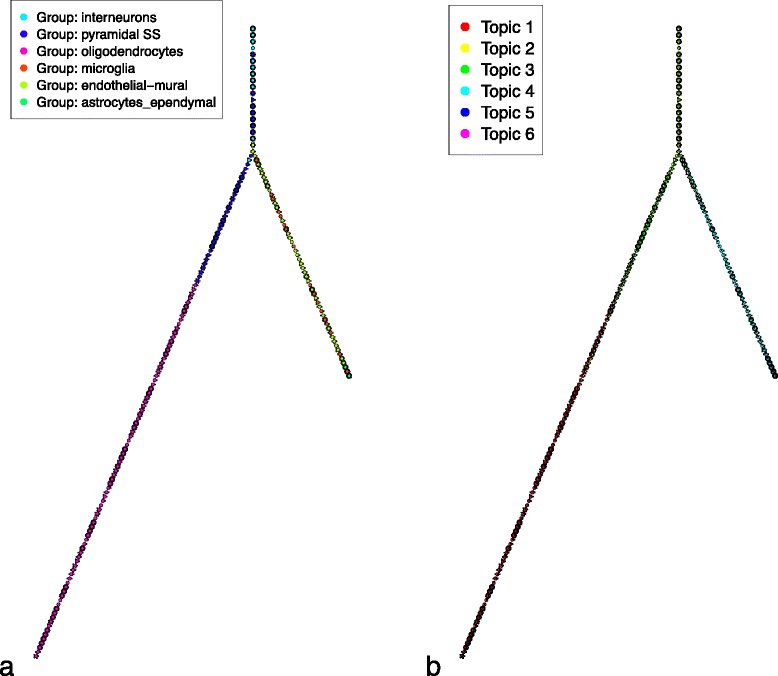
Mouse cortical cell subtypes. Backbone trees obtained by cellTree for mouse cortical cell subtypes annotated with cell subtype (**a**) and topic distributions (**b**)

The resulting backbone tree (see Fig. 6 and Additional files 10 and 11 for full ranking of cells) overall shows a structure in line with known subtype labels in the dataset and enriched GO terms (see Tables 9, 10 and 11) deliver biologically-coherent explanations for each topic.

**Table 9:** List of molecular function GO terms significantly enriched and uniquely appearing in each topic for embryonic mouse cell differentiation

GO.ID	Term	*p*-Value
Topic 2		
GO:0001054	RNA polymerase I activity	2.5e-07
GO:0043022	ribosome binding	4.8e-06
GO:0030515	snoRNA binding	5.0e-06
GO:0005524	ATP binding	6.1e-06
Topic 3		
GO:0003713	transcription coactivator activity	7.6e-07
GO:0003730	mRNA 3’-UTR binding	4.6e-06
GO:0004843	ubiquitin-specific protease activity	7.7e-06
Topic 4		
GO:0020037	heme binding	2.2e-19
O:0008395	steroid hydroxylase activity	5.9e-18
O:0019825	oxygen binding	6.5e-14
O:0005506	iron ion binding	9.2e-13
O:0004364	glutathione transferase activity	1.5e-12
O:0008392	arachidonic acid epoxygenase activity	1.9e-12
O:0005102	receptor binding	4.2e-11
GO:0004867	serine-type endopeptidase inhibitor activity	1.1e-10
GO:0009055	electron carrier activity	1.3e-10
GO:0070330	aromatase activity	2.6e-10
GO:0042803	protein homodimerization activity	7.4e-10
GO:0008201	heparin binding	1.5e-09
GO:0002020	protease binding	2.5e-08
GO:0051087	chaperone binding	6.0e-07
GO:0003988	acetyl-CoA C-acyltransferase activity	8.4e-07
GO:0016836	hydro-lyase activity	1.0e-06
GO:0004602	glutathione peroxidase activity	1.0e-06
GO:0004601	peroxidase activity	1.3e-06
GO:0030170	pyridoxal phosphate binding	1.6e-06
GO:0004029	aldehyde dehydrogenase (NAD) activity	2.4e-06
GO:0001848	complement binding	3.1e-06
GO:0016616	oxidoreductase activity, acting on the CH-OH group of donors, NAD or NADP as acceptor	3.2e-06
GO:0050660	flavin adenine dinucleotide binding	3.8e-06
GO:0005507	copper ion binding	4.1e-06
GO:0016709	oxidoreductase activity, acting on paired donors, with incorporation or reduction of molecular oxygen, NAD(P)H as one donor, and incorporation of one atom of oxygen	5.3e-06
GO:0032403	protein complex binding	5.7e-06
GO:0051537	2 iron, 2 sulfur cluster binding	7.8e-06

**Table 10:** List of molecular function GO terms significantly enriched and uniquely appearing in each topic for mouse cortical cell subtypes

GO.ID	Term	*p*-Value
Topic 2		
GO:0005524	ATP binding	4.7e-12
GO:0005516	calmodulin binding	4.6e-09
GO:0019901	protein kinase binding	2.0e-08
GO:0044325	ion channel binding	3.3e-08
GO:0005515	protein binding	2.7e-07
GO:0005509	calcium ion binding	3.6e-07
GO:0017075	syntaxin-1 binding	1.0e-05
Topic 3		
GO:0003677	DNA binding	1.1e-07
GO:0032403	protein complex binding	3.7e-06
GO:0019843	rRNA binding	6.1e-06
Topic 4		
GO:0003924	GTPase activity	2.7e-07

**Table 11:** List of biological process GO terms significantly enriched and uniquely appearing in each topic for mouse cortical cell subtypes

GO.ID	Term	*p*-Value
Topic 1		
GO:0006457	protein folding	1.1e-06
Topic 2		
GO:0007165	signal transduction	1.8e-06
GO:0016079	synaptic vesicle exocytosis	2.9e-06
Topic 3		
GO:0015991	ATP hydrolysis coupled proton transport	3.0e-07
Topic 4		
GO:0046916	cellular transition metal ion homeostasis	1.4e-06
GO:0016525	negative regulation of angiogenesis	2.7e-06
Topic 5		
GO:0006633	fatty acid biosynthetic process	1.2e-05

The combined effect of topic 2’s decrease and topic 3’s increase matches the biological activity expected in a progression from interneurons to pyramidal cells: as interneurons differentiate into pyramidal neurons, they move away from synaptic vesicle exocytosis (BP:GO:0016079) to a more calcium-based and electrical signalling through ATP hydrolysis (BP:GO:0015991) and calmodulin regulation of neurites’ tips by syntaxin-1 (MF:GO:0017075), which binds to the plasma membranes. This is aligned with studies showing that interneurons and pyramidal cells are both derived from progenitor neocortical cells [38, 39].

The increasing importance of topic 1 in the left branch corresponds to the appearance of more prominent structural features in myelinated neuronal cell types, turning protuberances from the triangular shaped soma of the pyramidal neurons [40] into the branching protrusions of the oligodendrocytes [41, 42]: the excessive myelin glycoprotein present in oligodendrocytes membranes is reflected by the significant protein folding (BP:GO:0006457) activity.

The other branch comprised mostly of non-myelinated glial cells, such as microglia and astrocytes, is dominated by topic 4. Defining functional features of topic 4, such as oxidation reduction processes (BP:GO:0055114) and transport (BP:GO:0006810) in the blood vasculature appropriately outline the structural closeness of epidermal mural cells and microglia [29,30], along with more specific roles such as the participation in metal ion homeostasis (BP:GO:0046916) of endothelial mural cells and negative regulation of angiogenesis (BP:GO:0016525) usually observed in epithelial mural cells [43].

Astrocytes are clustered by cellTree at the end of the differentiation spectrum in the non-myelinated branch, in line with the similarities in vasculature linkage functionality between these neuronal subtypes. Topic 6 accurately marks the stimulated proliferation at the astrocyte stage [44] with the poly(A) RNA binding process (BP:GO:0044822).

Through gene expression values alone, cellTree not only automatically identified myelination as one of the major differentiating characteristic within glial cells: splitting oligodendrocytes and non-myelinated glial cells into two separate branches, it also identified many of the major structural and functional features linking different cortical cell subtypes.

## Conclusions

With cellTree, we introduced an entirely novel approach to single-cell gene expression analysis that not only can infer complex underlying hierarchical structures in cell populations from expression data alone, but also provide biological backing for the model it creates. The representation of cells as statistical mixture of topics allows for the capture of subtle evolving characteristics between cells along a continuum, and deals well with heterogeneous populations.

Although rooted in a strong Bayesian statistical framework, the package is designed to be useable by experimentalists with only minimal bioinformatics skills and absolutely no knowledge in machine learning. Using data meta-analysis, the package can provide reasonable default values for most of the parameters used by the model inference, visualisation and analysis algorithms, making it possible for an unfamiliar user of the software to quickly evaluate a new dataset in a few simple lines of R code. Finally, in addition to letting users manipulate the results as standard R objects, all graph visualisation, ranking tables and result summaries can be rendered to file in PDF or LATE Xformat, for easy reuse in scientific communication.

As with most machine learning models, model complexity is a crucial aspect of the LDA techniques used by cellTree: while denser models (using more topics and a “flater” per-topic distribution over the genes) may yield a better distance matrix between cells (and lead to a more accurate hierarchical structure inference), they are also harder to interpret and subject to the risk of overfitting. A balance must therefore be found with models that produce biologically-useful results, yet remain sparse enough to avoid overfitting and maximise clarity.

In addition to the likelihood-based model selection method [16] currently used by cellTree, we are hoping to offer improved approches for automatic model selection, based on recent advances in topic modelling [45–47] in our next release. Similarly, we are planning to take advantage of recent improvements to the field, to offer a more comprehensive semantic analysis of topics [48, 49].

We also plan to refine the GO enrichment method used by cellTree by taking advantage of techniques for better multiple-hypotheses testing correction [50] and replacing the current rank-based test on the per-topic gene list by statistical testing of the probability distribution itself.

The next step in our development roadmap is the addition of a number of standard methods for both dimensionality-reduction (such as PCA and ICA) and population structure inference, in addition to the topic-based methods already implemented, in order to offer experimentalists an integrated interface to quickly run and compare different analysis pipelines on their single-cell gene expression dataset.

## Availability and requirements

The cellTree R/Bioconductor package is available for free under an open-source licence and can be easily obtained and installed by following the instructions at: http://bioconductor.org/packages/cellTree/ It is designed to run on any recent personal computer (minimum CPU requirements depend on the size of the input data) and any Operating System, as long as the necessary R language software has been installed.

## Additional files

Additional file 1 Approximate Backbone Tree Construction Algorithm. Pseudo-code of the algorithm and heuristics used by cellTree to build a Backbone Tree representation of the cell population. (PDF 102 kb)

Additional file 2 Gene probability distribution for topic 3. Per-topic gene probability distribution for Topic 3 of the hESC data model computed by cellTree. (PDF 56 kb)

Additional file 3 GO BP Terms for myoblast data. Full table of enriched GO BP terms for each topic in myoblast data. (PDF 36 kb)

Additional file 4 GO CC Terms for myoblast data. Full table of enriched GO CC terms for each topic in myoblast data. (PDF 38 kb)

Additional file 5 cellTree summary for hESC data. Full list of cell samples in the hESC data set, ordered and annotated by cellTree. (PDF 36 kb)

Additional file 6 GO BP Terms for hESC data. Full table of enriched GO BP terms for each topic in hESC data. (PDF 42 kb)

Additional file 7 GO CC Terms for hESC data. Full table of enriched GO CC terms for each topic in hESC data. (PDF 35 kb)

Additional file 8 GO MF Terms for hESC data. Full table of enriched GO MF terms for each topic in hESC data. (PDF 31 kb)

Additional file 9 Gene probability distribution for topic 3. Per-topic gene probability distribution for Topic 3 of the hESC data model computed by cellTree. (CSV 3 kb)

Additional file 10 cellTree summary for mouse embryonic cells data. Full list of cell samples in the mouse embryonic cells data set, ordered and annotated by cellTree. (PDF 59 kb)

Additional file 11 cellTree summary for mouse cortical cells data. Full list of cell samples in the mouse cortical cells data set, ordered and annotated by cellTree. (PDF 150 kb)
