# Supplementary material for: CellTree: an R/bioconductor package to infer the hierarchical structure of cell populations from single-cell RNA-seq data
Source: BMC Bioinformatics. 2016 Sep 13;17(1):363. doi: 10.1186/s12859-016-1175-6 (PMC5020541; doi:10.1186/s12859-016-1175-6)
Supplement: Additional file 1 — Approximate Backbone Tree Construction Algorithm. Pseudo-code of the algorithm and heuristics used by cellTree to build a Backbone Tree representation of the cell population. (PDF 102 kb) [file 12859_2016_1175_MOESM1_ESM.pdf]

Approximate Backbone Tree Algorithm

Input: Complete undirected graph: $G \equiv (V(G), E(G))$
Input: Distance attribute: $d : V(G) \times V(G) \rightarrow \mathbb{R}^+$
Input: Distance threshold: δ
Input: Outlier tolerance: ρ
$T := \mathit{minimum\_spanning\_tree}(G)$
$B := \mathit{Graph}(\{T.\mathit{root}\}, \emptyset)$ ▷ Init backbone tree with the root of MST
$S := \emptyset$ ▷ Set of vertices close enough to the backbone
while $|V(B)| + |S| < (1 - \rho)|V(G)|$ **do**
    $V_s := T.\mathit{neighbours}(V(B))$ ▷ Next branch need to start near current backbone
    $R := T.\mathit{induced\_subgraph}(V(T) \setminus V(B))$ ▷ Create (disconnected) subgraph by removing backbone vertices
    $P := \max_{v_1 \in V_s \cap V(R), v_2 \in V(R)} R.\mathit{shortest\_path}(v_1, v_2)$ ▷ Longest shortest path between starting set and non-backbone vertices
    $B.\mathit{add\_path}(P)$ ▷ Add branch to backbone
    $S := \{v \in V(G) \setminus V(B) \mid \exists v_b \in V(B), d(v_b, v) < \delta\}$
end while
for $u := B.\mathit{backbone}_D \mathit{FS}()$ **do** ▷ Depth-first-search through all backbone vertices
    for $v := u.\mathit{backbone}_c \mathit{hildren}()$ **do** ▷ Remove close contiguous backbone nodes
        if $d(u, v) < \delta$ **then**
            if $|\{w \in V(G) \setminus V(B) \mid \exists v_b \in V(B) \setminus v, d(v_b, w) < \delta\}| < \rho|V(G)|$ **then**
                $\mathit{merge\_backbone}_v \mathit{ertices}(u, v)$ ▷ remove v from backbone
            else
                if $|\{w \in V(G) \setminus V(B) \mid \exists v_b \in V(B) \setminus u, d(v_b, w) < \delta\}| < \rho|V(G)|$ **then**
                    $\mathit{merge\_backbone}_v \mathit{ertices}(v, u)$ ▷ remove u from backbone
                end if
            end if
        end if
    end for
end for
Output: B
