# Supplementary material for: CellTree: an R/bioconductor package to infer the hierarchical structure of cell populations from single-cell RNA-seq data
Source: BMC Bioinformatics. 2016 Sep 13;17(1):363. doi: 10.1186/s12859-016-1175-6 (PMC5020541; doi:10.1186/s12859-016-1175-6)

Ordered cells by branch

Legend: Topic #1 Topic #2 Topic #3 Topic #4 Topic #5

Table 1: Branch 1

node.label	cell.name	cell.group	main.topic	topics
4	T0_CT_A06	0	1	
40	T0_CT_E08	0	1	
13	T0_CT_B08	0	1	
15	T0_CT_C02	0	1	
30	T0_CT_D08	0	1	
60	T0_CT_G08	0	1	
59	T0_CT_G07	0	1	
10	T0_CT_B03	0	1	
58	T0_CT_G04	0	1	
28	T0_CT_D06	0	1	
41	T0_CT_E09	0	1	
34	T0_CT_E01	0	1	
65	T0_CT_H04	0	1	
1	T0_CT_A01	0	1	
53	T0_CT_F11	0	1	
196	T48_CT_E12	48	1	
126	T24_CT_F12	24	1	
150	T48_CT_A07	48	1	
24	T0_CT_D01	0	5	
45	T0_CT_F01	0	1	

Table 2: Branch 1.1

node.label	cell.name	cell.group	main.topic	topics
2	T0_CT_A03	0	1	
50	T0_CT_F06	0	1	
26	T0_CT_D03	0	1	
20	T0_CT_C08	0	1	
23	T0_CT_C12	0	1	
55	T0_CT_G01	0	1	
5	T0_CT_A07	0	1	
3	T0_CT_A05	0	1	
268	T72_CT_H09	72	1	
47	T0_CT_F03	0	1	
137	T24_CT_H01	24	1	
14	T0_CT_B09	0	1	
39	T0_CT_E07	0	1	
38	T0_CT_E06	0	1	
57	T0_CT_G03	0	1	
43	T0_CT_E11	0	1	
54	T0_CT_F12	0	1	

46	T0_CT_F02	0	1
33	T0_CT_D12	0	1
48	T0_CT_F04	0	1
44	T0_CT_E12	0	1
86	T24_CT_B11	24	1
211	T48_CT_G10	48	1
73	T24_CT_A05	24	1
7	T0_CT_A10	0	1
35	T0_CT_E03	0	1
19	T0_CT_C07	0	1
25	T0_CT_D02	0	1
69	T0_CT_H12	0	1
66	T0_CT_H05	0	1
27	T0_CT_D05	0	2
9	T0_CT_B01	0	2
68	T0_CT_H09	0	2
63	T0_CT_H01	0	2
112	T24_CT_E07	24	2
130	T24_CT_G04	24	5
18	T0_CT_C06	0	2
148	T48_CT_A05	48	5
188	T48_CT_E03	48	2
124	T24_CT_F10	24	2
62	T0_CT_G11	0	1
261	T72_CT_G08	72	4
51	T0_CT_F07	0	5
67	T0_CT_H08	0	1
12	T0_CT_B07	0	1
11	T0_CT_B05	0	1
29	T0_CT_D07	0	1
56	T0_CT_G02	0	1
16	T0_CT_C03	0	3
70	T24_CT_A01	24	3
205	T48_CT_G01	48	3
136	T24_CT_G12	24	3
113	T24_CT_E09	24	3
133	T24_CT_G08	24	3
87	T24_CT_C01	24	3
142	T24_CT_H09	24	3
85	T24_CT_B09	24	3
76	T24_CT_A09	24	3
143	T24_CT_H12	24	3
138	T24_CT_H02	24	3
89	T24_CT_C03	24	3
101	T24_CT_D05	24	3
103	T24_CT_D07	24	2
95	T24_CT_C11	24	2
78	T24_CT_B01	24	3
102	T24_CT_D06	24	3
219	T48_CT_H07	48	2

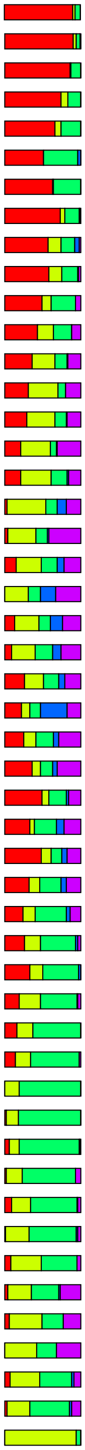

140	T24_CT_H05	24	3
121	T24_CT_F07	24	2
119	T24_CT_F04	24	2
37	T0_CT_E05	0	2
77	T24_CT_A10	24	2
203	T48_CT_F10	48	2
212	T48_CT_G11	48	2
132	T24_CT_G06	24	3
109	T24_CT_E02	24	3
122	T24_CT_F08	24	3
79	T24_CT_B02	24	3
94	T24_CT_C10	24	3
100	T24_CT_D04	24	3
177	T48_CT_D03	48	3
194	T48_CT_E10	48	3
141	T24_CT_H07	24	3
248	T72_CT_D10	72	4
199	T48_CT_F03	48	3
61	T0_CT_G09	0	3
269	T72_CT_H10	72	4
198	T48_CT_F02	48	2
164	T48_CT_B12	48	2
183	T48_CT_D10	48	2
189	T48_CT_E04	48	2
201	T48_CT_F07	48	3
118	T24_CT_F03	24	3
120	T24_CT_F05	24	3
152	T48_CT_A09	48	3
171	T48_CT_C07	48	2
125	T24_CT_F11	24	2
176	T48_CT_D02	48	2
108	T24_CT_E01	24	3
155	T48_CT_A12	48	2
187	T48_CT_E02	48	2
221	T48_CT_H11	48	2
181	T48_CT_D08	48	3
114	T24_CT_E11	24	3
195	T48_CT_E11	48	3
161	T48_CT_B08	48	3
173	T48_CT_C10	48	3
204	T48_CT_F11	48	3
225	T72_CT_A08	72	3
270	T72_CT_H11	72	4
236	T72_CT_B11	72	4
250	T72_CT_E04	72	4
255	T72_CT_F07	72	4
239	T72_CT_C06	72	4
230	T72_CT_B03	72	4
263	T72_CT_G11	72	4
241	T72_CT_C09	72	4

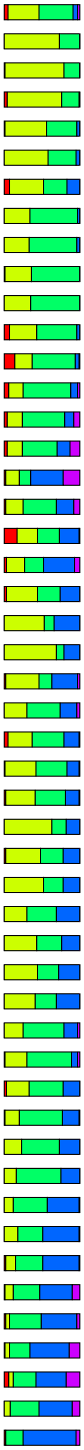

244	T72_CT_D03	72	3	
247	T72_CT_D07	72	4	
246	T72_CT_D05	72	4	
233	T72_CT_B06	72	3	
229	T72_CT_B02	72	3	
249	T72_CT_D11	72	3	
231	T72_CT_B04	72	3	
259	T72_CT_G04	72	3	
192	T48_CT_E07	48	3	
258	T72_CT_G03	72	3	
6	T0_CT_A08	0	3	
75	T24_CT_A08	24	3	
146	T48_CT_A03	48	3	
235	T72_CT_B09	72	3	
159	T48_CT_B04	48	3	
128	T24_CT_G02	24	3	
185	T48_CT_D12	48	3	
191	T48_CT_E06	48	3	
135	T24_CT_G11	24	3	
111	T24_CT_E05	24	3	
216	T48_CT_H04	48	3	
166	T48_CT_C02	48	3	
149	T48_CT_A06	48	3	
129	T24_CT_G03	24	3	
83	T24_CT_B07	24	3	
168	T48_CT_C04	48	3	
158	T48_CT_B03	48	3	
8	T0_CT_A11	0	3	

Table 3: Branch 1.2

node.label	cell.name	cell.group	main.topic	topics
49	T0_CT_F05	0	5	
64	T0_CT_H02	0	5	
21	T0_CT_C09	0	5	
31	T0_CT_D09	0	5	
36	T0_CT_E04	0	5	
17	T0_CT_C05	0	5	
32	T0_CT_D11	0	5	
123	T24_CT_F09	24	5	
72	T24_CT_A04	24	5	
98	T24_CT_D02	24	5	
52	T0_CT_F09	0	5	
117	T24_CT_F02	24	5	
106	T24_CT_D10	24	5	
127	T24_CT_G01	24	5	
90	T24_CT_C05	24	5	
99	T24_CT_D03	24	5	
22	T0_CT_C11	0	5	
104	T24_CT_D08	24	5	

84	T24_CT_B08	24	5
92	T24_CT_C08	24	5
156	T48_CT_B01	48	5
93	T24_CT_C09	24	5
218	T48_CT_H06	48	5
74	T24_CT_A07	24	5
134	T24_CT_G10	24	5
131	T24_CT_G05	24	5
213	T48_CT_G12	48	5
88	T24_CT_C02	24	5
153	T48_CT_A10	48	5
157	T48_CT_B02	48	2
97	T24_CT_D01	24	2
182	T48_CT_D09	48	2
197	T48_CT_F01	48	5
175	T48_CT_D01	48	2
214	T48_CT_H01	48	5
139	T24_CT_H03	24	5
217	T48_CT_H05	48	5
208	T48_CT_G07	48	5
81	T24_CT_B05	24	5
82	T24_CT_B06	24	5
116	T24_CT_F01	24	2
207	T48_CT_G03	48	5
115	T24_CT_E12	24	2
145	T48_CT_A02	48	5
107	T24_CT_D11	24	5
160	T48_CT_B06	48	5
202	T48_CT_F09	48	5
71	T24_CT_A03	24	5
147	T48_CT_A04	48	5
162	T48_CT_B10	48	5
167	T48_CT_C03	48	2
110	T24_CT_E04	24	5
91	T24_CT_C07	24	2
190	T48_CT_E05	48	2
96	T24_CT_C12	24	5
80	T24_CT_B03	24	2
154	T48_CT_A11	48	5
209	T48_CT_G08	48	2
200	T48_CT_F05	48	5
193	T48_CT_E08	48	5
174	T48_CT_C11	48	5
180	T48_CT_D07	48	5
170	T48_CT_C06	48	2
165	T48_CT_C01	48	5
210	T48_CT_G09	48	2
220	T48_CT_H08	48	5
169	T48_CT_C05	48	5
151	T48_CT_A08	48	5

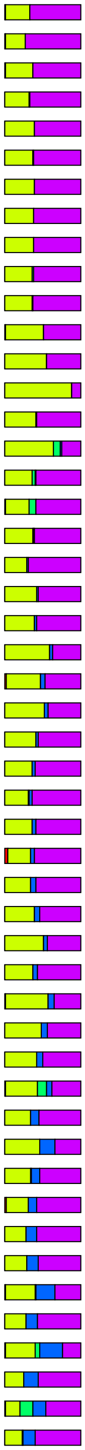

206	T48_CT_G02	48	5
163	T48_CT_B11	48	5
105	T24_CT_D09	24	5
222	T48_CT_H12	48	5
178	T48_CT_D04	48	5
179	T48_CT_D06	48	5
42	T0_CT_E10	0	5
144	T48_CT_A01	48	5
228	T72_CT_B01	72	5
224	T72_CT_A05	72	5
223	T72_CT_A01	72	5
215	T48_CT_H02	48	5
227	T72_CT_A11	72	5
232	T72_CT_B05	72	4
237	T72_CT_B12	72	5
256	T72_CT_F10	72	5
243	T72_CT_D01	72	5
265	T72_CT_H03	72	5
253	T72_CT_F01	72	5
262	T72_CT_G10	72	5
271	T72_CT_H12	72	5
186	T48_CT_E01	48	4
226	T72_CT_A09	72	5
251	T72_CT_E05	72	5
254	T72_CT_F05	72	5
264	T72_CT_H01	72	5
238	T72_CT_C04	72	5
252	T72_CT_E07	72	5
245	T72_CT_D04	72	5
240	T72_CT_C07	72	5
172	T48_CT_C09	48	5
184	T48_CT_D11	48	5
257	T72_CT_F11	72	5
260	T72_CT_G06	72	5
234	T72_CT_B08	72	5
266	T72_CT_H05	72	1
267	T72_CT_H08	72	1
242	T72_CT_C11	72	1

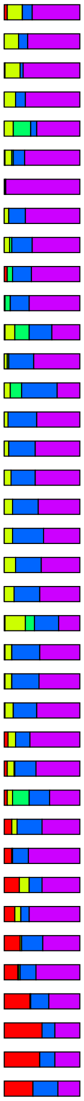

Supplement: Additional file 2 — Gene probability distribution for topic 3. Per-topic gene probability distribution for Topic 3 of the hESC data model computed by cellTree. (PDF 56 kb) [file 12859_2016_1175_MOESM2_ESM.pdf]
