# Supplementary material for: CellTree: an R/bioconductor package to infer the hierarchical structure of cell populations from single-cell RNA-seq data
Source: BMC Bioinformatics. 2016 Sep 13;17(1):363. doi: 10.1186/s12859-016-1175-6 (PMC5020541; doi:10.1186/s12859-016-1175-6)
Supplement: Additional file 3 — GO BP Terms for myoblast data. Full table of enriched GO BP terms for each topic in myoblast data. (PDF 36 kb) [file 12859_2016_1175_MOESM3_ESM.pdf]

	GO.ID	Term	Total	p-Value
1	GO:0070124	mitochondrial translational initiation	82	1.8e-21
2	GO:0070125	mitochondrial translational elongation	82	1.8e-21
3	GO:0000398	mRNA splicing, via spliceosome	250	3.2e-21
4	GO:0070126	mitochondrial translational termination	84	2.1e-19
5	GO:0051436	negative regulation of ubiquitin-protein ligase activity involved in mitotic cell cycle	82	7.5e-17
6	GO:0051437	positive regulation of ubiquitin-protein ligase activity involved in regulation of mitotic cell cycle transition	64	1.2e-16
7	GO:0006521	regulation of cellular amino acid metabolic process	52	1.3e-16
8	GO:0031145	anaphase-promoting complex-dependent proteasomal ubiquitin-dependent protein catabolic process	106	1.3e-15
9	GO:0002479	antigen processing and presentation of exogenous peptide antigen via MHC class I, TAP-dependent	59	2.7e-15
10	GO:0006977	DNA damage response, signal transduction by p53 class mediator resulting in cell cycle arrest	57	3.2e-15
11	GO:0006120	mitochondrial electron transport, NADH to ubiquinone	40	1.6e-14
12	GO:0006614	SRP-dependent cotranslational protein targeting to membrane	32	3.1e-14
13	GO:0038061	NIK/NF-kappaB signaling	76	1.6e-13
14	GO:0016032	viral process	556	1.9e-12
15	GO:0006412	translation	423	2.0e-12
16	GO:0051301	cell division	512	4.7e-12
17	GO:0007067	mitotic nuclear division	359	1.4e-10
18	GO:0019083	viral transcription	100	1.7e-10
19	GO:0043066	negative regulation of apoptotic process	533	2.5e-10
20	GO:0006283	transcription-coupled nucleotide-excision repair	43	5.7e-10
21	GO:0006446	regulation of translational initiation	66	9.3e-10
22	GO:0007264	small GTPase mediated signal transduction	624	1.7e-09
23	GO:0006406	mRNA export from nucleus	68	2.1e-09
24	GO:0033209	tumor necrosis factor-mediated signaling pathway	95	2.1e-09
25	GO:0022904	respiratory electron transport chain	104	3.4e-09
26	GO:0090263	positive regulation of canonical Wnt signaling pathway	89	5.4e-09
27	GO:0006413	translational initiation	173	6.9e-09
28	GO:0007077	mitotic nuclear envelope disassembly	40	7.8e-09
29	GO:0008380	RNA splicing	331	1.0e-08
30	GO:0016925	protein sumoylation	95	1.1e-08
31	GO:0002223	stimulatory C-type lectin receptor signaling pathway	95	1.4e-08
32	GO:0006368	transcription elongation from RNA polymerase II promoter	79	2.1e-08
33	GO:0000086	G2/M transition of mitotic cell cycle	161	2.7e-08
34	GO:0007059	chromosome segregation	219	4.5e-08
35	GO:0010827	regulation of glucose transport	74	4.6e-08
36	GO:0000082	G1/S transition of mitotic cell cycle	208	6.0e-08
37	GO:0090090	negative regulation of canonical Wnt signaling pathway	103	6.1e-08
38	GO:0006369	termination of RNA polymerase II transcription	45	1.1e-07
39	GO:0042769	DNA damage response, detection of DNA damage	31	1.3e-07
40	GO:0000381	regulation of alternative mRNA splicing, via spliceosome	20	1.3e-07
41	GO:1900034	regulation of cellular response to heat	63	1.4e-07
42	GO:0042776	mitochondrial ATP synthesis coupled proton transport	14	6.4e-07
43	GO:0007265	Ras protein signal transduction	367	6.9e-07
44	GO:0010467	gene expression	3621	7.2e-07
45	GO:0010499	proteasomal ubiquitin-independent protein catabolic process	20	1.5e-06
46	GO:0006271	DNA strand elongation involved in DNA replication	35	1.6e-06
47	GO:0006626	protein targeting to mitochondrion	118	1.7e-06

Table 1: Topic 1 (All terms)

	GO.ID	Term	Total	p-Value
1	GO:0000398	mRNA splicing, via spliceosome	250	6.7e-14
2	GO:0016032	viral process	556	1.6e-12
3	GO:0002479	antigen processing and presentation of exogenous peptide antigen via MHC class I, TAP-dependent	59	3.6e-12
4	GO:0006120	mitochondrial electron transport, NADH to ubiquinone	40	7.2e-12
5	GO:0006614	SRP-dependent cotranslational protein targeting to membrane	32	6.9e-11
6	GO:0070124	mitochondrial translational initiation	82	2.5e-10
7	GO:0043066	negative regulation of apoptotic process	533	9.9e-10
8	GO:0051436	negative regulation of ubiquitin-protein ligase activity involved in mitotic cell cycle	82	1.3e-09
9	GO:0070125	mitochondrial translational elongation	82	2.1e-09
10	GO:0006977	DNA damage response, signal transduction by p53 class mediator resulting in cell cycle arrest	57	4.2e-09
11	GO:0006413	translational initiation	173	5.0e-09
12	GO:0070126	mitochondrial translational termination	84	6.7e-09
13	GO:0090090	negative regulation of canonical Wnt signaling pathway	103	1.2e-08
14	GO:0006521	regulation of cellular amino acid metabolic process	52	1.3e-08
15	GO:0007411	axon guidance	336	1.8e-08
16	GO:0006446	regulation of translational initiation	66	2.9e-08
17	GO:0006367	transcription initiation from RNA polymerase II promoter	204	9.9e-08
18	GO:0000381	regulation of alternative mRNA splicing, via spliceosome	20	2.2e-07
19	GO:0048010	vascular endothelial growth factor receptor signaling pathway	193	3.6e-07
20	GO:0006368	transcription elongation from RNA polymerase II promoter	79	4.0e-07
21	GO:0038061	NIK/NF-kappaB signaling	76	4.4e-07
22	GO:0022904	respiratory electron transport chain	104	8.8e-07
23	GO:0010499	proteasomal ubiquitin-independent protein catabolic process	20	1.1e-06
24	GO:0006376	mRNA splice site selection	24	1.1e-06
25	GO:0031145	anaphase-promoting complex-dependent proteasomal ubiquitin-dependent protein catabolic process	106	1.3e-06
26	GO:0008286	insulin receptor signaling pathway	230	1.5e-06
27	GO:0010467	gene expression	3621	1.7e-06

Table 2: Topic 2 (All terms)

	GO.ID	Term	Total	p-Value
1	GO:0070125	mitochondrial translational elongation	82	2.0e-21
2	GO:0070124	mitochondrial translational initiation	82	1.7e-20
3	GO:0070126	mitochondrial translational termination	84	2.0e-19
4	GO:0006120	mitochondrial electron transport, NADH to ubiquinone	40	5.8e-18
5	GO:0006521	regulation of cellular amino acid metabolic process	52	5.8e-18
6	GO:0051436	negative regulation of ubiquitin-protein ligase activity involved in mitotic cell cycle	82	1.4e-17
7	GO:0002479	antigen processing and presentation of exogenous peptide antigen via MHC class I, TAP-dependent	59	2.7e-17
8	GO:0000398	mRNA splicing, via spliceosome	250	1.1e-16
9	GO:0006977	DNA damage response, signal transduction by p53 class mediator resulting in cell cycle arrest	57	1.4e-15
10	GO:0031145	anaphase-promoting complex-dependent proteasomal ubiquitin-dependent protein catabolic process	106	1.1e-14
11	GO:0038061	NIK/NF-kappaB signaling	76	2.4e-14
12	GO:0006614	SRP-dependent cotranslational protein targeting to membrane	32	3.3e-14
13	GO:0051437	positive regulation of ubiquitin-protein ligase activity involved in regulation of mitotic cell cycle transition	64	6.6e-13
14	GO:0016032	viral process	556	2.2e-12
15	GO:0006446	regulation of translational initiation	66	5.3e-11
16	GO:0007265	Ras protein signal transduction	367	1.0e-10
17	GO:0022904	respiratory electron transport chain	104	4.0e-10
18	GO:0090263	positive regulation of canonical Wnt signaling pathway	89	5.8e-10
19	GO:0033209	tumor necrosis factor-mediated signaling pathway	95	1.1e-09
20	GO:0006412	translation	423	4.0e-09
21	GO:0043066	negative regulation of apoptotic process	533	4.6e-09
22	GO:0002223	stimulatory C-type lectin receptor signaling pathway	95	4.9e-09
23	GO:0010467	gene expression	3621	6.7e-09
24	GO:0030049	muscle filament sliding	29	2.5e-08
25	GO:0042776	mitochondrial ATP synthesis coupled proton transport	14	2.6e-08
26	GO:0090090	negative regulation of canonical Wnt signaling pathway	103	3.1e-08
27	GO:0006413	translational initiation	173	6.9e-08
28	GO:0006368	transcription elongation from RNA polymerase II promoter	79	8.9e-08
29	GO:0007411	axon guidance	336	1.1e-07
30	GO:0008286	insulin receptor signaling pathway	230	1.1e-07
31	GO:0048010	vascular endothelial growth factor receptor signaling pathway	193	1.9e-07
32	GO:0051084	'de novo' posttranslational protein folding	41	3.6e-07
33	GO:0000381	regulation of alternative mRNA splicing, via spliceosome	20	4.0e-07
34	GO:0010499	proteasomal ubiquitin-independent protein catabolic process	20	5.5e-07
35	GO:0017148	negative regulation of translation	76	1.1e-06
36	GO:0006406	mRNA export from nucleus	68	1.4e-06
37	GO:0001731	formation of translation preinitiation complex	16	1.7e-06
38	GO:0000186	activation of MAPKK activity	154	1.8e-06

Table 3: Topic 3 (All terms)

	GO.ID	Term	Total	p-Value
1	GO:0007411	axon guidance	336	5.8e-12
2	GO:0006614	SRP-dependent cotranslational protein targeting to membrane	32	2.2e-10
3	GO:0016032	viral process	556	2.0e-09
4	GO:0006977	DNA damage response, signal transduction by p53 class mediator resulting in cell cycle arrest	57	6.8e-09
5	GO:0002479	antigen processing and presentation of exogenous peptide antigen via MHC class I, TAP-dependent	59	1.1e-08
6	GO:0043066	negative regulation of apoptotic process	533	1.1e-07
7	GO:0006521	regulation of cellular amino acid metabolic process	52	5.1e-07
8	GO:0000398	mRNA splicing, via spliceosome	250	6.4e-07
9	GO:0006120	mitochondrial electron transport, NADH to ubiquinone	40	6.8e-07
10	GO:0006886	intracellular protein transport	742	9.6e-07
11	GO:0007265	Ras protein signal transduction	367	1.4e-06

Table 4: Topic 4 (All terms)

	GO.ID	Term	Total	p-Value
1	GO:0070125	mitochondrial translational elongation	82	1.0e-26
2	GO:0070124	mitochondrial translational initiation	82	1.1e-25
3	GO:0070126	mitochondrial translational termination	84	1.8e-23
4	GO:0006120	mitochondrial electron transport, NADH to ubiquinone	40	2.0e-19
5	GO:0002479	antigen processing and presentation of exogenous peptide antigen via MHC class I, TAP-dependent	59	7.5e-19
6	GO:0006521	regulation of cellular amino acid metabolic process	52	1.3e-18
7	GO:0006977	DNA damage response, signal transduction by p53 class mediator resulting in cell cycle arrest	57	7.0e-18
8	GO:0051436	negative regulation of ubiquitin-protein ligase activity involved in mitotic cell cycle	82	7.7e-18
9	GO:0006614	SRP-dependent cotranslational protein targeting to membrane	32	1.3e-17
10	GO:0000398	mRNA splicing, via spliceosome	250	3.9e-15
11	GO:0038061	NIK/NF-kappaB signaling	76	1.8e-14
12	GO:0043066	negative regulation of apoptotic process	533	6.0e-14
13	GO:0016032	viral process	556	7.5e-14
14	GO:0031145	anaphase-promoting complex-dependent proteasomal ubiquitin-dependent protein catabolic process	106	9.4e-14
15	GO:0051437	positive regulation of ubiquitin-protein ligase activity involved in regulation of mitotic cell cycle transition	64	7.5e-13
16	GO:0090263	positive regulation of canonical Wnt signaling pathway	89	2.5e-11
17	GO:0022904	respiratory electron transport chain	104	7.9e-11
18	GO:0006446	regulation of translational initiation	66	2.6e-10
19	GO:0033209	tumor necrosis factor-mediated signaling pathway	95	8.3e-10
20	GO:0007265	Ras protein signal transduction	367	1.4e-09
21	GO:0006368	transcription elongation from RNA polymerase II promoter	79	2.4e-09
22	GO:0010467	gene expression	3621	3.2e-09
23	GO:0002223	stimulatory C-type lectin receptor signaling pathway	95	3.5e-09
24	GO:0090090	negative regulation of canonical Wnt signaling pathway	103	2.5e-08
25	GO:0006412	translation	423	3.3e-08
26	GO:0042776	mitochondrial ATP synthesis coupled proton transport	14	7.2e-08
27	GO:0006413	translational initiation	173	2.0e-07
28	GO:0010499	proteasomal ubiquitin-independent protein catabolic process	20	3.0e-07
29	GO:0050434	positive regulation of viral transcription	49	3.0e-07
30	GO:0051084	'de novo' posttranslational protein folding	41	3.3e-07
31	GO:0006370	7-methylguanosine mRNA capping	30	4.3e-07
32	GO:0044267	cellular protein metabolic process	3243	4.7e-07
33	GO:0001731	formation of translation preinitiation complex	16	7.0e-07
34	GO:0007411	axon guidance	336	1.5e-06
35	GO:0006406	mRNA export from nucleus	68	1.6e-06
36	GO:0006457	protein folding	184	1.6e-06

Table 5: Topic 5 (All terms)

	GO.ID	Term	Total	p-Value
6	GO:0051437	positive regulation of ubiquitin-protein ligase activity involved in regulation of mitotic cell cycle transition	64	1.2e-16
15	GO:0006412	translation	423	2.0e-12
16	GO:0051301	cell division	512	4.7e-12
17	GO:0007067	mitotic nuclear division	359	1.4e-10
18	GO:0019083	viral transcription	100	1.7e-10
20	GO:0006283	transcription-coupled nucleotide-excision repair	43	5.7e-10
22	GO:0007264	small GTPase mediated signal transduction	624	1.7e-09
23	GO:0006406	mRNA export from nucleus	68	2.1e-09
24	GO:0033209	tumor necrosis factor-mediated signaling pathway	95	2.1e-09
26	GO:0090263	positive regulation of canonical Wnt signaling pathway	89	5.4e-09
28	GO:0007077	mitotic nuclear envelope disassembly	40	7.8e-09
29	GO:0008380	RNA splicing	331	1.0e-08
30	GO:0016925	protein sumoylation	95	1.1e-08
31	GO:0002223	stimulatory C-type lectin receptor signaling pathway	95	1.4e-08
33	GO:0000086	G2/M transition of mitotic cell cycle	161	2.7e-08
34	GO:0007059	chromosome segregation	219	4.5e-08
35	GO:0010827	regulation of glucose transport	74	4.6e-08
36	GO:0000082	G1/S transition of mitotic cell cycle	208	6.0e-08
38	GO:0006369	termination of RNA polymerase II transcription	45	1.1e-07
39	GO:0042769	DNA damage response, detection of DNA damage	31	1.3e-07
40	GO:0000381	regulation of alternative mRNA splicing, via spliceosome	20	1.3e-07
41	GO:1900034	regulation of cellular response to heat	63	1.4e-07
42	GO:0042776	mitochondrial ATP synthesis coupled proton transport	14	6.4e-07
46	GO:0006271	DNA strand elongation involved in DNA replication	35	1.6e-06
47	GO:0006626	protein targeting to mitochondrion	118	1.7e-06

Table 6: Topic 1 (Terms that appear in less than half of other topics)

	GO.ID	Term	Total	p-Value
17	GO:0006367	transcription initiation from RNA polymerase II promoter	204	9.9e-08
18	GO:0000381	regulation of alternative mRNA splicing, via spliceosome	20	2.2e-07
19	GO:0048010	vascular endothelial growth factor receptor signaling pathway	193	3.6e-07
24	GO:0006376	mRNA splice site selection	24	1.1e-06
26	GO:0008286	insulin receptor signaling pathway	230	1.5e-06

Table 7: Topic 2 (Terms that appear in less than half of other topics)

	GO.ID	Term	Total	p-Value
13	GO:0051437	positive regulation of ubiquitin-protein ligase activity involved in regulation of mitotic cell cycle transition	64	6.6e-13
18	GO:0090263	positive regulation of canonical Wnt signaling pathway	89	5.8e-10
19	GO:0033209	tumor necrosis factor-mediated signaling pathway	95	1.1e-09
20	GO:0006412	translation	423	4.0e-09
22	GO:0002223	stimulatory C-type lectin receptor signaling pathway	95	4.9e-09
24	GO:0030049	muscle filament sliding	29	2.5e-08
25	GO:0042776	mitochondrial ATP synthesis coupled proton transport	14	2.6e-08
30	GO:0008286	insulin receptor signaling pathway	230	1.1e-07
31	GO:0048010	vascular endothelial growth factor receptor signaling pathway	193	1.9e-07
32	GO:0051084	'de novo' posttranslational protein folding	41	3.6e-07
33	GO:0000381	regulation of alternative mRNA splicing, via spliceosome	20	4.0e-07
35	GO:0017148	negative regulation of translation	76	1.1e-06
36	GO:0006406	mRNA export from nucleus	68	1.4e-06
37	GO:0001731	formation of translation preinitiation complex	16	1.7e-06
38	GO:0000186	activation of MAPKK activity	154	1.8e-06

Table 8: Topic 3 (Terms that appear in less than half of other topics)

	GO.ID	Term	Total	p-Value
10	GO:0006886	intracellular protein transport	742	9.6e-07

Table 9: Topic 4 (Terms that appear in less than half of other topics)

	GO.ID	Term	Total	p-Value
15	GO:0051437	positive regulation of ubiquitin-protein ligase activity involved in regulation of mitotic cell cycle transition	64	7.5e-13
16	GO:0090263	positive regulation of canonical Wnt signaling pathway	89	2.5e-11
19	GO:0033209	tumor necrosis factor-mediated signaling pathway	95	8.3e-10
23	GO:0002223	stimulatory C-type lectin receptor signaling pathway	95	3.5e-09
25	GO:0006412	translation	423	3.3e-08
26	GO:0042776	mitochondrial ATP synthesis coupled proton transport	14	7.2e-08
29	GO:0050434	positive regulation of viral transcription	49	3.0e-07
30	GO:0051084	'de novo' posttranslational protein folding	41	3.3e-07
31	GO:0006370	7-methylguanosine mRNA capping	30	4.3e-07
32	GO:0044267	cellular protein metabolic process	3243	4.7e-07
33	GO:0001731	formation of translation preinitiation complex	16	7.0e-07
35	GO:0006406	mRNA export from nucleus	68	1.6e-06
36	GO:0006457	protein folding	184	1.6e-06

Table 10: Topic 5 (Terms that appear in less than half of other topics)

	GO.ID	Term	Total	p-Value
16	GO:0051301	cell division	512	4.7e-12
17	GO:0007067	mitotic nuclear division	359	1.4e-10
18	GO:0019083	viral transcription	100	1.7e-10
20	GO:0006283	transcription-coupled nucleotide-excision repair	43	5.7e-10
22	GO:0007264	small GTPase mediated signal transduction	624	1.7e-09
28	GO:0007077	mitotic nuclear envelope disassembly	40	7.8e-09
29	GO:0008380	RNA splicing	331	1.0e-08
30	GO:0016925	protein sumoylation	95	1.1e-08
33	GO:0000086	G2/M transition of mitotic cell cycle	161	2.7e-08
34	GO:0007059	chromosome segregation	219	4.5e-08
35	GO:0010827	regulation of glucose transport	74	4.6e-08
36	GO:0000082	G1/S transition of mitotic cell cycle	208	6.0e-08
38	GO:0006369	termination of RNA polymerase II transcription	45	1.1e-07
39	GO:0042769	DNA damage response, detection of DNA damage	31	1.3e-07
41	GO:1900034	regulation of cellular response to heat	63	1.4e-07
46	GO:0006271	DNA strand elongation involved in DNA replication	35	1.6e-06
47	GO:0006626	protein targeting to mitochondrion	118	1.7e-06

Table 11: Topic 1 (Terms that only appear in this topic)

	GO.ID	Term	Total	p-Value
17	GO:0006367	transcription initiation from RNA polymerase II promoter	204	9.9e-08
24	GO:0006376	mRNA splice site selection	24	1.1e-06

Table 12: Topic 2 (Terms that only appear in this topic)

	GO.ID	Term	Total	p-Value
24	GO:0030049	muscle filament sliding	29	2.5e-08
35	GO:0017148	negative regulation of translation	76	1.1e-06
38	GO:0000186	activation of MAPKK activity	154	1.8e-06

Table 13: Topic 3 (Terms that only appear in this topic)

	GO.ID	Term	Total	p-Value
10	GO:0006886	intracellular protein transport	742	9.6e-07

Table 14: Topic 4 (Terms that only appear in this topic)

	GO.ID	Term	Total	p-Value
29	GO:0050434	positive regulation of viral transcription	49	3.0e-07
31	GO:0006370	7-methylguanosine mRNA capping	30	4.3e-07
32	GO:0044267	cellular protein metabolic process	3243	4.7e-07
36	GO:0006457	protein folding	184	1.6e-06

Table 15: Topic 5 (Terms that only appear in this topic)
