# Supplementary material for: CellTree: an R/bioconductor package to infer the hierarchical structure of cell populations from single-cell RNA-seq data
Source: BMC Bioinformatics. 2016 Sep 13;17(1):363. doi: 10.1186/s12859-016-1175-6 (PMC5020541; doi:10.1186/s12859-016-1175-6)
Supplement: Additional file 4 — GO CC Terms for myoblast data. Full table of enriched GO CC terms for each topic in myoblast data. (PDF 38 kb) [file 12859_2016_1175_MOESM4_ESM.pdf]

	GO.ID	Term	Total	p-Value
1	GO:0070062	extracellular exosome	1736	< 1e-30
2	GO:0005654	nucleoplasm	2397	< 1e-30
3	GO:0005829	cytosol	2245	7.3e-24
4	GO:0005730	nucleolus	696	1.8e-22
5	GO:0016020	membrane	4667	6.1e-20
6	GO:0005747	mitochondrial respiratory chain complex I	40	7.3e-17
7	GO:0005737	cytoplasm	6973	2.9e-16
8	GO:0071013	catalytic step 2 spliceosome	81	3.4e-16
9	GO:0043209	myelin sheath	122	9.9e-14
10	GO:0043234	protein complex	2881	8.3e-13
11	GO:0005743	mitochondrial inner membrane	393	1.5e-11
12	GO:0000777	condensed chromosome kinetochore	89	1.1e-10
13	GO:0016607	nuclear speck	171	1.3e-10
14	GO:0005763	mitochondrial small ribosomal subunit	24	4.1e-10
15	GO:0005925	focal adhesion	302	3.0e-09
16	GO:0030529	ribonucleoprotein complex	542	4.4e-08
17	GO:0005840	ribosome	126	5.3e-08
18	GO:0005681	spliceosomal complex	162	6.2e-08
19	GO:0005839	proteasome core complex	17	7.0e-08
20	GO:0005643	nuclear pore	61	8.9e-08
21	GO:0010494	cytoplasmic stress granule	28	1.6e-07
22	GO:0005753	mitochondrial proton-transporting ATP synthase complex	17	2.2e-07
23	GO:0005852	eukaryotic translation initiation factor 3 complex	15	4.6e-07
24	GO:0008540	proteasome regulatory particle, base subcomplex	12	5.5e-07
25	GO:0042470	melanosome	79	1.0e-06
26	GO:0005813	centrosome	400	1.4e-06
27	GO:0033290	eukaryotic 48S preinitiation complex	14	1.8e-06
28	GO:0000922	spindle pole	105	2.2e-06
29	GO:0005832	chaperonin-containing T-complex	9	3.3e-06
30	GO:0005876	spindle microtubule	49	3.4e-06
31	GO:0071011	precatalytic spliceosome	21	3.6e-06
32	GO:0005762	mitochondrial large ribosomal subunit	32	4.6e-06
33	GO:0005689	U12-type spliceosomal complex	25	4.8e-06
34	GO:0005688	U6 snRNP	8	5.7e-06
35	GO:0005686	U2 snRNP	16	7.8e-06
36	GO:0000940	condensed chromosome outer kinetochore	13	9.5e-06
37	GO:0005759	mitochondrial matrix	324	1.1e-05
38	GO:0031597	cytosolic proteasome complex	11	1.2e-05
39	GO:0005758	mitochondrial intermembrane space	63	1.2e-05
40	GO:0000784	nuclear chromosome, telomeric region	63	1.2e-05
41	GO:0046540	U4/U6 x U5 tri-snRNP complex	16	1.3e-05

Table 1: Topic 1 (All terms)

	GO.ID	Term	Total	p-Value
1	GO:0070062	extracellular exosome	1736	< 1e-30
2	GO:0005925	focal adhesion	302	6.1e-15
3	GO:0005747	mitochondrial respiratory chain complex I	40	2.7e-13
4	GO:0005829	cytosol	2245	4.3e-11
5	GO:0016020	membrane	4667	1.6e-10
6	GO:0071013	catalytic step 2 spliceosome	81	3.3e-10
7	GO:0043209	myelin sheath	122	7.7e-10
8	GO:0005654	nucleoplasm	2397	1.6e-08
9	GO:0016607	nuclear speck	171	4.6e-08
10	GO:0010494	cytoplasmic stress granule	28	2.7e-07
11	GO:0005839	proteasome core complex	17	2.9e-07
12	GO:0005753	mitochondrial proton-transporting ATP synthase complex	17	5.2e-07
13	GO:0042470	melanosome	79	1.2e-06
14	GO:0005852	eukaryotic translation initiation factor 3 complex	15	1.6e-06
15	GO:0005665	DNA-directed RNA polymerase II, core complex	15	5.1e-06
16	GO:0033290	eukaryotic 48S preinitiation complex	14	6.7e-06
17	GO:0005763	mitochondrial small ribosomal subunit	24	1.2e-05
18	GO:0005737	cytoplasm	6973	1.3e-05

Table 2: Topic 2 (All terms)

	GO.ID	Term	Total	p-Value
1	GO:0070062	extracellular exosome	1736	$< 1e-30$
2	GO:0005747	mitochondrial respiratory chain complex I	40	1.5e-20
3	GO:0005829	cytosol	2245	6.8e-20
4	GO:0071013	catalytic step 2 spliceosome	81	1.4e-16
5	GO:0043209	myelin sheath	122	6.4e-16
6	GO:0016020	membrane	4667	7.6e-14
7	GO:0005925	focal adhesion	302	2.4e-13
8	GO:0005730	nucleolus	696	5.7e-12
9	GO:0005737	cytoplasm	6973	1.9e-11
10	GO:0005743	mitochondrial inner membrane	393	2.9e-11
11	GO:0005763	mitochondrial small ribosomal subunit	24	1.6e-09
12	GO:0016607	nuclear speck	171	4.6e-09
13	GO:0030529	ribonucleoprotein complex	542	6.4e-09
14	GO:0042470	melanosome	79	1.0e-08
15	GO:0010494	cytoplasmic stress granule	28	1.2e-08
16	GO:0005839	proteasome core complex	17	2.7e-08
17	GO:0005753	mitochondrial proton-transporting ATP synthase complex	17	6.3e-08
18	GO:0001725	stress fiber	37	1.7e-07
19	GO:0005654	nucleoplasm	2397	2.1e-07
20	GO:0008540	proteasome regulatory particle, base subcomplex	12	3.1e-07
21	GO:0030018	Z disc	77	3.1e-07
22	GO:0005852	eukaryotic translation initiation factor 3 complex	15	4.0e-07
23	GO:0005746	mitochondrial respiratory chain	62	8.4e-07
24	GO:0033290	eukaryotic 48S preinitiation complex	14	8.8e-07
25	GO:0098796	membrane protein complex	634	1.2e-06
26	GO:0098800	inner mitochondrial membrane protein complex	91	1.3e-06
27	GO:0043234	protein complex	2881	1.3e-06
28	GO:0005762	mitochondrial large ribosomal subunit	32	1.5e-06
29	GO:0071011	precatalytic spliceosome	21	2.4e-06
30	GO:0000932	cytoplasmic mRNA processing body	59	2.9e-06
31	GO:0005793	endoplasmic reticulum-Golgi intermediate compartment	66	3.1e-06
32	GO:0005832	chaperonin-containing T-complex	9	3.8e-06
33	GO:0031597	cytosolic proteasome complex	11	4.3e-06
34	GO:0005840	ribosome	126	6.9e-06
35	GO:0005758	mitochondrial intermembrane space	63	8.6e-06
36	GO:0005686	U2 snRNP	16	9.1e-06
37	GO:0005681	spliceosomal complex	162	1.2e-05
38	GO:0016282	eukaryotic 43S preinitiation complex	14	1.3e-05

Table 3: Topic 3 (All terms)

	GO.ID	Term	Total	p-Value
1	GO:0070062	extracellular exosome	1736	< 1e-30
2	GO:0005925	focal adhesion	302	8.9e-13
3	GO:0005829	cytosol	2245	3.1e-10
4	GO:0016020	membrane	4667	1.3e-08
5	GO:0005747	mitochondrial respiratory chain complex I	40	2.0e-08
6	GO:0005654	nucleoplasm	2397	2.2e-08
7	GO:0005737	cytoplasm	6973	3.0e-08
8	GO:0016607	nuclear speck	171	4.8e-08
9	GO:0071013	catalytic step 2 spliceosome	81	5.6e-07
10	GO:0010494	cytoplasmic stress granule	28	1.8e-06
11	GO:0043209	myelin sheath	122	2.2e-06
12	GO:0005852	eukaryotic translation initiation factor 3 complex	15	6.0e-06
13	GO:0005604	basement membrane	58	1.0e-05

Table 4: Topic 4 (All terms)

	GO.ID	Term	Total	p-Value
1	GO:0070062	extracellular exosome	1736	< 1e-30
2	GO:0005829	cytosol	2245	3.8e-22
3	GO:0005747	mitochondrial respiratory chain complex I	40	4.0e-22
4	GO:0043209	myelin sheath	122	2.7e-19
5	GO:0005743	mitochondrial inner membrane	393	4.0e-17
6	GO:0016020	membrane	4667	9.9e-16
7	GO:0005925	focal adhesion	302	2.8e-13
8	GO:0071013	catalytic step 2 spliceosome	81	2.0e-12
9	GO:0005730	nucleolus	696	1.2e-11
10	GO:0005840	ribosome	126	7.9e-10
11	GO:0005737	cytoplasm	6973	1.1e-09
12	GO:0005763	mitochondrial small ribosomal subunit	24	1.3e-09
13	GO:0005839	proteasome core complex	17	9.0e-09
14	GO:0042470	melanosome	79	1.4e-08
15	GO:0005654	nucleoplasm	2397	6.1e-08
16	GO:0005762	mitochondrial large ribosomal subunit	32	7.5e-08
17	GO:0016607	nuclear speck	171	8.6e-08
18	GO:0005753	mitochondrial proton-transporting ATP synthase complex	17	1.4e-07
19	GO:0005643	nuclear pore	61	1.5e-07
20	GO:0005852	eukaryotic translation initiation factor 3 complex	15	1.9e-07
21	GO:0030529	ribonucleoprotein complex	542	2.8e-07
22	GO:0033290	eukaryotic 48S preinitiation complex	14	7.0e-07
23	GO:0008540	proteasome regulatory particle, base subcomplex	12	7.0e-07
24	GO:0043234	protein complex	2881	9.6e-07
25	GO:0010494	cytoplasmic stress granule	28	2.0e-06
26	GO:0005789	endoplasmic reticulum membrane	628	2.4e-06
27	GO:0098796	membrane protein complex	634	5.3e-06
28	GO:0005746	mitochondrial respiratory chain	62	5.3e-06
29	GO:0005793	endoplasmic reticulum-Golgi intermediate compartment	66	7.5e-06
30	GO:0031597	cytosolic proteasome complex	11	8.6e-06
31	GO:0005832	chaperonin-containing T-complex	9	8.7e-06
32	GO:0005758	mitochondrial intermembrane space	63	9.4e-06
33	GO:0016282	eukaryotic 43S preinitiation complex	14	1.1e-05
34	GO:0005885	Arp2/3 protein complex	10	1.3e-05

Table 5: Topic 5 (All terms)

	GO.ID	Term	Total	p-Value
4	GO:0005730	nucleolus	696	1.8e-22
10	GO:0043234	protein complex	2881	8.3e-13
11	GO:0005743	mitochondrial inner membrane	393	1.5e-11
12	GO:0000777	condensed chromosome kinetochore	89	1.1e-10
16	GO:0030529	ribonucleoprotein complex	542	4.4e-08
17	GO:0005840	ribosome	126	5.3e-08
18	GO:0005681	spliceosomal complex	162	6.2e-08
20	GO:0005643	nuclear pore	61	8.9e-08
24	GO:0008540	proteasome regulatory particle, base subcomplex	12	5.5e-07
26	GO:0005813	centrosome	400	1.4e-06
28	GO:0000922	spindle pole	105	2.2e-06
29	GO:0005832	chaperonin-containing T-complex	9	3.3e-06
30	GO:0005876	spindle microtubule	49	3.4e-06
31	GO:0071011	precatalytic spliceosome	21	3.6e-06
32	GO:0005762	mitochondrial large ribosomal subunit	32	4.6e-06
33	GO:0005689	U12-type spliceosomal complex	25	4.8e-06
34	GO:0005688	U6 snRNP	8	5.7e-06
35	GO:0005686	U2 snRNP	16	7.8e-06
36	GO:0000940	condensed chromosome outer kinetochore	13	9.5e-06
37	GO:0005759	mitochondrial matrix	324	1.1e-05
38	GO:0031597	cytosolic proteasome complex	11	1.2e-05
39	GO:0005758	mitochondrial intermembrane space	63	1.2e-05
40	GO:0000784	nuclear chromosome, telomeric region	63	1.2e-05
41	GO:0046540	U4/U6 x U5 tri-snRNP complex	16	1.3e-05

Table 6: Topic 1 (Terms that appear in less than half of other topics)

	GO.ID	Term	Total	p-Value
15	GO:0005665	DNA-directed RNA polymerase II, core complex	15	5.1e-06

Table 7: Topic 2 (Terms that appear in less than half of other topics)

	GO.ID	Term	Total	p-Value
8	GO:0005730	nucleolus	696	5.7e-12
10	GO:0005743	mitochondrial inner membrane	393	2.9e-11
13	GO:0030529	ribonucleoprotein complex	542	6.4e-09
18	GO:0001725	stress fiber	37	1.7e-07
20	GO:0008540	proteasome regulatory particle, base subcomplex	12	3.1e-07
21	GO:0030018	Z disc	77	3.1e-07
23	GO:0005746	mitochondrial respiratory chain	62	8.4e-07
25	GO:0098796	membrane protein complex	634	1.2e-06
26	GO:0098800	inner mitochondrial membrane protein complex	91	1.3e-06
27	GO:0043234	protein complex	2881	1.3e-06
28	GO:0005762	mitochondrial large ribosomal subunit	32	1.5e-06
29	GO:0071011	precatalytic spliceosome	21	2.4e-06
30	GO:0000932	cytoplasmic mRNA processing body	59	2.9e-06
31	GO:0005793	endoplasmic reticulum-Golgi intermediate compartment	66	3.1e-06
32	GO:0005832	chaperonin-containing T-complex	9	3.8e-06
33	GO:0031597	cytosolic proteasome complex	11	4.3e-06
34	GO:0005840	ribosome	126	6.9e-06
35	GO:0005758	mitochondrial intermembrane space	63	8.6e-06
36	GO:0005686	U2 snRNP	16	9.1e-06
37	GO:0005681	spliceosomal complex	162	1.2e-05
38	GO:0016282	eukaryotic 43S preinitiation complex	14	1.3e-05

Table 8: Topic 3 (Terms that appear in less than half of other topics)

	GO.ID	Term	Total	p-Value
13	GO:0005604	basement membrane	58	1.0e-05

Table 9: Topic 4 (Terms that appear in less than half of other topics)

	GO.ID	Term	Total	p-Value
5	GO:0005743	mitochondrial inner membrane	393	4.0e-17
9	GO:0005730	nucleolus	696	1.2e-11
10	GO:0005840	ribosome	126	7.9e-10
16	GO:0005762	mitochondrial large ribosomal subunit	32	7.5e-08
19	GO:0005643	nuclear pore	61	1.5e-07
21	GO:0030529	ribonucleoprotein complex	542	2.8e-07
23	GO:0008540	proteasome regulatory particle, base subcomplex	12	7.0e-07
24	GO:0043234	protein complex	2881	9.6e-07
26	GO:0005789	endoplasmic reticulum membrane	628	2.4e-06
27	GO:0098796	membrane protein complex	634	5.3e-06
28	GO:0005746	mitochondrial respiratory chain	62	5.3e-06
29	GO:0005793	endoplasmic reticulum-Golgi intermediate compartment	66	7.5e-06
30	GO:0031597	cytosolic proteasome complex	11	8.6e-06
31	GO:0005832	chaperonin-containing T-complex	9	8.7e-06
32	GO:0005758	mitochondrial intermembrane space	63	9.4e-06
33	GO:0016282	eukaryotic 43S preinitiation complex	14	1.1e-05
34	GO:0005885	Arp2/3 protein complex	10	1.3e-05

Table 10: Topic 5 (Terms that appear in less than half of other topics)

	GO.ID	Term	Total	p-Value
12	GO:0000777	condensed chromosome kinetochore	89	1.1e-10
26	GO:0005813	centrosome	400	1.4e-06
28	GO:0000922	spindle pole	105	2.2e-06
30	GO:0005876	spindle microtubule	49	3.4e-06
33	GO:0005689	U12-type spliceosomal complex	25	4.8e-06
34	GO:0005688	U6 snRNP	8	5.7e-06
36	GO:0000940	condensed chromosome outer kinetochore	13	9.5e-06
37	GO:0005759	mitochondrial matrix	324	1.1e-05
40	GO:0000784	nuclear chromosome, telomeric region	63	1.2e-05
41	GO:0046540	U4/U6 x U5 tri-snRNP complex	16	1.3e-05

Table 11: Topic 1 (Terms that only appear in this topic)

	GO.ID	Term	Total	p-Value
15	GO:0005665	DNA-directed RNA polymerase II, core complex	15	5.1e-06

Table 12: Topic 2 (Terms that only appear in this topic)

	GO.ID	Term	Total	p-Value
18	GO:0001725	stress fiber	37	1.7e-07
21	GO:0030018	Z disc	77	3.1e-07
26	GO:0098800	inner mitochondrial membrane protein complex	91	1.3e-06
30	GO:0000932	cytoplasmic mRNA processing body	59	2.9e-06

Table 13: Topic 3 (Terms that only appear in this topic)

	GO.ID	Term	Total	p-Value
13	GO:0005604	basement membrane	58	1.0e-05

Table 14: Topic 4 (Terms that only appear in this topic)

	GO.ID	Term	Total	p-Value
26	GO:0005789	endoplasmic reticulum membrane	628	2.4e-06
34	GO:0005885	Arp2/3 protein complex	10	1.3e-05

Table 15: Topic 5 (Terms that only appear in this topic)
