# Supplementary material for: CellTree: an R/bioconductor package to infer the hierarchical structure of cell populations from single-cell RNA-seq data
Source: BMC Bioinformatics. 2016 Sep 13;17(1):363. doi: 10.1186/s12859-016-1175-6 (PMC5020541; doi:10.1186/s12859-016-1175-6)
Supplement: Additional file 5 — cellTree summary for hESC data. Full list of cell samples in the hESC data set, ordered and annotated by cellTree. (PDF 36 kb) [file 12859_2016_1175_MOESM5_ESM.pdf]

Ordered cells by branch

Legend: Topic #1 Topic #2 Topic #3 Topic #4

Table 1: Branch 1

node.label	cell.name	cell.group	main.topic	topics
2	Oocyte.2	oocyte	3	
3	Oocyte.3	oocyte	3	
1	Oocyte.1	oocyte	3	
4	Zygote.1	zygote	3	
6	Zygote.3	zygote	3	
5	Zygote.2	zygote	3	
15	X4.cell.embryo.1.Cell.3	X4.cell.embryo	3	
7	X2.cell.embryo.1.Cell.1	X2.cell.embryo	3	
10	X2.cell.embryo.2.Cell.2	X2.cell.embryo	3	
8	X2.cell.embryo.1.Cell.2	X2.cell.embryo	3	
12	X2.cell.embryo.3.Cell.2	X2.cell.embryo	3	
11	X2.cell.embryo.3.Cell.1	X2.cell.embryo	3	
20	X4.cell.embryo.2.Cell.4	X4.cell.embryo	3	
9	X2.cell.embryo.2.Cell.1	X2.cell.embryo	3	
18	X4.cell.embryo.2.Cell.2	X4.cell.embryo	3	
16	X4.cell.embryo.1.Cell.4	X4.cell.embryo	3	
14	X4.cell.embryo.1.Cell.2	X4.cell.embryo	3	
22	X4.cell.embryo.3.Cell.2	X4.cell.embryo	3	
13	X4.cell.embryo.1.Cell.1	X4.cell.embryo	3	
24	X4.cell.embryo.3.Cell.4	X4.cell.embryo	3	
23	X4.cell.embryo.3.Cell.3	X4.cell.embryo	3	
21	X4.cell.embryo.3.Cell.1	X4.cell.embryo	3	
17	X4.cell.embryo.2.Cell.1	X4.cell.embryo	3	
19	X4.cell.embryo.2.Cell.3	X4.cell.embryo	3	
25	X8.cell.embryo.1.Cell.1	X8.cell.embryo	3	
27	X8.cell.embryo.1.Cell.3	X8.cell.embryo	3	
28	X8.cell.embryo.1.Cell.4	X8.cell.embryo	3	
26	X8.cell.embryo.1.Cell.2	X8.cell.embryo	3	
47	Morulae.1.Cell.3	Morulae.1	4	
52	Morulae.1.Cell.8	Morulae.1	4	
40	X8.cell.embryo.3.Cell.4	X8.cell.embryo	4	
37	X8.cell.embryo.3.Cell.1	X8.cell.embryo	4	
41	X8.cell.embryo.3.Cell.5	X8.cell.embryo	4	
38	X8.cell.embryo.3.Cell.2	X8.cell.embryo	4	
43	X8.cell.embryo.3.Cell.7	X8.cell.embryo	4	
42	X8.cell.embryo.3.Cell.6	X8.cell.embryo	4	
39	X8.cell.embryo.3.Cell.3	X8.cell.embryo	4	
30	X8.cell.embryo.2.Cell.2	X8.cell.embryo	4	
31	X8.cell.embryo.2.Cell.3	X8.cell.embryo	4	
36	X8.cell.embryo.2.Cell.8	X8.cell.embryo	4	
29	X8.cell.embryo.2.Cell.1	X8.cell.embryo	4	
34	X8.cell.embryo.2.Cell.6	X8.cell.embryo	4	

44	X8.cell.embryo.3.Cell.8	X8.cell.embryo	4	
35	X8.cell.embryo.2.Cell.7	X8.cell.embryo	4	
33	X8.cell.embryo.2.Cell.5	X8.cell.embryo	4	
32	X8.cell.embryo.2.Cell.4	X8.cell.embryo	4	
56	Morulae.2.Cell.4	Morulae.2	4	
58	Morulae.2.Cell.6	Morulae.2	4	
55	Morulae.2.Cell.3	Morulae.2	4	
50	Morulae.1.Cell.6	Morulae.1	4	
53	Morulae.2.Cell.1	Morulae.2	4	
57	Morulae.2.Cell.5	Morulae.2	4	
60	Morulae.2.Cell.8	Morulae.2	4	
46	Morulae.1.Cell.2	Morulae.1	4	
54	Morulae.2.Cell.2	Morulae.2	4	
51	Morulae.1.Cell.7	Morulae.1	4	
48	Morulae.1.Cell.4	Morulae.1	4	
59	Morulae.2.Cell.7	Morulae.2	4	
45	Morulae.1.Cell.1	Morulae.1	4	
49	Morulae.1.Cell.5	Morulae.1	4	
74	Late.blastocyst.2.Cell.2	Late.blastocyst.2	2	
75	Late.blastocyst.2.Cell.3	Late.blastocyst.2	2	
83	Late.blastocyst.3.Cell.1	Late.blastocyst.3	2	
86	Late.blastocyst.3.Cell.4	Late.blastocyst.3	2	
73	Late.blastocyst.2.Cell.1	Late.blastocyst.2	2	

Table 2: Branch 1.1

node.label	cell.name	cell.group	main.topic	topics
84	Late.blastocyst.3.Cell.2	Late.blastocyst.3	2	
80	Late.blastocyst.2.Cell.8	Late.blastocyst.2	2	
90	Late.blastocyst.3.Cell.8	Late.blastocyst.3	2	
69	Late.blastocyst.1.Cell.9	Late.blastocyst.1	2	
89	Late.blastocyst.3.Cell.7	Late.blastocyst.3	2	
72	Late.blastocyst.1.Cell.12	Late.blastocyst.1	2	
77	Late.blastocyst.2.Cell.5	Late.blastocyst.2	2	
78	Late.blastocyst.2.Cell.6	Late.blastocyst.2	2	
79	Late.blastocyst.2.Cell.7	Late.blastocyst.2	2	
81	Late.blastocyst.2.Cell.9	Late.blastocyst.2	2	
64	Late.blastocyst.1.Cell.4	Late.blastocyst.1	2	
82	Late.blastocyst.2.Cell.10	Late.blastocyst.2	2	
66	Late.blastocyst.1.Cell.6	Late.blastocyst.1	2	
85	Late.blastocyst.3.Cell.3	Late.blastocyst.3	2	
88	Late.blastocyst.3.Cell.6	Late.blastocyst.3	2	
65	Late.blastocyst.1.Cell.5	Late.blastocyst.1	2	
76	Late.blastocyst.2.Cell.4	Late.blastocyst.2	2	
68	Late.blastocyst.1.Cell.8	Late.blastocyst.1	2	
67	Late.blastocyst.1.Cell.7	Late.blastocyst.1	2	
70	Late.blastocyst.1.Cell.10	Late.blastocyst.1	2	
61	Late.blastocyst.1.Cell.1	Late.blastocyst.1	2	
62	Late.blastocyst.1.Cell.2	Late.blastocyst.1	2	
63	Late.blastocyst.1.Cell.3	Late.blastocyst.1	2	

71	Late.blastocyst.1.Cell.11	Late.blastocyst.1	2	

Table 3: Branch 1.2

node.label	cell.name	cell.group	main.topic	topics
87	Late.blastocyst.3.Cell.5	Late.blastocyst.3	2	
96	hESC.passage.0.Cell.6	hESC.passage.0	1	
92	hESC.passage.0.Cell.2	hESC.passage.0	1	
95	hESC.passage.0.Cell.5	hESC.passage.0	1	
98	hESC.passage.0.Cell.8	hESC.passage.0	1	
118	hESC.passage.10.Cell.20	hESC.passage.10	1	
94	hESC.passage.0.Cell.4	hESC.passage.0	1	
119	hESC.passage.10.Cell.21	hESC.passage.10	1	
97	hESC.passage.0.Cell.7	hESC.passage.0	1	
91	hESC.passage.0.Cell.1	hESC.passage.0	1	
113	hESC.passage.10.Cell.15	hESC.passage.10	1	
117	hESC.passage.10.Cell.19	hESC.passage.10	1	
115	hESC.passage.10.Cell.17	hESC.passage.10	1	
114	hESC.passage.10.Cell.16	hESC.passage.10	1	
106	hESC.passage.10.Cell.8	hESC.passage.10	1	
102	hESC.passage.10.Cell.4	hESC.passage.10	1	
112	hESC.passage.10.Cell.14	hESC.passage.10	1	
110	hESC.passage.10.Cell.12	hESC.passage.10	1	
116	hESC.passage.10.Cell.18	hESC.passage.10	1	
100	hESC.passage.10.Cell.2	hESC.passage.10	1	
104	hESC.passage.10.Cell.6	hESC.passage.10	1	
121	hESC.passage.10.Cell.23	hESC.passage.10	1	
93	hESC.passage.0.Cell.3	hESC.passage.0	1	
111	hESC.passage.10.Cell.13	hESC.passage.10	1	
101	hESC.passage.10.Cell.3	hESC.passage.10	1	
103	hESC.passage.10.Cell.5	hESC.passage.10	1	
99	hESC.passage.10.Cell.1	hESC.passage.10	1	
123	hESC.passage.10.Cell.25	hESC.passage.10	1	
108	hESC.passage.10.Cell.10	hESC.passage.10	1	
122	hESC.passage.10.Cell.24	hESC.passage.10	1	
105	hESC.passage.10.Cell.7	hESC.passage.10	1	
120	hESC.passage.10.Cell.22	hESC.passage.10	1	
109	hESC.passage.10.Cell.11	hESC.passage.10	1	
107	hESC.passage.10.Cell.9	hESC.passage.10	1	
