# Supplementary material for: CellTree: an R/bioconductor package to infer the hierarchical structure of cell populations from single-cell RNA-seq data
Source: BMC Bioinformatics. 2016 Sep 13;17(1):363. doi: 10.1186/s12859-016-1175-6 (PMC5020541; doi:10.1186/s12859-016-1175-6)
Supplement: Additional file 6 — GO BP Terms for hESC data. Full table of enriched GO BP terms for each topic in hESC data. (PDF 42 kb) [file 12859_2016_1175_MOESM6_ESM.pdf]

	GO.ID	Term	Total	p-Value
1	GO:0070124	mitochondrial translational initiation	77	2.2e-28
2	GO:0070125	mitochondrial translational elongation	77	7.9e-28
3	GO:0070126	mitochondrial translational termination	79	9.3e-26
4	GO:0006614	SRP-dependent cotranslational protein targeting to membrane	54	3.9e-25
5	GO:0006415	translational termination	121	2.1e-23
6	GO:0006413	translational initiation	194	5.7e-22
7	GO:0000184	nuclear-transcribed mRNA catabolic process, nonsense-mediated decay	64	7.8e-20
8	GO:0006414	translational elongation	138	4.1e-19
9	GO:0002479	antigen processing and presentation of exogenous peptide antigen via MHC class I, TAP-dependent	51	2.8e-16
10	GO:0031145	anaphase-promoting complex-dependent proteasomal ubiquitin-dependent protein catabolic process	92	1.1e-15
11	GO:0000398	mRNA splicing, via spliceosome	200	1.9e-15
12	GO:0019083	viral transcription	118	2.2e-15
13	GO:0051437	positive regulation of ubiquitin-protein ligase activity involved in regulation of mitotic cell cycle transition	57	1.3e-14
14	GO:0051436	negative regulation of ubiquitin-protein ligase activity involved in mitotic cell cycle	71	1.8e-14
15	GO:0006521	regulation of cellular amino acid metabolic process	48	4.1e-13
16	GO:0006977	DNA damage response, signal transduction by p53 class mediator resulting in cell cycle arrest	49	2.6e-12
17	GO:0022904	respiratory electron transport chain	107	9.7e-12
18	GO:0006120	mitochondrial electron transport, NADH to ubiquinone	40	1.0e-11
19	GO:0038061	NIK/NF-kappaB signaling	71	2.8e-11
20	GO:0016032	viral process	525	3.2e-11
21	GO:0006446	regulation of translational initiation	62	8.7e-10
22	GO:0042776	mitochondrial ATP synthesis coupled proton transport	17	1.9e-09
23	GO:0006418	tRNA aminoacylation for protein translation	43	2.3e-09
24	GO:0006406	mRNA export from nucleus	62	2.8e-09
25	GO:0006271	DNA strand elongation involved in DNA replication	35	7.0e-09
26	GO:0042769	DNA damage response, detection of DNA damage	28	3.5e-08
27	GO:0033209	tumor necrosis factor-mediated signaling pathway	87	4.0e-08
28	GO:0006626	protein targeting to mitochondrion	100	6.4e-08
29	GO:0090263	positive regulation of canonical Wnt signaling pathway	86	3.0e-07
30	GO:0045814	negative regulation of gene expression, epigenetic	127	4.1e-07
31	GO:1902600	hydrogen ion transmembrane transport	70	4.2e-07
32	GO:0001731	formation of translation preinitiation complex	14	6.0e-07
33	GO:0006283	transcription-coupled nucleotide-excision repair	43	6.0e-07
34	GO:0051084	'de novo' posttranslational protein folding	35	1.3e-06
35	GO:0010467	gene expression	3415	1.5e-06

Table 1: Topic 1 (All terms)

	GO.ID	Term	Total	p-Value
1	GO:0070124	mitochondrial translational initiation	77	$< 1e-30$
2	GO:0070125	mitochondrial translational elongation	77	$< 1e-30$
3	GO:0070126	mitochondrial translational termination	79	$< 1e-30$
4	GO:0006614	SRP-dependent cotranslational protein targeting to membrane	54	3.1e-29
5	GO:0006415	translational termination	121	2.6e-25
6	GO:0006413	translational initiation	194	2.7e-23
7	GO:0006414	translational elongation	138	1.4e-20
8	GO:0000184	nuclear-transcribed mRNA catabolic process, nonsense-mediated decay	64	1.5e-20
9	GO:0006120	mitochondrial electron transport, NADH to ubiquinone	40	3.5e-18
10	GO:0019083	viral transcription	118	1.4e-17
11	GO:0000398	mRNA splicing, via spliceosome	200	4.3e-15
12	GO:0031145	anaphase-promoting complex-dependent proteasomal ubiquitin-dependent protein catabolic process	92	1.5e-14
13	GO:0051437	positive regulation of ubiquitin-protein ligase activity involved in regulation of mitotic cell cycle transition	57	2.4e-14
14	GO:0051436	negative regulation of ubiquitin-protein ligase activity involved in mitotic cell cycle	71	7.2e-13
15	GO:0006521	regulation of cellular amino acid metabolic process	48	1.9e-12
16	GO:0022904	respiratory electron transport chain	107	3.3e-12
17	GO:0006446	regulation of translational initiation	62	5.9e-11
18	GO:0006364	rRNA processing	124	7.1e-11
19	GO:0006977	DNA damage response, signal transduction by p53 class mediator resulting in cell cycle arrest	49	7.6e-11
20	GO:0002479	antigen processing and presentation of exogenous peptide antigen via MHC class I, TAP-dependent	51	1.3e-10
21	GO:0038061	NIK/NF-kappaB signaling	71	3.9e-10
22	GO:0006626	protein targeting to mitochondrion	100	5.1e-10
23	GO:0016032	viral process	525	2.5e-09
24	GO:0042776	mitochondrial ATP synthesis coupled proton transport	17	7.7e-09
25	GO:0001731	formation of translation preinitiation complex	14	3.8e-08
26	GO:0006406	mRNA export from nucleus	62	5.6e-08
27	GO:1900034	regulation of cellular response to heat	57	7.0e-08
28	GO:0051301	cell division	464	1.3e-07
29	GO:0044743	intracellular protein transmembrane import	29	2.1e-07
30	GO:0006271	DNA strand elongation involved in DNA replication	35	2.6e-07
31	GO:0000462	maturation of SSU-rRNA from tricistronic rRNA transcript (SSU-rRNA, 5.8S rRNA, LSU-rRNA)	21	3.3e-07
32	GO:0033209	tumor necrosis factor-mediated signaling pathway	87	4.4e-07
33	GO:0006418	tRNA aminoacylation for protein translation	43	4.6e-07
34	GO:0006369	termination of RNA polymerase II transcription	39	5.5e-07
35	GO:0007077	mitotic nuclear envelope disassembly	34	7.2e-07
36	GO:1990542	mitochondrial transmembrane transport	37	9.7e-07
37	GO:0031124	mRNA 3'-end processing	68	1.3e-06
38	GO:0006283	transcription-coupled nucleotide-excision repair	43	1.6e-06
39	GO:1902600	hydrogen ion transmembrane transport	70	1.6e-06

Table 2: Topic 2 (All terms)

	GO.ID	Term	Total	p-Value
1	GO:0006415	translational termination	121	2.1e-18
2	GO:0006413	translational initiation	194	1.7e-17
3	GO:0006614	SRP-dependent cotranslational protein targeting to membrane	54	5.2e-17
4	GO:0006414	translational elongation	138	1.8e-16
5	GO:0019083	viral transcription	118	5.4e-16
6	GO:0000184	nuclear-transcribed mRNA catabolic process, nonsense-mediated decay	64	1.9e-15
7	GO:0031145	anaphase-promoting complex-dependent proteasomal ubiquitin-dependent protein catabolic process	92	2.2e-09
8	GO:0051437	positive regulation of ubiquitin-protein ligase activity involved in regulation of mitotic cell cycle transition	57	1.4e-08
9	GO:0000281	mitotic cytokinesis	25	1.3e-07
10	GO:0038061	NIK/NF-kappaB signaling	71	8.1e-07
11	GO:0051436	negative regulation of ubiquitin-protein ligase activity involved in mitotic cell cycle	71	8.5e-07

Table 3: Topic 3 (All terms)

	GO.ID	Term	Total	p-Value
1	GO:0006413	translational initiation	194	7.4e-22
2	GO:0006614	SRP-dependent cotranslational protein targeting to membrane	54	1.5e-21
3	GO:0006415	translational termination	121	3.0e-21
4	GO:0000184	nuclear-transcribed mRNA catabolic process, nonsense-mediated decay	64	5.6e-21
5	GO:0000398	mRNA splicing, via spliceosome	200	5.8e-19
6	GO:0006414	translational elongation	138	1.1e-17
7	GO:0019083	viral transcription	118	3.1e-17
8	GO:0070125	mitochondrial translational elongation	77	3.2e-16
9	GO:0006364	rRNA processing	124	7.0e-16
10	GO:0070124	mitochondrial translational initiation	77	2.1e-15
11	GO:0070126	mitochondrial translational termination	79	1.6e-14
12	GO:0051437	positive regulation of ubiquitin-protein ligase activity involved in regulation of mitotic cell cycle transition	57	7.5e-12
13	GO:0031145	anaphase-promoting complex-dependent proteasomal ubiquitin-dependent protein catabolic process	92	1.1e-11
14	GO:0051436	negative regulation of ubiquitin-protein ligase activity involved in mitotic cell cycle	71	5.2e-10
15	GO:0010501	RNA secondary structure unwinding	33	1.5e-09
16	GO:0006521	regulation of cellular amino acid metabolic process	48	4.3e-09
17	GO:0000462	maturation of SSU-rRNA from tricistronic rRNA transcript (SSU-rRNA, 5.8S rRNA, LSU-rRNA)	21	4.6e-09
18	GO:0045596	negative regulation of cell differentiation	309	9.5e-09
19	GO:0002479	antigen processing and presentation of exogenous peptide antigen via MHC class I, TAP-dependent	51	1.0e-08
20	GO:0038061	NIK/NF-kappaB signaling	71	1.1e-08
21	GO:0006406	mRNA export from nucleus	62	1.8e-08
22	GO:0048387	negative regulation of retinoic acid receptor signaling pathway	25	1.9e-08
23	GO:0006446	regulation of translational initiation	62	2.1e-08
24	GO:0031124	mRNA 3'-end processing	68	2.2e-08
25	GO:0006369	termination of RNA polymerase II transcription	39	3.5e-08
26	GO:0016032	viral process	525	8.1e-08
27	GO:0001731	formation of translation preinitiation complex	14	1.1e-07
28	GO:0043066	negative regulation of apoptotic process	516	1.4e-07
29	GO:0006368	transcription elongation from RNA polymerase II promoter	72	2.0e-07
30	GO:0006977	DNA damage response, signal transduction by p53 class mediator resulting in cell cycle arrest	49	2.7e-07
31	GO:0010467	gene expression	3415	3.5e-07

Table 4: Topic 4 (All terms)

	GO.ID	Term	Total	p-Value
1	GO:0070124	mitochondrial translational initiation	77	2.2e-28
2	GO:0070125	mitochondrial translational elongation	77	7.9e-28
3	GO:0070126	mitochondrial translational termination	79	9.3e-26
9	GO:0002479	antigen processing and presentation of exogenous peptide antigen via MHC class I, TAP-dependent	51	2.8e-16
11	GO:0000398	mRNA splicing, via spliceosome	200	1.9e-15
15	GO:0006521	regulation of cellular amino acid metabolic process	48	4.1e-13
16	GO:0006977	DNA damage response, signal transduction by p53 class mediator resulting in cell cycle arrest	49	2.6e-12
17	GO:0022904	respiratory electron transport chain	107	9.7e-12
18	GO:0006120	mitochondrial electron transport, NADH to ubiquinone	40	1.0e-11
20	GO:0016032	viral process	525	3.2e-11
21	GO:0006446	regulation of translational initiation	62	8.7e-10
22	GO:0042776	mitochondrial ATP synthesis coupled proton transport	17	1.9e-09
23	GO:0006418	tRNA aminoacylation for protein translation	43	2.3e-09
24	GO:0006406	mRNA export from nucleus	62	2.8e-09
25	GO:0006271	DNA strand elongation involved in DNA replication	35	7.0e-09
26	GO:0042769	DNA damage response, detection of DNA damage	28	3.5e-08
27	GO:0033209	tumor necrosis factor-mediated signaling pathway	87	4.0e-08
28	GO:0006626	protein targeting to mitochondrion	100	6.4e-08
29	GO:0090263	positive regulation of canonical Wnt signaling pathway	86	3.0e-07
30	GO:0045814	negative regulation of gene expression, epigenetic	127	4.1e-07
31	GO:1902600	hydrogen ion transmembrane transport	70	4.2e-07
32	GO:0001731	formation of translation preinitiation complex	14	6.0e-07
33	GO:0006283	transcription-coupled nucleotide-excision repair	43	6.0e-07
34	GO:0051084	'de novo' posttranslational protein folding	35	1.3e-06
35	GO:0010467	gene expression	3415	1.5e-06

Table 5: Topic 1 (Terms that appear in less than half of other topics)

	GO.ID	Term	Total	p-Value
1	GO:0070124	mitochondrial translational initiation	77	$<$ 1e-30
2	GO:0070125	mitochondrial translational elongation	77	$<$ 1e-30
3	GO:0070126	mitochondrial translational termination	79	$<$ 1e-30
9	GO:0006120	mitochondrial electron transport, NADH to ubiquinone	40	3.5e-18
11	GO:0000398	mRNA splicing, via spliceosome	200	4.3e-15
15	GO:0006521	regulation of cellular amino acid metabolic process	48	1.9e-12
16	GO:0022904	respiratory electron transport chain	107	3.3e-12
17	GO:0006446	regulation of translational initiation	62	5.9e-11
18	GO:0006364	rRNA processing	124	7.1e-11
19	GO:0006977	DNA damage response, signal transduction by p53 class mediator resulting in cell cycle arrest	49	7.6e-11
20	GO:0002479	antigen processing and presentation of exogenous peptide antigen via MHC class I, TAP-dependent	51	1.3e-10
22	GO:0006626	protein targeting to mitochondrion	100	5.1e-10
23	GO:0016032	viral process	525	2.5e-09
24	GO:0042776	mitochondrial ATP synthesis coupled proton transport	17	7.7e-09
25	GO:0001731	formation of translation preinitiation complex	14	3.8e-08
26	GO:0006406	mRNA export from nucleus	62	5.6e-08
27	GO:1900034	regulation of cellular response to heat	57	7.0e-08
28	GO:0051301	cell division	464	1.3e-07
29	GO:0044743	intracellular protein transmembrane import	29	2.1e-07
30	GO:0006271	DNA strand elongation involved in DNA replication	35	2.6e-07
31	GO:0000462	maturation of SSU-rRNA from tricistronic rRNA transcript (SSU-rRNA, 5.8S rRNA, LSU-rRNA)	21	3.3e-07
32	GO:0033209	tumor necrosis factor-mediated signaling pathway	87	4.4e-07
33	GO:0006418	tRNA aminoacylation for protein translation	43	4.6e-07
34	GO:0006369	termination of RNA polymerase II transcription	39	5.5e-07
35	GO:0007077	mitotic nuclear envelope disassembly	34	7.2e-07
36	GO:1990542	mitochondrial transmembrane transport	37	9.7e-07
37	GO:0031124	mRNA 3'-end processing	68	1.3e-06
38	GO:0006283	transcription-coupled nucleotide-excision repair	43	1.6e-06
39	GO:1902600	hydrogen ion transmembrane transport	70	1.6e-06

Table 6: Topic 2 (Terms that appear in less than half of other topics)

	GO.ID	Term	Total	p-Value
9	GO:0000281	mitotic cytokinesis	25	1.3e-07

Table 7: Topic 3 (Terms that appear in less than half of other topics)

	GO.ID	Term	Total	p-Value
5	GO:0000398	mRNA splicing, via spliceosome	200	5.8e-19
8	GO:0070125	mitochondrial translational elongation	77	3.2e-16
9	GO:0006364	rRNA processing	124	7.0e-16
10	GO:0070124	mitochondrial translational initiation	77	2.1e-15
11	GO:0070126	mitochondrial translational termination	79	1.6e-14
15	GO:0010501	RNA secondary structure unwinding	33	1.5e-09
16	GO:0006521	regulation of cellular amino acid metabolic process	48	4.3e-09
17	GO:0000462	maturation of SSU-rRNA from tricistronic rRNA transcript (SSU-rRNA, 5.8S rRNA, LSU-rRNA)	21	4.6e-09
18	GO:0045596	negative regulation of cell differentiation	309	9.5e-09
19	GO:0002479	antigen processing and presentation of exogenous peptide antigen via MHC class I, TAP-dependent	51	1.0e-08
21	GO:0006406	mRNA export from nucleus	62	1.8e-08
22	GO:0048387	negative regulation of retinoic acid receptor signaling pathway	25	1.9e-08
23	GO:0006446	regulation of translational initiation	62	2.1e-08
24	GO:0031124	mRNA 3'-end processing	68	2.2e-08
25	GO:0006369	termination of RNA polymerase II transcription	39	3.5e-08
26	GO:0016032	viral process	525	8.1e-08
27	GO:0001731	formation of translation preinitiation complex	14	1.1e-07
28	GO:0043066	negative regulation of apoptotic process	516	1.4e-07
29	GO:0006368	transcription elongation from RNA polymerase II promoter	72	2.0e-07
30	GO:0006977	DNA damage response, signal transduction by p53 class mediator resulting in cell cycle arrest	49	2.7e-07
31	GO:0010467	gene expression	3415	3.5e-07

Table 8: Topic 4 (Terms that appear in less than half of other topics)

	GO.ID	Term	Total	p-Value
26	GO:0042769	DNA damage response, detection of DNA damage	28	3.5e-08
29	GO:0090263	positive regulation of canonical Wnt signaling pathway	86	3.0e-07
30	GO:0045814	negative regulation of gene expression, epigenetic	127	4.1e-07
34	GO:0051084	'de novo' posttranslational protein folding	35	1.3e-06

Table 9: Topic 1 (Terms that only appear in this topic)

	GO.ID	Term	Total	p-Value
27	GO:1900034	regulation of cellular response to heat	57	7.0e-08
28	GO:0051301	cell division	464	1.3e-07
29	GO:0044743	intracellular protein transmembrane import	29	2.1e-07
35	GO:0007077	mitotic nuclear envelope disassembly	34	7.2e-07
36	GO:1990542	mitochondrial transmembrane transport	37	9.7e-07

Table 10: Topic 2 (Terms that only appear in this topic)

	GO.ID	Term	Total	p-Value
9	GO:0000281	mitotic cytokinesis	25	1.3e-07

Table 11: Topic 3 (Terms that only appear in this topic)

	GO.ID	Term	Total	p-Value
15	GO:0010501	RNA secondary structure unwinding	33	1.5e-09
18	GO:0045596	negative regulation of cell differentiation	309	9.5e-09
22	GO:0048387	negative regulation of retinoic acid receptor signaling pathway	25	1.9e-08
28	GO:0043066	negative regulation of apoptotic process	516	1.4e-07
29	GO:0006368	transcription elongation from RNA polymerase II promoter	72	2.0e-07

Table 12: Topic 4 (Terms that only appear in this topic)
