# Supplementary material for: CellTree: an R/bioconductor package to infer the hierarchical structure of cell populations from single-cell RNA-seq data
Source: BMC Bioinformatics. 2016 Sep 13;17(1):363. doi: 10.1186/s12859-016-1175-6 (PMC5020541; doi:10.1186/s12859-016-1175-6)
Supplement: Additional file 7 — GO CC Terms for hESC data. Full table of enriched GO CC terms for each topic in hESC data. (PDF 35 kb) [file 12859_2016_1175_MOESM7_ESM.pdf]

	GO.ID	Term	Total	p-Value
1	GO:0070062	extracellular exosome	1659	$< 1e-30$
2	GO:0005654	nucleoplasm	2159	9.9e-27
3	GO:0005730	nucleolus	643	5.0e-26
4	GO:0005743	mitochondrial inner membrane	361	1.2e-23
5	GO:0005829	cytosol	2117	4.8e-20
6	GO:0022625	cytosolic large ribosomal subunit	31	1.0e-18
7	GO:0005747	mitochondrial respiratory chain complex I	40	1.8e-15
8	GO:0005763	mitochondrial small ribosomal subunit	24	4.5e-14
9	GO:0043209	myelin sheath	116	1.8e-13
10	GO:0005737	cytoplasm	6408	3.5e-13
11	GO:0043234	protein complex	2619	1.1e-11
12	GO:0016020	membrane	4361	6.7e-11
13	GO:0071013	catalytic step 2 spliceosome	64	7.0e-11
14	GO:0005759	mitochondrial matrix	298	4.3e-10
15	GO:0005753	mitochondrial proton-transporting ATP synthase complex	19	1.7e-09
16	GO:0005762	mitochondrial large ribosomal subunit	28	1.5e-08
17	GO:0005739	mitochondrion	1175	3.5e-08
18	GO:0005839	proteasome core complex	13	4.0e-08
19	GO:0016607	nuclear speck	143	7.9e-08
20	GO:0033290	eukaryotic 48S preinitiation complex	12	4.4e-07
21	GO:0016282	eukaryotic 43S preinitiation complex	12	5.5e-07
22	GO:0005852	eukaryotic translation initiation factor 3 complex	13	7.1e-07
23	GO:0030529	ribonucleoprotein complex	501	8.0e-07
24	GO:0098800	inner mitochondrial membrane protein complex	88	9.6e-07
25	GO:0042470	melanosome	77	1.1e-06
26	GO:0022627	cytosolic small ribosomal subunit	14	1.4e-06
27	GO:0005925	focal adhesion	260	1.5e-06
28	GO:0071011	precatalytic spliceosome	16	2.4e-06
29	GO:0044297	cell body	216	2.7e-06
30	GO:0005840	ribosome	144	4.1e-06
31	GO:0045263	proton-transporting ATP synthase complex, coupling factor F(o)	10	4.2e-06
32	GO:0005758	mitochondrial intermembrane space	54	4.3e-06
33	GO:0032040	small-subunit processome	29	6.5e-06
34	GO:0015030	Cajal body	41	7.8e-06
35	GO:0044452	nucleolar part	52	8.1e-06

Table 1: Topic 1 (All terms)

	GO.ID	Term	Total	p-Value
1	GO:0070062	extracellular exosome	1659	$< 1e-30$
2	GO:0005743	mitochondrial inner membrane	361	$< 1e-30$
3	GO:0005654	nucleoplasm	2159	3.7e-25
4	GO:0005730	nucleolus	643	7.2e-25
5	GO:0005829	cytosol	2117	2.4e-22
6	GO:0005747	mitochondrial respiratory chain complex I	40	1.3e-19
7	GO:0022625	cytosolic large ribosomal subunit	31	5.8e-19
8	GO:0043209	myelin sheath	116	1.0e-16
9	GO:0043234	protein complex	2619	3.0e-15
10	GO:0005737	cytoplasm	6408	4.2e-15
11	GO:0005763	mitochondrial small ribosomal subunit	24	4.5e-15
12	GO:0016020	membrane	4361	6.7e-13
13	GO:0071013	catalytic step 2 spliceosome	64	1.9e-11
14	GO:0005762	mitochondrial large ribosomal subunit	28	1.1e-10
15	GO:0005739	mitochondrion	1175	8.7e-10
16	GO:0016607	nuclear speck	143	9.7e-10
17	GO:0033290	eukaryotic 48S preinitiation complex	12	3.4e-09
18	GO:0005753	mitochondrial proton-transporting ATP synthase complex	19	5.2e-09
19	GO:0005759	mitochondrial matrix	298	5.9e-09
20	GO:0005852	eukaryotic translation initiation factor 3 complex	13	1.0e-08
21	GO:0005840	ribosome	144	1.1e-08
22	GO:0022627	cytosolic small ribosomal subunit	14	1.7e-08
23	GO:0005839	proteasome core complex	13	1.9e-08
24	GO:0044452	nucleolar part	52	2.1e-08
25	GO:0032040	small-subunit processome	29	2.6e-08
26	GO:0005925	focal adhesion	260	3.1e-08
27	GO:0005758	mitochondrial intermembrane space	54	4.3e-08
28	GO:0005789	endoplasmic reticulum membrane	550	7.6e-08
29	GO:0016282	eukaryotic 43S preinitiation complex	12	7.9e-08
30	GO:0042470	melanosome	77	1.6e-07
31	GO:0030686	90S preribosome	21	2.8e-07
32	GO:0098796	membrane protein complex	562	3.6e-07
33	GO:0005746	mitochondrial respiratory chain	64	5.6e-07
34	GO:0098800	inner mitochondrial membrane protein complex	88	5.6e-07
35	GO:0015030	Cajal body	41	1.6e-06
36	GO:0005643	nuclear pore	49	2.0e-06
37	GO:0030529	ribonucleoprotein complex	501	3.1e-06
38	GO:0000785	chromatin	328	5.5e-06
39	GO:0042645	mitochondrial nucleoid	35	1.2e-05
40	GO:0071011	precatalytic spliceosome	16	1.4e-05

Table 2: Topic 2 (All terms)

	GO.ID	Term	Total	p-Value
1	GO:0005654	nucleoplasm	2159	1.0e-16
2	GO:0022625	cytosolic large ribosomal subunit	31	4.6e-11
3	GO:0005737	cytoplasm	6408	7.5e-11
4	GO:0022627	cytosolic small ribosomal subunit	14	1.4e-07
5	GO:0043209	myelin sheath	116	2.4e-07
6	GO:0005829	cytosol	2117	3.1e-07
7	GO:0005813	centrosome	349	2.8e-06
8	GO:0071013	catalytic step 2 spliceosome	64	3.7e-06
9	GO:0005730	nucleolus	643	4.8e-06

Table 3: Topic 3 (All terms)

	GO.ID	Term	Total	p-Value
1	GO:0005654	nucleoplasm	2159	$< 1e-30$
2	GO:0005730	nucleolus	643	2.9e-30
3	GO:0022625	cytosolic large ribosomal subunit	31	7.8e-19
4	GO:0071013	catalytic step 2 spliceosome	64	9.1e-18
5	GO:0005634	nucleus	4479	3.9e-15
6	GO:0016607	nuclear speck	143	4.3e-14
7	GO:0044452	nucleolar part	52	8.0e-12
8	GO:0015030	Cajal body	41	4.5e-11
9	GO:0032040	small-subunit processome	29	1.2e-09
10	GO:0005852	eukaryotic translation initiation factor 3 complex	13	1.5e-09
11	GO:0033290	eukaryotic 48S preinitiation complex	12	1.2e-08
12	GO:0005743	mitochondrial inner membrane	361	1.6e-08
13	GO:0030686	90S preribosome	21	1.9e-08
14	GO:0005643	nuclear pore	49	3.0e-08
15	GO:0022627	cytosolic small ribosomal subunit	14	3.4e-08
16	GO:0030529	ribonucleoprotein complex	501	1.2e-07
17	GO:0016282	eukaryotic 43S preinitiation complex	12	2.1e-07
18	GO:0043234	protein complex	2619	2.6e-07
19	GO:0005763	mitochondrial small ribosomal subunit	24	7.2e-07
20	GO:0071011	precatalytic spliceosome	16	2.0e-06
21	GO:0043209	myelin sheath	116	5.4e-06
22	GO:0005747	mitochondrial respiratory chain complex I	40	6.0e-06
23	GO:0005689	U12-type spliceosomal complex	17	9.1e-06
24	GO:0005732	small nucleolar ribonucleoprotein complex	16	1.1e-05

Table 4: Topic 4 (All terms)

	GO.ID	Term	Total	p-Value
1	GO:0070062	extracellular exosome	1659	$< 1e-30$
4	GO:0005743	mitochondrial inner membrane	361	1.2e-23
5	GO:0005829	cytosol	2117	4.8e-20
7	GO:0005747	mitochondrial respiratory chain complex I	40	1.8e-15
8	GO:0005763	mitochondrial small ribosomal subunit	24	4.5e-14
10	GO:0005737	cytoplasm	6408	3.5e-13
11	GO:0043234	protein complex	2619	1.1e-11
12	GO:0016020	membrane	4361	6.7e-11
14	GO:0005759	mitochondrial matrix	298	4.3e-10
15	GO:0005753	mitochondrial proton-transporting ATP synthase complex	19	1.7e-09
16	GO:0005762	mitochondrial large ribosomal subunit	28	1.5e-08
17	GO:0005739	mitochondrion	1175	3.5e-08
18	GO:0005839	proteasome core complex	13	4.0e-08
19	GO:0016607	nuclear speck	143	7.9e-08
20	GO:0033290	eukaryotic 48S preinitiation complex	12	4.4e-07
21	GO:0016282	eukaryotic 43S preinitiation complex	12	5.5e-07
22	GO:0005852	eukaryotic translation initiation factor 3 complex	13	7.1e-07
23	GO:0030529	ribonucleoprotein complex	501	8.0e-07
24	GO:0098800	inner mitochondrial membrane protein complex	88	9.6e-07
25	GO:0042470	melanosome	77	1.1e-06
27	GO:0005925	focal adhesion	260	1.5e-06
28	GO:0071011	precatalytic spliceosome	16	2.4e-06
29	GO:0044297	cell body	216	2.7e-06
30	GO:0005840	ribosome	144	4.1e-06
31	GO:0045263	proton-transporting ATP synthase complex, coupling factor F(o)	10	4.2e-06
32	GO:0005758	mitochondrial intermembrane space	54	4.3e-06
33	GO:0032040	small-subunit processome	29	6.5e-06
34	GO:0015030	Cajal body	41	7.8e-06
35	GO:0044452	nucleolar part	52	8.1e-06

Table 5: Topic 1 (Terms that appear in less than half of other topics)

	GO.ID	Term	Total	p-Value
1	GO:0070062	extracellular exosome	1659	$<$ 1e-30
2	GO:0005743	mitochondrial inner membrane	361	$<$ 1e-30
5	GO:0005829	cytosol	2117	2.4e-22
6	GO:0005747	mitochondrial respiratory chain complex I	40	1.3e-19
9	GO:0043234	protein complex	2619	3.0e-15
10	GO:0005737	cytoplasm	6408	4.2e-15
11	GO:0005763	mitochondrial small ribosomal subunit	24	4.5e-15
12	GO:0016020	membrane	4361	6.7e-13
14	GO:0005762	mitochondrial large ribosomal subunit	28	1.1e-10
15	GO:0005739	mitochondrion	1175	8.7e-10
16	GO:0016607	nuclear speck	143	9.7e-10
17	GO:0033290	eukaryotic 48S preinitiation complex	12	3.4e-09
18	GO:0005753	mitochondrial proton-transporting ATP synthase complex	19	5.2e-09
19	GO:0005759	mitochondrial matrix	298	5.9e-09
20	GO:0005852	eukaryotic translation initiation factor 3 complex	13	1.0e-08
21	GO:0005840	ribosome	144	1.1e-08
23	GO:0005839	proteasome core complex	13	1.9e-08
24	GO:0044452	nucleolar part	52	2.1e-08
25	GO:0032040	small-subunit processome	29	2.6e-08
26	GO:0005925	focal adhesion	260	3.1e-08
27	GO:0005758	mitochondrial intermembrane space	54	4.3e-08
28	GO:0005789	endoplasmic reticulum membrane	550	7.6e-08
29	GO:0016282	eukaryotic 43S preinitiation complex	12	7.9e-08
30	GO:0042470	melanosome	77	1.6e-07
31	GO:0030686	90S preribosome	21	2.8e-07
32	GO:0098796	membrane protein complex	562	3.6e-07
33	GO:0005746	mitochondrial respiratory chain	64	5.6e-07
34	GO:0098800	inner mitochondrial membrane protein complex	88	5.6e-07
35	GO:0015030	Cajal body	41	1.6e-06
36	GO:0005643	nuclear pore	49	2.0e-06
37	GO:0030529	ribonucleoprotein complex	501	3.1e-06
38	GO:0000785	chromatin	328	5.5e-06
39	GO:0042645	mitochondrial nucleoid	35	1.2e-05
40	GO:0071011	precatalytic spliceosome	16	1.4e-05

Table 6: Topic 2 (Terms that appear in less than half of other topics)

	GO.ID	Term	Total	p-Value
3	GO:0005737	cytoplasm	6408	7.5e-11
6	GO:0005829	cytosol	2117	3.1e-07
7	GO:0005813	centrosome	349	2.8e-06

Table 7: Topic 3 (Terms that appear in less than half of other topics)

	GO.ID	Term	Total	p-Value
5	GO:0005634	nucleus	4479	3.9e-15
6	GO:0016607	nuclear speck	143	4.3e-14
7	GO:0044452	nucleolar part	52	8.0e-12
8	GO:0015030	Cajal body	41	4.5e-11
9	GO:0032040	small-subunit processome	29	1.2e-09
10	GO:0005852	eukaryotic translation initiation factor 3 complex	13	1.5e-09
11	GO:0033290	eukaryotic 48S preinitiation complex	12	1.2e-08
12	GO:0005743	mitochondrial inner membrane	361	1.6e-08
13	GO:0030686	90S preribosome	21	1.9e-08
14	GO:0005643	nuclear pore	49	3.0e-08
16	GO:0030529	ribonucleoprotein complex	501	1.2e-07
17	GO:0016282	eukaryotic 43S preinitiation complex	12	2.1e-07
18	GO:0043234	protein complex	2619	2.6e-07
19	GO:0005763	mitochondrial small ribosomal subunit	24	7.2e-07
20	GO:0071011	precatalytic spliceosome	16	2.0e-06
22	GO:0005747	mitochondrial respiratory chain complex I	40	6.0e-06
23	GO:0005689	U12-type spliceosomal complex	17	9.1e-06
24	GO:0005732	small nucleolar ribonucleoprotein complex	16	1.1e-05

Table 8: Topic 4 (Terms that appear in less than half of other topics)

	GO.ID	Term	Total	p-Value
29	GO:0044297	cell body	216	2.7e-06
31	GO:0045263	proton-transporting ATP synthase complex, coupling factor F(o)	10	4.2e-06

Table 9: Topic 1 (Terms that only appear in this topic)

	GO.ID	Term	Total	p-Value
28	GO:0005789	endoplasmic reticulum membrane	550	7.6e-08
32	GO:0098796	membrane protein complex	562	3.6e-07
33	GO:0005746	mitochondrial respiratory chain	64	5.6e-07
38	GO:0000785	chromatin	328	5.5e-06
39	GO:0042645	mitochondrial nucleoid	35	1.2e-05

Table 10: Topic 2 (Terms that only appear in this topic)

	GO.ID	Term	Total	p-Value
7	GO:0005813	centrosome	349	2.8e-06

Table 11: Topic 3 (Terms that only appear in this topic)

	GO.ID	Term	Total	p-Value
5	GO:0005634	nucleus	4479	3.9e-15
23	GO:0005689	U12-type spliceosomal complex	17	9.1e-06
24	GO:0005732	small nucleolar ribonucleoprotein complex	16	1.1e-05

Table 12: Topic 4 (Terms that only appear in this topic)
