# Supplementary material for: CellTree: an R/bioconductor package to infer the hierarchical structure of cell populations from single-cell RNA-seq data
Source: BMC Bioinformatics. 2016 Sep 13;17(1):363. doi: 10.1186/s12859-016-1175-6 (PMC5020541; doi:10.1186/s12859-016-1175-6)
Supplement: Additional file 8 — GO MF Terms for hESC data. Full table of enriched GO MF terms for each topic in hESC data. (PDF 31 kb) [file 12859_2016_1175_MOESM8_ESM.pdf]

	GO.ID	Term	Total	p-Value
1	GO:0044822	poly(A) RNA binding	871	$< 1e-30$
2	GO:0003735	structural constituent of ribosome	97	$< 1e-30$
3	GO:0005515	protein binding	6327	1.0e-21
4	GO:0008137	NADH dehydrogenase (ubiquinone) activity	37	1.8e-14
5	GO:0003743	translation initiation factor activity	42	3.1e-13
6	GO:0000166	nucleotide binding	1512	6.2e-12
7	GO:0051082	unfolded protein binding	69	7.2e-10
8	GO:0003729	mRNA binding	96	2.6e-08
9	GO:0004812	aminoacyl-tRNA ligase activity	38	5.2e-08
10	GO:0003723	RNA binding	1150	1.2e-07
11	GO:0004298	threonine-type endopeptidase activity	14	1.5e-07
12	GO:0019843	rRNA binding	33	1.6e-07
13	GO:0051087	chaperone binding	60	3.6e-07
14	GO:0031625	ubiquitin protein ligase binding	189	6.6e-07
15	GO:0004129	cytochrome-c oxidase activity	19	7.5e-06

Table 1: Topic 1 (All terms)

	GO.ID	Term	Total	p-Value
1	GO:0044822	poly(A) RNA binding	871	$< 1e-30$
2	GO:0003735	structural constituent of ribosome	97	$< 1e-30$
3	GO:0008137	NADH dehydrogenase (ubiquinone) activity	37	7.3e-19
4	GO:0003743	translation initiation factor activity	42	2.5e-16
5	GO:0005515	protein binding	6327	1.6e-15
6	GO:0000166	nucleotide binding	1512	1.0e-10
7	GO:0003729	mRNA binding	96	3.6e-09
8	GO:0051082	unfolded protein binding	69	2.9e-08
9	GO:0004298	threonine-type endopeptidase activity	14	7.3e-08
10	GO:0051087	chaperone binding	60	4.5e-07
11	GO:0019843	rRNA binding	33	6.6e-07
12	GO:0003723	RNA binding	1150	9.9e-07
13	GO:0005524	ATP binding	961	2.0e-06
14	GO:0008536	Ran GTPase binding	25	3.2e-06
15	GO:0004812	aminoacyl-tRNA ligase activity	38	6.4e-06

Table 2: Topic 2 (All terms)

	GO.ID	Term	Total	p-Value
1	GO:0044822	poly(A) RNA binding	871	6.2e-16
2	GO:0005515	protein binding	6327	4.6e-10
3	GO:0003735	structural constituent of ribosome	97	1.3e-07
4	GO:0005524	ATP binding	961	1.4e-06
5	GO:0031625	ubiquitin protein ligase binding	189	7.9e-06

Table 3: Topic 3 (All terms)

	GO.ID	Term	Total	p-Value
1	GO:0044822	poly(A) RNA binding	871	$< 1e-30$
2	GO:0003735	structural constituent of ribosome	97	5.3e-28
3	GO:0005515	protein binding	6327	1.5e-13
4	GO:0000166	nucleotide binding	1512	8.5e-11
5	GO:0003743	translation initiation factor activity	42	3.1e-10
6	GO:0003723	RNA binding	1150	6.5e-10
7	GO:0019843	rRNA binding	33	1.2e-09
8	GO:0003729	mRNA binding	96	2.0e-09
9	GO:0003677	DNA binding	1518	4.4e-09
10	GO:0030515	snoRNA binding	21	1.5e-07
11	GO:0004004	ATP-dependent RNA helicase activity	53	3.0e-07
12	GO:0042974	retinoic acid receptor binding	37	1.1e-06
13	GO:0004402	histone acetyltransferase activity	40	6.6e-06
14	GO:0043022	ribosome binding	32	6.9e-06
15	GO:0051082	unfolded protein binding	69	8.7e-06

Table 4: Topic 4 (All terms)

	GO.ID	Term	Total	p-Value
4	GO:0008137	NADH dehydrogenase (ubiquinone) activity	37	1.8e-14
5	GO:0003743	translation initiation factor activity	42	3.1e-13
6	GO:0000166	nucleotide binding	1512	6.2e-12
7	GO:0051082	unfolded protein binding	69	7.2e-10
8	GO:0003729	mRNA binding	96	2.6e-08
9	GO:0004812	aminoacyl-tRNA ligase activity	38	5.2e-08
10	GO:0003723	RNA binding	1150	1.2e-07
11	GO:0004298	threonine-type endopeptidase activity	14	1.5e-07
12	GO:0019843	rRNA binding	33	1.6e-07
13	GO:0051087	chaperone binding	60	3.6e-07
14	GO:0031625	ubiquitin protein ligase binding	189	6.6e-07
15	GO:0004129	cytochrome-c oxidase activity	19	7.5e-06

Table 5: Topic 1 (Terms that appear in less than half of other topics)

	GO.ID	Term	Total	p-Value
3	GO:0008137	NADH dehydrogenase (ubiquinone) activity	37	7.3e-19
4	GO:0003743	translation initiation factor activity	42	2.5e-16
6	GO:0000166	nucleotide binding	1512	1.0e-10
7	GO:0003729	mRNA binding	96	3.6e-09
8	GO:0051082	unfolded protein binding	69	2.9e-08
9	GO:0004298	threonine-type endopeptidase activity	14	7.3e-08
10	GO:0051087	chaperone binding	60	4.5e-07
11	GO:0019843	rRNA binding	33	6.6e-07
12	GO:0003723	RNA binding	1150	9.9e-07
13	GO:0005524	ATP binding	961	2.0e-06
14	GO:0008536	Ran GTPase binding	25	3.2e-06
15	GO:0004812	aminoacyl-tRNA ligase activity	38	6.4e-06

Table 6: Topic 2 (Terms that appear in less than half of other topics)

	GO.ID	Term	Total	p-Value
4	GO:0005524	ATP binding	961	1.4e-06
5	GO:0031625	ubiquitin protein ligase binding	189	7.9e-06

Table 7: Topic 3 (Terms that appear in less than half of other topics)

	GO.ID	Term	Total	p-Value
4	GO:0000166	nucleotide binding	1512	8.5e-11
5	GO:0003743	translation initiation factor activity	42	3.1e-10
6	GO:0003723	RNA binding	1150	6.5e-10
7	GO:0019843	rRNA binding	33	1.2e-09
8	GO:0003729	mRNA binding	96	2.0e-09
9	GO:0003677	DNA binding	1518	4.4e-09
10	GO:0030515	snoRNA binding	21	1.5e-07
11	GO:0004004	ATP-dependent RNA helicase activity	53	3.0e-07
12	GO:0042974	retinoic acid receptor binding	37	1.1e-06
13	GO:0004402	histone acetyltransferase activity	40	6.6e-06
14	GO:0043022	ribosome binding	32	6.9e-06
15	GO:0051082	unfolded protein binding	69	8.7e-06

Table 8: Topic 4 (Terms that appear in less than half of other topics)

	GO.ID	Term	Total	p-Value
15	GO:0004129	cytochrome-c oxidase activity	19	7.5e-06

Table 9: Topic 1 (Terms that only appear in this topic)

	GO.ID	Term	Total	p-Value
14	GO:0008536	Ran GTPase binding	25	3.2e-06

Table 10: Topic 2 (Terms that only appear in this topic)

GO.ID	Term	Total	p-Value

Table 11: Topic 3 (Terms that only appear in this topic)

	GO.ID	Term	Total	p-Value
9	GO:0003677	DNA binding	1518	4.4e-09
10	GO:0030515	snoRNA binding	21	1.5e-07
11	GO:0004004	ATP-dependent RNA helicase activity	53	3.0e-07
12	GO:0042974	retinoic acid receptor binding	37	1.1e-06
13	GO:0004402	histone acetyltransferase activity	40	6.6e-06
14	GO:0043022	ribosome binding	32	6.9e-06

Table 12: Topic 4 (Terms that only appear in this topic)
