# Supplementary material for: CellTree: an R/bioconductor package to infer the hierarchical structure of cell populations from single-cell RNA-seq data
Source: BMC Bioinformatics. 2016 Sep 13;17(1):363. doi: 10.1186/s12859-016-1175-6 (PMC5020541; doi:10.1186/s12859-016-1175-6)
Supplement: Additional file 10 — cellTree summary for mouse embryonic cells data. Full list of cell samples in the mouse embryonic cells data set, ordered and annotated by cellTree. (PDF 59 kb) [file 12859_2016_1175_MOESM10_ESM.pdf]

Ordered cells by branch

Legend: Topic #1 Topic #2 Topic #3 Topic #4

Table 1: Branch 1

node.label	cell.name	cell.group	main.topic	topics
278	GSM1112767_zy2_expression.txt	zy	3	
279	GSM1112768_zy3_expression.txt	zy	3	
280	GSM1112769_zy4_expression.txt	zy	3	
277	GSM1112766_zy1_expression.txt	zy	3	
111	GSM1112600_C57twocell_20-2_expression.txt	C57twocell	3	
109	GSM1112598_C57twocell_18-2_expression.txt	C57twocell	3	
108	GSM1112597_C57twocell_18-1_expression.txt	C57twocell	3	
110	GSM1112599_C57twocell_20-1_expression.txt	C57twocell	3	
107	GSM1112596_C57twocell_16-2_expression.txt	C57twocell	3	
106	GSM1112595_C57twocell_16-1_expression.txt	C57twocell	3	
113	GSM1112602_C57twocell_22-2_expression.txt	C57twocell	3	
112	GSM1112601_C57twocell_22-1_expression.txt	C57twocell	3	
120	GSM1112609_early2cell_3-1_expression.txt	early2cell	3	
119	GSM1112608_early2cell_2-2_expression.txt	early2cell	3	
116	GSM1112605_early2cell_1-1_expression.txt	early2cell	3	
121	GSM1112610_early2cell_3-2_expression.txt	early2cell	3	
18	GSM1112507_16cell_4-2_expression.txt	16cell	3	
115	GSM1112604_early2cell_0r-2_expression.txt	early2cell	3	
117	GSM1112606_early2cell_1-2_expression.txt	early2cell	3	
118	GSM1112607_early2cell_2-1_expression.txt	early2cell	3	
114	GSM1112603_early2cell_0r-1_expression.txt	early2cell	3	
208	GSM1112697_mid2cell_3-2_expression.txt	mid2cell	3	
207	GSM1112696_mid2cell_3-1_expression.txt	mid2cell	3	
213	GSM1112702_mid2cell_6-1_expression.txt	mid2cell	3	
215	GSM1112704_mid2cell_7-1_expression.txt	mid2cell	3	
205	GSM1112694_mid2cell_0r-1_expression.txt	mid2cell	3	
214	GSM1112703_mid2cell_6-2_expression.txt	mid2cell	3	
206	GSM1112695_mid2cell_0r-2_expression.txt	mid2cell	3	
211	GSM1112700_mid2cell_5-1_expression.txt	mid2cell	3	
216	GSM1112705_mid2cell_7-2_expression.txt	mid2cell	3	
212	GSM1112701_mid2cell_5-2_expression.txt	mid2cell	3	
172	GSM1112661_late2cell_8-2_expression.txt	late2cell	3	
210	GSM1112699_mid2cell_4-2_expression.txt	mid2cell	3	
209	GSM1112698_mid2cell_4-1_expression.txt	mid2cell	3	
168	GSM1112657_late2cell_6-2_expression.txt	late2cell	3	
171	GSM1112660_late2cell_8-1_expression.txt	late2cell	3	
169	GSM1112658_late2cell_7-1_expression.txt	late2cell	3	
167	GSM1112656_late2cell_6-1_expression.txt	late2cell	3	
165	GSM1112654_late2cell_5-1_expression.txt	late2cell	3	
170	GSM1112659_late2cell_7-2_expression.txt	late2cell	3	
174	GSM1112663_late2cell_9-2_expression.txt	late2cell	3	
166	GSM1112655_late2cell_5-2_expression.txt	late2cell	2	

173	GSM1112662_late2cell_9-1_expression.txt	late2cell	2
64	GSM1112553_4cell_4-4_expression.txt	4cell	2
61	GSM1112550_4cell_4-1_expression.txt	4cell	2
63	GSM1112552_4cell_4-3_expression.txt	4cell	2
62	GSM1112551_4cell_4-2_expression.txt	4cell	2
53	GSM1112542_4cell_1-4_expression.txt	4cell	2
56	GSM1112545_4cell_2-3_expression.txt	4cell	2
52	GSM1112541_4cell_1-2_expression.txt	4cell	2
51	GSM1112540_4cell_1-1_expression.txt	4cell	2
57	GSM1112546_4cell_2-4_expression.txt	4cell	2
55	GSM1112544_4cell_2-2_expression.txt	4cell	2
60	GSM1112549_4cell_3-4_expression.txt	4cell	2
54	GSM1112543_4cell_2-1_expression.txt	4cell	2
59	GSM1112548_4cell_3-3_expression.txt	4cell	2
58	GSM1112547_4cell_3-1_expression.txt	4cell	2
67	GSM1112556_8cell_1-4_expression.txt	8cell	2
301	GSM1278029_8cell_2pooled_split7b_expression.txt	8cell	2
68	GSM1112557_8cell_1-5_expression.txt	8cell	2
78	GSM1112567_8cell_2-8_expression.txt	8cell	2
75	GSM1112564_8cell_2-4_expression.txt	8cell	2
294	GSM1278022_8cell_14-1_smartseq2_expression.txt	8cell	2
298	GSM1278026_8cell_2pooled_split5a_expression.txt	8cell	2
307	GSM1278035_8cell_split4b_expression.txt	8cell	2
304	GSM1278032_8cell_split3a_expression.txt	8cell	2
299	GSM1278027_8cell_2pooled_split5b_expression.txt	8cell	2
76	GSM1112565_8cell_2-6_expression.txt	8cell	2
306	GSM1278034_8cell_split4a_expression.txt	8cell	2
16	GSM1112505_16cell_4-10_expression.txt	16cell	2
85	GSM1112574_8cell_5-8_expression.txt	8cell	2
300	GSM1278028_8cell_2pooled_split7a_expression.txt	8cell	2
72	GSM1112561_8cell_2-1_expression.txt	8cell	2
73	GSM1112562_8cell_2-2_expression.txt	8cell	2
86	GSM1112575_8cell_8-1_expression.txt	8cell	2
80	GSM1112569_8cell_5-2_expression.txt	8cell	2
46	GSM1112535_16cell_6-5_expression.txt	16cell	2
7	GSM1112496_16cell_1-2_expression.txt	16cell	2
305	GSM1278033_8cell_split3b_expression.txt	8cell	2
290	GSM1278018_8cell_12-2_smartseq2_expression.txt	8cell	2
292	GSM1278020_8cell_12-4_smartseq2_expression.txt	8cell	2
296	GSM1278024_8cell_14-3_smartseq2_expression.txt	8cell	2
83	GSM1112572_8cell_5-6_expression.txt	8cell	2
81	GSM1112570_8cell_5-3_expression.txt	8cell	2
4	GSM1112493_16cell_1-13_expression.txt	16cell	2
84	GSM1112573_8cell_5-7_expression.txt	8cell	2
17	GSM1112506_16cell_4-11_expression.txt	16cell	2
25	GSM1112514_16cell_4-9_expression.txt	16cell	2
3	GSM1112492_16cell_1-12_expression.txt	16cell	2
66	GSM1112555_8cell_1-2_expression.txt	8cell	2
20	GSM1112509_16cell_4-4_expression.txt	16cell	2
8	GSM1112497_16cell_1-3_expression.txt	16cell	2

302	GSM1278030_8cell_2pooled_split9a_expression.txt	8cell	2
87	GSM1112576_8cell_8-2_expression.txt	8cell	2
22	GSM1112511_16cell_4-6_expression.txt	16cell	2
19	GSM1112508_16cell_4-3_expression.txt	16cell	2
24	GSM1112513_16cell_4-8_expression.txt	16cell	2
91	GSM1112580_8cell_8-7_expression.txt	8cell	2
1	GSM1112490_16cell_1-10_expression.txt	16cell	2
33	GSM1112522_16cell_5-4_expression.txt	16cell	2
21	GSM1112510_16cell_4-5_expression.txt	16cell	2
6	GSM1112495_16cell_1-15_expression.txt	16cell	2
2	GSM1112491_16cell_1-11_expression.txt	16cell	2
92	GSM1112581_8cell_8-8_expression.txt	8cell	2
14	GSM1112503_16cell_1-9_expression.txt	16cell	2
289	GSM1278017_8cell_12-1_smartseq2_expression.txt	8cell	2
5	GSM1112494_16cell_1-14_expression.txt	16cell	2
15	GSM1112504_16cell_4-1_expression.txt	16cell	2
65	GSM1112554_8cell_1-1_expression.txt	8cell	2
11	GSM1112500_16cell_1-6_expression.txt	16cell	2
74	GSM1112563_8cell_2-3_expression.txt	8cell	2
303	GSM1278031_8cell_2pooled_split9b_expression.txt	8cell	2
89	GSM1112578_8cell_8-4_expression.txt	8cell	2
293	GSM1278021_8cell_13-1_smartseq2_expression.txt	8cell	2
77	GSM1112566_8cell_2-7_expression.txt	8cell	2
82	GSM1112571_8cell_5-4_expression.txt	8cell	2
297	GSM1278025_8cell_14-4_smartseq2_expression.txt	8cell	2
90	GSM1112579_8cell_8-6_expression.txt	8cell	2
69	GSM1112558_8cell_1-6_expression.txt	8cell	2
295	GSM1278023_8cell_14-2_smartseq2_expression.txt	8cell	2
10	GSM1112499_16cell_1-5_expression.txt	16cell	2
71	GSM1112560_8cell_1-8_expression.txt	8cell	2
79	GSM1112568_8cell_5-1_expression.txt	8cell	2
291	GSM1278019_8cell_12-3_smartseq2_expression.txt	8cell	2
287	GSM1278015_16cell_split2a_expression.txt	16cell	2
9	GSM1112498_16cell_1-4_expression.txt	16cell	2
70	GSM1112559_8cell_1-7_expression.txt	8cell	2
13	GSM1112502_16cell_1-8_expression.txt	16cell	2
29	GSM1112518_16cell_5-12_expression.txt	16cell	2
31	GSM1112520_16cell_5-2_expression.txt	16cell	2
12	GSM1112501_16cell_1-7_expression.txt	16cell	2
38	GSM1112527_16cell_5-9_expression.txt	16cell	2
30	GSM1112519_16cell_5-13_expression.txt	16cell	2
23	GSM1112512_16cell_4-7_expression.txt	16cell	2
44	GSM1112533_16cell_6-3_expression.txt	16cell	2
26	GSM1112515_16cell_5-1_expression.txt	16cell	2
32	GSM1112521_16cell_5-3_expression.txt	16cell	2
286	GSM1278014_16cell_split1b_expression.txt	16cell	2
282	GSM1278010_16cell_2pooled_split6b_expression.txt	16cell	2
281	GSM1278009_16cell_2pooled_split6a_expression.txt	16cell	2
28	GSM1112517_16cell_5-11_expression.txt	16cell	2
40	GSM1112529_16cell_6-10_expression.txt	16cell	2

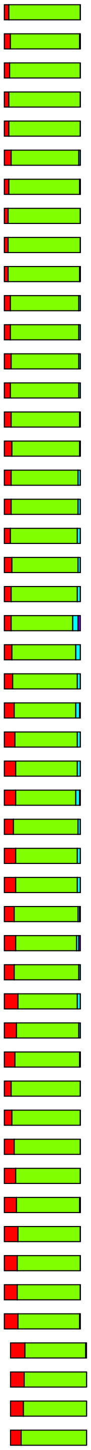

34	GSM1112523_16cell_5-5_expression.txt	16cell	2
50	GSM1112539_16cell_6-9_expression.txt	16cell	2
41	GSM1112530_16cell_6-11_expression.txt	16cell	2
39	GSM1112528_16cell_6-1_expression.txt	16cell	2
45	GSM1112534_16cell_6-4_expression.txt	16cell	2
37	GSM1112526_16cell_5-8_expression.txt	16cell	2
283	GSM1278011_16cell_2pooled_split8a_expression.txt	16cell	2
285	GSM1278013_16cell_split1a_expression.txt	16cell	2
27	GSM1112516_16cell_5-10_expression.txt	16cell	2
36	GSM1112525_16cell_5-7_expression.txt	16cell	2
288	GSM1278016_16cell_split2b_expression.txt	16cell	2
42	GSM1112531_16cell_6-12_expression.txt	16cell	2
43	GSM1112532_16cell_6-2_expression.txt	16cell	2
48	GSM1112537_16cell_6-7_expression.txt	16cell	2
284	GSM1278012_16cell_2pooled_split8b_expression.txt	16cell	2
35	GSM1112524_16cell_5-6_expression.txt	16cell	2
49	GSM1112538_16cell_6-8_expression.txt	16cell	2
47	GSM1112536_16cell_6-6_expression.txt	16cell	2
243	GSM1112732_midblast_2-13_expression.txt	midblast	1
240	GSM1112729_midblast_2-10_expression.txt	midblast	1
246	GSM1112735_midblast_2-16_expression.txt	midblast	1
248	GSM1112737_midblast_2-18_expression.txt	midblast	1
135	GSM1112624_earlyblast_2-7_expression.txt	earlyblast	1
245	GSM1112734_midblast_2-15_expression.txt	midblast	1
128	GSM1112617_earlyblast_2-19_expression.txt	earlyblast	1
151	GSM1112640_earlyblast_3-9_expression.txt	earlyblast	1
130	GSM1112619_earlyblast_2-22_expression.txt	earlyblast	1
155	GSM1112644_earlyblast_4-14_expression.txt	earlyblast	1
153	GSM1112642_earlyblast_4-12_expression.txt	earlyblast	1
252	GSM1112741_midblast_2-3_expression.txt	midblast	1
250	GSM1112739_midblast_2-23_expression.txt	midblast	1
122	GSM1112611_earlyblast_2-1_expression.txt	earlyblast	1
244	GSM1112733_midblast_2-14_expression.txt	midblast	1
158	GSM1112647_earlyblast_4-18_expression.txt	earlyblast	1
160	GSM1112649_earlyblast_4-3_expression.txt	earlyblast	1
126	GSM1112615_earlyblast_2-16_expression.txt	earlyblast	1
140	GSM1112629_earlyblast_3-13_expression.txt	earlyblast	1
242	GSM1112731_midblast_2-12_expression.txt	midblast	1
129	GSM1112618_earlyblast_2-2_expression.txt	earlyblast	1
162	GSM1112651_earlyblast_4-6_expression.txt	earlyblast	1
157	GSM1112646_earlyblast_4-17_expression.txt	earlyblast	1
255	GSM1112744_midblast_2-6_expression.txt	midblast	1
220	GSM1112709_midblast_1-12_expression.txt	midblast	1
146	GSM1112635_earlyblast_3-3_expression.txt	earlyblast	1
143	GSM1112632_earlyblast_3-16_expression.txt	earlyblast	1
137	GSM1112626_earlyblast_3-1_expression.txt	earlyblast	1
147	GSM1112636_earlyblast_3-4_expression.txt	earlyblast	1
251	GSM1112740_midblast_2-24_expression.txt	midblast	1
239	GSM1112728_midblast_2-1_expression.txt	midblast	1
148	GSM1112637_earlyblast_3-6_expression.txt	earlyblast	1

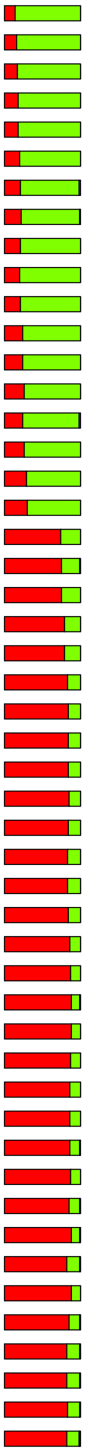

142	GSM1112631_earlyblast_3-15_expression.txt	earlyblast	1	
144	GSM1112633_earlyblast_3-17_expression.txt	earlyblast	1	
131	GSM1112620_earlyblast_2-3_expression.txt	earlyblast	1	
145	GSM1112634_earlyblast_3-2_expression.txt	earlyblast	1	
185	GSM1112674_lateblast_1-24_expression.txt	lateblast	1	
187	GSM1112676_lateblast_1-27_expression.txt	lateblast	1	
127	GSM1112616_earlyblast_2-17_expression.txt	earlyblast	1	
163	GSM1112652_earlyblast_4-8_expression.txt	earlyblast	1	
232	GSM1112721_midblast_1-24_expression.txt	midblast	1	
176	GSM1112665_lateblast_1-11_expression.txt	lateblast	1	
141	GSM1112630_earlyblast_3-14_expression.txt	earlyblast	1	
133	GSM1112622_earlyblast_2-5_expression.txt	earlyblast	1	
134	GSM1112623_earlyblast_2-6_expression.txt	earlyblast	1	
150	GSM1112639_earlyblast_3-8_expression.txt	earlyblast	1	
257	GSM1112746_midblast_2-8_expression.txt	midblast	1	
221	GSM1112710_midblast_1-13_expression.txt	midblast	1	
253	GSM1112742_midblast_2-4_expression.txt	midblast	1	
132	GSM1112621_earlyblast_2-4_expression.txt	earlyblast	1	
227	GSM1112716_midblast_1-19_expression.txt	midblast	1	

Table 2: Branch 1.1

node.label	cell.name	cell.group	main.topic	topics
247	GSM1112736_midblast_2-17_expression.txt	midblast	1	
258	GSM1112747_midblast_2-9_expression.txt	midblast	1	
164	GSM1112653_earlyblast_4-9_expression.txt	earlyblast	1	
219	GSM1112708_midblast_1-11_expression.txt	midblast	1	
256	GSM1112745_midblast_2-7_expression.txt	midblast	1	
225	GSM1112714_midblast_1-17_expression.txt	midblast	1	
156	GSM1112645_earlyblast_4-16_expression.txt	earlyblast	1	
154	GSM1112643_earlyblast_4-13_expression.txt	earlyblast	1	
236	GSM1112725_midblast_1-6_expression.txt	midblast	1	
125	GSM1112614_earlyblast_2-15_expression.txt	earlyblast	1	
224	GSM1112713_midblast_1-16_expression.txt	midblast	1	
229	GSM1112718_midblast_1-20_expression.txt	midblast	1	
235	GSM1112724_midblast_1-5_expression.txt	midblast	1	
231	GSM1112720_midblast_1-23_expression.txt	midblast	1	
261	GSM1112750_midblast_3-11_expression.txt	midblast	1	
234	GSM1112723_midblast_1-4_expression.txt	midblast	1	
218	GSM1112707_midblast_1-10_expression.txt	midblast	1	
180	GSM1112669_lateblast_1-19_expression.txt	lateblast	1	
223	GSM1112712_midblast_1-15_expression.txt	midblast	1	
222	GSM1112711_midblast_1-14_expression.txt	midblast	1	
263	GSM1112752_midblast_3-13_expression.txt	midblast	1	
138	GSM1112627_earlyblast_3-10_expression.txt	earlyblast	1	
159	GSM1112648_earlyblast_4-2_expression.txt	earlyblast	1	
238	GSM1112727_midblast_1-9_expression.txt	midblast	1	
152	GSM1112641_earlyblast_4-1_expression.txt	earlyblast	1	
175	GSM1112664_lateblast_1-10_expression.txt	lateblast	1	
217	GSM1112706_midblast_1-1_expression.txt	midblast	1	

139	GSM1112628_earlyblast_3-12_expression.txt	earlyblast	1
274	GSM1112763_midblast_3-7_expression.txt	midblast	1
237	GSM1112726_midblast_1-8_expression.txt	midblast	1
124	GSM1112613_earlyblast_2-12_expression.txt	earlyblast	1
136	GSM1112625_earlyblast_2-8_expression.txt	earlyblast	1
149	GSM1112638_earlyblast_3-7_expression.txt	earlyblast	1
228	GSM1112717_midblast_1-2_expression.txt	midblast	1
266	GSM1112755_midblast_3-17_expression.txt	midblast	1
123	GSM1112612_earlyblast_2-10_expression.txt	earlyblast	1
193	GSM1112682_lateblast_1-9_expression.txt	lateblast	1
275	GSM1112764_midblast_3-8_expression.txt	midblast	1
272	GSM1112761_midblast_3-5_expression.txt	midblast	1
186	GSM1112675_lateblast_1-26_expression.txt	lateblast	1
183	GSM1112672_lateblast_1-21_expression.txt	lateblast	1
161	GSM1112650_earlyblast_4-5_expression.txt	earlyblast	1
226	GSM1112715_midblast_1-18_expression.txt	midblast	1
269	GSM1112758_midblast_3-23_expression.txt	midblast	1
267	GSM1112756_midblast_3-18_expression.txt	midblast	1
201	GSM1112690_lateblast_2-5_expression.txt	lateblast	1
198	GSM1112687_lateblast_2-17_expression.txt	lateblast	1
268	GSM1112757_midblast_3-2_expression.txt	midblast	1
273	GSM1112762_midblast_3-6_expression.txt	midblast	1
179	GSM1112668_lateblast_1-16_expression.txt	lateblast	1
271	GSM1112760_midblast_3-4_expression.txt	midblast	1
189	GSM1112678_lateblast_1-5_expression.txt	lateblast	1
177	GSM1112666_lateblast_1-13_expression.txt	lateblast	1
265	GSM1112754_midblast_3-15_expression.txt	midblast	1
230	GSM1112719_midblast_1-22_expression.txt	midblast	1
199	GSM1112688_lateblast_2-2_expression.txt	lateblast	1
233	GSM1112722_midblast_1-3_expression.txt	midblast	1
270	GSM1112759_midblast_3-3_expression.txt	midblast	1
262	GSM1112751_midblast_3-12_expression.txt	midblast	1
188	GSM1112677_lateblast_1-4_expression.txt	lateblast	1
260	GSM1112749_midblast_3-10_expression.txt	midblast	1
178	GSM1112667_lateblast_1-14_expression.txt	lateblast	1
202	GSM1112691_lateblast_2-7_expression.txt	lateblast	1
184	GSM1112673_lateblast_1-23_expression.txt	lateblast	1
264	GSM1112753_midblast_3-14_expression.txt	midblast	1
276	GSM1112765_midblast_3-9_expression.txt	midblast	1
259	GSM1112748_midblast_3-1_expression.txt	midblast	1
182	GSM1112671_lateblast_1-20_expression.txt	lateblast	1
194	GSM1112683_lateblast_2-1_expression.txt	lateblast	1
190	GSM1112679_lateblast_1-6_expression.txt	lateblast	1
197	GSM1112686_lateblast_2-16_expression.txt	lateblast	1
191	GSM1112680_lateblast_1-7_expression.txt	lateblast	1
195	GSM1112684_lateblast_2-12_expression.txt	lateblast	1
200	GSM1112689_lateblast_2-3_expression.txt	lateblast	1
203	GSM1112692_lateblast_2-8_expression.txt	lateblast	1
196	GSM1112685_lateblast_2-14_expression.txt	lateblast	1
192	GSM1112681_lateblast_1-8_expression.txt	lateblast	1

181	GSM1112670_lateblast_1-2_expression.txt	lateblast	1	
204	GSM1112693_lateblast_2-9_expression.txt	lateblast	1	

Table 3: Branch 1.2

node.label	cell.name	cell.group	main.topic	topics
254	GSM1112743_midblast_2-5_expression.txt	midblast	1	
241	GSM1112730_midblast_2-11_expression.txt	midblast	1	
249	GSM1112738_midblast_2-2_expression.txt	midblast	1	
88	GSM1112577_8cell_8-3_expression.txt	8cell	1	
314	GSM1278042_fibroblast_20_BxC_expression.txt	fibroblast	4	
313	GSM1278041_fibroblast_19_BxC_expression.txt	fibroblast	1	
315	GSM1278043_fibroblast_21_BxC_expression.txt	fibroblast	4	
310	GSM1278038_fibroblast_15_CxB_expression.txt	fibroblast	1	
316	GSM1278044_fibroblast_22_BxC_expression.txt	fibroblast	4	
311	GSM1278039_fibroblast_16_CxB_expression.txt	fibroblast	4	
317	GSM1278045_fibroblast_9_CxB_expression.txt	fibroblast	4	
308	GSM1278036_fibroblast_13_CxB_expression.txt	fibroblast	4	
312	GSM1278040_fibroblast_17_BxC_expression.txt	fibroblast	4	
309	GSM1278037_fibroblast_14_CxB_expression.txt	fibroblast	4	
98	GSM1112587_BXC_1ng_liver_RNA_1_expression.txt	BXC	4	
99	GSM1112588_BXC_30pg_liver_RNA_0r_expression.txt	BXC	4	
97	GSM1112586_BXC_1ng_liver_RNA_0r_expression.txt	BXC	4	
93	GSM1112582_BXC_100pg_liver_RNA_1_expression.txt	BXC	4	
94	GSM1112583_BXC_100pg_liver_RNA_2_expression.txt	BXC	4	
100	GSM1112589_BXC_30pg_liver_RNA_2_expression.txt	BXC	4	
95	GSM1112584_BXC_10pg_liver_RNA_1_expression.txt	BXC	4	
96	GSM1112585_BXC_10pg_liver_RNA_2_expression.txt	BXC	4	
103	GSM1112592_BXC_liver_cell_4_expression.txt	BXC	4	
101	GSM1112590_BXC_liver_cell_1_expression.txt	BXC	4	
102	GSM1112591_BXC_liver_cell_2_expression.txt	BXC	4	
104	GSM1112593_BXC_liver_cell_5_expression.txt	BXC	4	
105	GSM1112594_BXC_liver_cell_6_expression.txt	BXC	4	
