# Supplementary material for: CellTree: an R/bioconductor package to infer the hierarchical structure of cell populations from single-cell RNA-seq data
Source: BMC Bioinformatics. 2016 Sep 13;17(1):363. doi: 10.1186/s12859-016-1175-6 (PMC5020541; doi:10.1186/s12859-016-1175-6)
Supplement: Additional file 11 — cellTree summary for mouse cortical cells data. Full list of cell samples in the mouse cortical cells data set, ordered and annotated by cellTree. (PDF 150 kb) [file 12859_2016_1175_MOESM11_ESM.pdf]

Ordered cells by branch

Legend: Topic #1 Topic #2 Topic #3 Topic #4

Table 1: Branch 1

node.label	cell.name	cell.group	main.topic	topics
96	1772067065_A02	interneurons	1	
1630	1772060226_H05	astrocytes_ependymal	1	
275	1772060224_B06	pyramidal SS	1	
135	1772071015_A01	interneurons	1	
347	1772062109_F09	pyramidal SS	1	
413	1772071014_H02	pyramidal SS	1	
1634	1772058177_G08	astrocytes_ependymal	1	
1633	1772062113_C04	astrocytes_ependymal	1	
431	1772063061_H01	pyramidal SS	1	
404	1772063074_E06	pyramidal SS	1	
120	1772058148_H04	interneurons	1	
1686	1772071017_A07	endothelial-mural	1	
544	1772062109_C04	pyramidal SS	1	
76	1772058148_D08	interneurons	1	
456	1772063078_C04	pyramidal SS	1	
530	1772063077_B02	pyramidal SS	1	
349	1772071040_H03	pyramidal SS	1	
152	1772063074_E11	interneurons	1	
405	1772063064_H01	pyramidal SS	1	
50	1772067065_D01	interneurons	1	
540	1772062111_B08	pyramidal SS	1	
39	1772071015_F03	interneurons	1	
538	1772062116_E12	pyramidal SS	1	
386	1772062115_D01	pyramidal SS	1	
504	1772062114_B06	pyramidal SS	1	
10	1772071015_C11	interneurons	1	
37	1772071017_G11	interneurons	1	
490	1772060224_F11	pyramidal SS	1	
41	1772071014_G10	interneurons	1	
499	1772063068_C02	pyramidal SS	1	
527	1772060240_A05	pyramidal SS	1	
119	1772067065_F08	interneurons	1	
526	1772060240_B09	pyramidal SS	1	
137	1772063070_B10	interneurons	1	
416	1772063078_E02	pyramidal SS	1	
1623	1772063078_D03	astrocytes_ependymal	1	
522	1772063063_A02	pyramidal SS	1	
355	1772062116_B02	pyramidal SS	1	
510	1772063068_B07	pyramidal SS	1	
1594	1772063063_E01	astrocytes_ependymal	1	
460	1772063062_A03	pyramidal SS	1	
1628	1772063070_B07	astrocytes_ependymal	1	

1612	1772067070_B12	astrocytes_ependymal	1
179	1772067064_H09	pyramidal SS	1
3	1772071017_A05	interneurons	1
113	1772071015_A05	interneurons	1
1637	1772063077_D01	astrocytes_ependymal	1
1646	1772071041_G06	astrocytes_ependymal	1
42	1772067066_B07	interneurons	1
367	1772067070_B11	pyramidal SS	1
550	1772063063_D03	pyramidal SS	1
189	1772067063_B02	pyramidal SS	1
8	1772071014_E04	interneurons	1
38	1772071014_H12	interneurons	1
44	1772071015_F07	interneurons	1
46	1772071015_C10	interneurons	1
108	1772071015_C09	interneurons	1
343	1772071015_D07	pyramidal SS	1
33	1772071015_B04	interneurons	1
115	1772067065_A01	interneurons	1
293	1772063070_B11	pyramidal SS	1
35	1772067065_D06	interneurons	1
117	1772071017_H06	interneurons	1
1	1772071015_C02	interneurons	1
155	1772071017_D01	interneurons	1
34	1772071014_D03	interneurons	1
313	1772063063_D11	pyramidal SS	1
493	1772063074_C06	pyramidal SS	1
43	1772067065_G01	interneurons	1
158	1772071014_C04	interneurons	1
40	1772071015_B11	interneurons	1
112	1772067065_G10	interneurons	1
32	1772071017_B03	interneurons	1
51	1772071015_A02	interneurons	1
244	1772063064_D02	pyramidal SS	1
31	1772071014_A07	interneurons	1
104	1772071015_H07	interneurons	1
442	1772063064_C02	pyramidal SS	1
48	1772067065_A11	interneurons	1
52	1772071014_F01	interneurons	1
114	1772067065_B10	interneurons	1
151	1772063068_F12	interneurons	1
125	1772067066_E02	interneurons	1
136	1772071017_H10	interneurons	1
106	1772071014_A06	interneurons	1
18	1772071017_E06	interneurons	1
4	1772071014_B06	interneurons	1
381	1772060240_B10	pyramidal SS	1
6	1772071017_E02	interneurons	1
131	1772067066_D04	interneurons	1
5	1772067065_H06	interneurons	1
132	1772067065_G12	interneurons	1

159	1772071017_A08	interneurons	1	
9	1772071015_D04	interneurons	1	
133	1772071017_G05	interneurons	1	
55	1772071017_D07	interneurons	1	
190	1772060240_A07	pyramidal SS	1	
230	1772067064_F02	pyramidal SS	1	
14	1772071017_A09	interneurons	1	
270	1772067074_F12	pyramidal SS	1	
68	1772071015_E06	interneurons	1	
153	1772067073_C01	interneurons	1	
175	1772067063_E09	pyramidal SS	1	
25	1772071015_B08	interneurons	1	
314	1772062115_C11	pyramidal SS	1	
520	1772060240_D04	pyramidal SS	1	
377	1772067070_H11	pyramidal SS	1	
363	1772067063_B09	pyramidal SS	1	
397	1772071041_B12	pyramidal SS	1	
59	1772071015_H11	interneurons	1	
22	1772071014_B04	interneurons	1	
213	1772067074_D05	pyramidal SS	1	
559	1772066104_H07	pyramidal SS	1	
63	1772071017_B09	interneurons	1	
346	1772067066_C03	pyramidal SS	1	
361	1772071040_F06	pyramidal SS	1	
66	1772071015_G04	interneurons	1	
149	1772071014_B10	interneurons	1	
198	1772067070_A03	pyramidal SS	1	
75	1772071017_E04	interneurons	1	
26	1772071014_C11	interneurons	1	
216	1772071017_D11	pyramidal SS	1	
557	1772067069_D09	pyramidal SS	1	
176	1772067070_D04	pyramidal SS	1	
123	1772067065_G11	interneurons	1	
356	1772071041_D08	pyramidal SS	1	
203	1772067070_G11	pyramidal SS	1	
553	1772071017_E11	pyramidal SS	1	
551	1772071041_B09	pyramidal SS	1	
58	1772071017_C07	interneurons	1	
36	1772067066_H08	interneurons	1	
212	1772067070_F03	pyramidal SS	1	
7	1772067065_B07	interneurons	1	
30	1772071017_B11	interneurons	1	
56	1772071017_A11	interneurons	1	
2	1772071017_G12	interneurons	1	
57	1772071017_B01	interneurons	1	
134	1772071017_D12	interneurons	1	
29	1772071014_H11	interneurons	1	
263	1772063065_A10	pyramidal SS	1	
167	1772067073_F04	pyramidal SS	1	
164	1772071014_E02	interneurons	1	

60	1772071014_A05	interneurons	1
144	1772071015_G09	interneurons	1
392	1772067066_G02	pyramidal SS	1
162	1772071017_A06	interneurons	1
542	1772067070_C07	pyramidal SS	1
16	1772071017_A03	interneurons	1
107	1772071015_A11	interneurons	1
186	1772067064_D02	pyramidal SS	1
399	1772067073_F02	pyramidal SS	1
284	1772060240_F12	pyramidal SS	1
339	1772063068_G04	pyramidal SS	1
374	1772071040_E02	pyramidal SS	1
13	1772071017_F09	interneurons	1
344	1772071015_D08	pyramidal SS	1
69	1772067064_E04	interneurons	1
71	1772071014_F06	interneurons	1
336	1772063062_C04	pyramidal SS	1
555	1772060225_G12	pyramidal SS	1
488	1772063065_B10	pyramidal SS	1
290	1772063070_E01	pyramidal SS	1
243	1772071041_H10	pyramidal SS	1
482	1772067073_D12	pyramidal SS	1
127	1772067065_B09	interneurons	1
21	1772071014_E06	interneurons	1
180	1772071040_B01	pyramidal SS	1
214	1772067069_G03	pyramidal SS	1
170	1772067063_C05	pyramidal SS	1
245	1772067074_C05	pyramidal SS	1
210	1772067064_G02	pyramidal SS	1
259	1772067073_G09	pyramidal SS	1
562	1772066104_A04	pyramidal SS	1
11	1772071017_D04	interneurons	1
315	1772071041_C10	pyramidal SS	1
28	1772067065_F11	interneurons	1
477	1772071041_E08	pyramidal SS	1
65	1772067065_A12	interneurons	1
294	1772071041_A05	pyramidal SS	1
295	1772060240_H05	pyramidal SS	1
248	1772067070_D06	pyramidal SS	1
177	1772067064_C07	pyramidal SS	1
165	1772067069_H02	pyramidal SS	1
77	1772067065_F07	interneurons	1
191	1772067073_A01	pyramidal SS	1
172	1772067064_C12	pyramidal SS	1
224	1772071041_E01	pyramidal SS	1
221	1772067073_F08	pyramidal SS	1
297	1772063065_G10	pyramidal SS	1
332	1772066104_F10	pyramidal SS	1
27	1772067066_E10	interneurons	1
328	1772063065_D02	pyramidal SS	1

70	1772071015_C01	interneurons	1
266	1772063068_F07	pyramidal SS	1
500	1772063068_E08	pyramidal SS	1
12	1772071017_D06	interneurons	1
365	1772067073_B06	pyramidal SS	1
122	1772067065_B12	interneurons	1
389	1772067070_D10	pyramidal SS	1
62	1772071017_C08	interneurons	1
205	1772067069_G06	pyramidal SS	1
24	1772071017_E10	interneurons	1
318	1772067069_C05	pyramidal SS	1
200	1772067064_B05	pyramidal SS	1
519	1772060240_E09	pyramidal SS	1
283	1772071041_A06	pyramidal SS	1
319	1772063065_C09	pyramidal SS	1
171	1772067069_C12	pyramidal SS	1
199	1772071017_C06	pyramidal SS	1
74	1772071014_E05	interneurons	1
219	1772071041_D09	pyramidal SS	1
390	1772071040_A01	pyramidal SS	1
480	1772067070_F11	pyramidal SS	1
225	1772071041_G07	pyramidal SS	1
322	1772067073_B04	pyramidal SS	1
139	1772071015_E10	interneurons	1
474	1772067074_D06	pyramidal SS	1
187	1772067069_H10	pyramidal SS	1
128	1772067065_F10	interneurons	1
211	1772071040_E01	pyramidal SS	1
547	1772071041_C06	pyramidal SS	1
67	1772067066_B02	interneurons	1
157	1772071015_E08	interneurons	1
215	1772067070_E11	pyramidal SS	1
148	1772071017_C10	interneurons	1
257	1772067063_B12	pyramidal SS	1
252	1772071041_C05	pyramidal SS	1
298	1772067074_D10	pyramidal SS	1
253	1772067070_G12	pyramidal SS	1
292	1772063068_H12	pyramidal SS	1
183	1772067069_D10	pyramidal SS	1
161	1772067065_A08	interneurons	1
278	1772071040_G09	pyramidal SS	1
305	1772071041_B01	pyramidal SS	1
73	1772071017_C05	interneurons	1
126	1772067066_A11	interneurons	1
335	1772067073_F10	pyramidal SS	1
208	1772067066_A06	pyramidal SS	1
188	1772071017_G01	pyramidal SS	1
209	1772071040_G08	pyramidal SS	1
251	1772067074_F03	pyramidal SS	1
142	1772071015_F05	interneurons	1

178	1772067069_A11	pyramidal SS	1
324	1772071040_H01	pyramidal SS	1
535	1772067069_B11	pyramidal SS	1
260	1772067069_E04	pyramidal SS	1
197	1772067070_C01	pyramidal SS	1
227	1772071041_H09	pyramidal SS	1
545	1772071017_G07	pyramidal SS	1
124	1772067066_A10	interneurons	1
541	1772071040_H11	pyramidal SS	1
373	1772067070_G04	pyramidal SS	1
193	1772067074_A06	pyramidal SS	1
552	1772067066_D09	pyramidal SS	1
207	1772071041_H11	pyramidal SS	1
543	1772071017_A02	pyramidal SS	1
226	1772071041_G03	pyramidal SS	1
366	1772067073_G04	pyramidal SS	1
376	1772071040_B06	pyramidal SS	1
375	1772071041_D11	pyramidal SS	1
220	1772071041_H12	pyramidal SS	1
546	1772067074_A09	pyramidal SS	1
258	1772067063_H04	pyramidal SS	1
554	1772071017_B07	pyramidal SS	1
53	1772071015_D06	interneurons	1
537	1772071040_D07	pyramidal SS	1
228	1772071041_G08	pyramidal SS	1
237	1772071041_A04	pyramidal SS	1
163	1772071014_F12	interneurons	1
17	1772071017_F07	interneurons	1
279	1772071041_E10	pyramidal SS	1
181	1772067064_B04	pyramidal SS	1
274	1772063065_F03	pyramidal SS	1
539	1772067070_D03	pyramidal SS	1
395	1772067070_D05	pyramidal SS	1
471	1772063071_E09	pyramidal SS	1
166	1772063071_B07	pyramidal SS	1
173	1772071041_F07	pyramidal SS	1
202	1772067064_C02	pyramidal SS	1
271	1772067064_A01	pyramidal SS	1
276	1772071041_H05	pyramidal SS	1
15	1772071015_C08	interneurons	1
302	1772067074_F10	pyramidal SS	1
352	1772067073_E06	pyramidal SS	1
204	1772067074_F07	pyramidal SS	1
528	1772060240_G02	pyramidal SS	1
64	1772067066_A09	interneurons	1
201	1772067073_C12	pyramidal SS	1
334	1772063068_H01	pyramidal SS	1
308	1772063065_C08	pyramidal SS	1
312	1772060240_D06	pyramidal SS	1
61	1772071017_A10	interneurons	1

141	1772071041_B06	interneurons	1
20	1772071017_B05	interneurons	1
169	1772067066_E04	pyramidal SS	1
536	1772071040_C06	pyramidal SS	1
19	1772067066_C10	interneurons	1
23	1772067066_B09	interneurons	1
255	1772067074_B01	pyramidal SS	1
362	1772067074_G06	pyramidal SS	1
192	1772067064_B01	pyramidal SS	1
359	1772067074_A02	pyramidal SS	1
518	1772060240_E07	pyramidal SS	1
300	1772063065_D12	pyramidal SS	1
265	1772067069_F05	pyramidal SS	1
441	1772063068_F09	pyramidal SS	1
345	1772067064_A12	pyramidal SS	1
301	1772063065_B02	pyramidal SS	1
231	1772067064_F01	pyramidal SS	1
385	1772063065_F01	pyramidal SS	1
420	1772063068_E07	pyramidal SS	1
269	1772063065_F02	pyramidal SS	1
478	1772063065_D09	pyramidal SS	1
549	1772067063_C06	pyramidal SS	1
487	1772063068_G01	pyramidal SS	1
428	1772062115_C08	pyramidal SS	1
439	1772063061_A02	pyramidal SS	1
492	1772071040_H08	pyramidal SS	1
407	1772067064_H05	pyramidal SS	1
388	1772063065_A01	pyramidal SS	1
310	1772063061_A05	pyramidal SS	1
247	1772063068_F11	pyramidal SS	1
380	1772063068_C01	pyramidal SS	1
311	1772063068_F02	pyramidal SS	1
238	1772063068_B01	pyramidal SS	1
406	1772071014_C08	pyramidal SS	1
72	1772071017_D10	interneurons	1
489	1772063065_H05	pyramidal SS	1
529	1772063065_A06	pyramidal SS	1
563	1772066104_A01	pyramidal SS	1
450	1772063078_F03	pyramidal SS	1
249	1772067074_B09	pyramidal SS	1
516	1772060240_F09	pyramidal SS	1
340	1772063065_A03	pyramidal SS	1
49	1772071015_G12	interneurons	1
524	1772060240_A02	pyramidal SS	1
398	1772063077_E05	pyramidal SS	1
45	1772067065_E07	interneurons	1
47	1772067063_E06	interneurons	1
561	1772066104_G09	pyramidal SS	1
459	1772062115_E09	pyramidal SS	1
463	1772067063_H06	pyramidal SS	1

403	1772063061_A03	pyramidal SS	1	
469	1772067073_D11	pyramidal SS	1	
408	1772063068_A12	pyramidal SS	1	
531	1772063068_E02	pyramidal SS	1	
87	1772071040_A12	interneurons	1	
432	1772063061_B06	pyramidal SS	1	
453	1772062113_D06	pyramidal SS	1	
321	1772063068_G10	pyramidal SS	1	
184	1772062109_B03	pyramidal SS	1	
449	1772063070_F01	pyramidal SS	1	
372	1772062118_B11	pyramidal SS	1	
384	1772063077_C12	pyramidal SS	1	
281	1772062128_D10	pyramidal SS	1	
401	1772063061_A10	pyramidal SS	1	
387	1772062118_A01	pyramidal SS	1	
272	1772063064_B11	pyramidal SS	1	
402	1772062128_C12	pyramidal SS	1	
85	1772062115_G05	interneurons	1	
54	1772071017_B02	interneurons	1	
525	1772060240_C06	pyramidal SS	1	
411	1772067065_G07	pyramidal SS	1	
462	1772062113_G03	pyramidal SS	1	
461	1772062114_G07	pyramidal SS	1	
485	1772063068_E12	pyramidal SS	1	
444	1772063068_B04	pyramidal SS	1	
289	1772062128_D12	pyramidal SS	1	
235	1772063068_A01	pyramidal SS	1	
452	1772063062_D12	pyramidal SS	1	
358	1772062109_E09	pyramidal SS	1	
511	1772063068_A07	pyramidal SS	1	
89	1772063078_F12	interneurons	1	
418	1772063061_E05	pyramidal SS	1	
494	1772063065_A08	pyramidal SS	1	
138	1772063065_C02	interneurons	1	
426	1772063061_G12	pyramidal SS	1	
433	1772063071_E12	pyramidal SS	1	
451	1772062115_D06	pyramidal SS	1	
533	1772063068_C06	pyramidal SS	1	
160	1772063064_B06	interneurons	1	
94	1772062111_G03	interneurons	1	
267	1772063068_A02	pyramidal SS	1	
341	1772062128_A09	pyramidal SS	1	
491	1772062113_A04	pyramidal SS	1	
498	1772063078_E04	pyramidal SS	1	
282	1772063078_B12	pyramidal SS	1	
304	1772063068_E06	pyramidal SS	1	
502	1772063070_D09	pyramidal SS	1	
473	1772063070_D11	pyramidal SS	1	
464	1772062114_A02	pyramidal SS	1	
156	1772058148_H08	interneurons	1	

264	1772063068_D04	pyramidal SS	1	
497	1772062113_B05	pyramidal SS	1	
532	1772063068_C11	pyramidal SS	1	
338	1772063068_C10	pyramidal SS	1	
242	1772063077_D06	pyramidal SS	1	
505	1772063065_B05	pyramidal SS	1	
232	1772063063_H02	pyramidal SS	1	
285	1772063065_D04	pyramidal SS	1	
241	1772063062_H11	pyramidal SS	1	
82	1772063068_G07	interneurons	1	
470	1772063071_D09	pyramidal SS	1	
382	1772060240_B01	pyramidal SS	1	
560	1772066104_H05	pyramidal SS	1	
424	1772063071_A03	pyramidal SS	1	
320	1772063065_E02	pyramidal SS	1	
206	1772067070_F07	pyramidal SS	1	
394	1772063071_E01	pyramidal SS	1	
466	1772062113_E03	pyramidal SS	1	
105	1772062109_H07	interneurons	1	
472	1772062113_D03	pyramidal SS	1	
195	1772071041_G09	pyramidal SS	1	
286	1772062116_B11	pyramidal SS	1	

Table 2: Branch 1.1

node.label	cell.name	cell.group	main.topic	topics
97	1772062128_G04	interneurons	1	
229	1772067070_G08	pyramidal SS	1	
1519	1772062118_H11	astrocytes_ependymal	1	
475	1772063063_A07	pyramidal SS	1	
409	1772062109_H06	pyramidal SS	1	
1687	1772063071_G10	endothelial-mural	1	
412	1772062128_D01	pyramidal SS	1	
458	1772062109_G11	pyramidal SS	1	
440	1772063061_B01	pyramidal SS	1	
465	1772062109_D07	pyramidal SS	1	
425	1772058171_C07	pyramidal SS	1	
223	1772071040_F05	pyramidal SS	1	
414	1772063061_H04	pyramidal SS	1	
445	1772063077_C06	pyramidal SS	1	
129	1772063063_H03	interneurons	1	
435	1772058177_H05	pyramidal SS	1	
143	1772062115_G06	interneurons	1	
437	1772058177_H07	pyramidal SS	1	
350	1772063062_D03	pyramidal SS	1	
330	1772063062_F05	pyramidal SS	1	
154	1772058177_D04	interneurons	1	
507	1772063078_E06	pyramidal SS	1	
92	1772062128_D11	interneurons	1	
327	1772063071_E07	pyramidal SS	1	

438	1772058171_E01	pyramidal SS	1
280	1772062128_D07	pyramidal SS	1
287	1772063078_A04	pyramidal SS	1
484	1772062115_E10	pyramidal SS	1
240	1772063063_G11	pyramidal SS	1
109	1772063071_A06	interneurons	1
333	1772058177_G10	pyramidal SS	1
194	1772062116_B04	pyramidal SS	1
130	1772071017_A01	interneurons	1
118	1772071014_A12	interneurons	1
481	1772063063_H04	pyramidal SS	1
443	1772063061_G01	pyramidal SS	1
145	1772071017_G02	interneurons	1
342	1772063071_F08	pyramidal SS	1
548	1772067070_D09	pyramidal SS	1
495	1772063071_B03	pyramidal SS	1
329	1772063071_C08	pyramidal SS	1
496	1772063071_E05	pyramidal SS	1
262	1772063071_H01	pyramidal SS	1
234	1772063071_G03	pyramidal SS	1
95	1772063061_H03	interneurons	1
256	1772062115_G01	pyramidal SS	1
79	1772063077_B03	interneurons	1
467	1772067073_H12	pyramidal SS	1
454	1772063062_D06	pyramidal SS	1
140	1772062115_H01	interneurons	1
100	1772062128_B10	interneurons	1
378	1772063071_C01	pyramidal SS	1
309	1772062116_A06	pyramidal SS	1
182	1772063063_E12	pyramidal SS	1
236	1772063071_E06	pyramidal SS	1
168	1772067069_A03	pyramidal SS	1
233	1772063065_C11	pyramidal SS	1
354	1772071040_D11	pyramidal SS	1
369	1772067063_A01	pyramidal SS	1
457	1772063068_C12	pyramidal SS	1
364	1772071041_F02	pyramidal SS	1
250	1772063077_D12	pyramidal SS	1
400	1772062111_A09	pyramidal SS	1
299	1772071040_B12	pyramidal SS	1
277	1772062109_E06	pyramidal SS	1
288	1772063068_D03	pyramidal SS	1
446	1772063061_H08	pyramidal SS	1
337	1772062109_A11	pyramidal SS	1
476	1772062111_C10	pyramidal SS	1
503	1772063077_B04	pyramidal SS	1
448	1772063063_E11	pyramidal SS	1
217	1772071041_E12	pyramidal SS	1
360	1772067074_C04	pyramidal SS	1
323	1772067074_D08	pyramidal SS	1

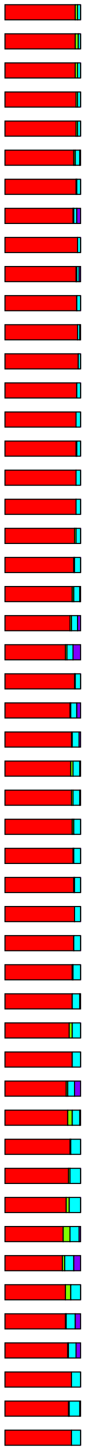

357	1772067064_G12	pyramidal SS	1
353	1772071040_F10	pyramidal SS	1
111	1772067066_H09	interneurons	1
556	1772071041_A09	pyramidal SS	1
316	1772060225_C12	pyramidal SS	1
93	1772063078_B02	interneurons	1
239	1772063070_F03	pyramidal SS	1
150	1772062128_G06	interneurons	1
98	1772062109_H03	interneurons	1
1489	1772062128_C09	endothelial-mural	1
1445	1772063068_B12	endothelial-mural	1
348	1772060224_C03	pyramidal SS	1
90	1772062115_C05	interneurons	1
427	1772062128_H06	pyramidal SS	1
325	1772063062_F07	pyramidal SS	1
306	1772060226_F11	pyramidal SS	1
296	1772063065_F04	pyramidal SS	1
303	1772060224_H02	pyramidal SS	1
1688	1772058148_C03	endothelial-mural	1
1680	1772062111_E09	endothelial-mural	1
1444	1772063063_H05	endothelial-mural	1
1441	1772063065_D08	endothelial-mural	1
1682	1772063074_H03	endothelial-mural	1
1683	1772063062_H10	endothelial-mural	1
1439	1772063061_B10	endothelial-mural	1
1089	1772063068_F06	oligodendrocytes	1
506	1772063078_D10	pyramidal SS	1
509	1772063070_F09	pyramidal SS	1
1443	1772063065_B12	endothelial-mural	1
1446	1772062109_G06	endothelial-mural	1
1675	1772063063_G07	endothelial-mural	1
1670	1772062113_D02	endothelial-mural	1
517	1772062128_A11	pyramidal SS	1
146	1772062111_C11	interneurons	1
307	1772067069_C08	pyramidal SS	1
1305	1772063063_H11	microglia	1
1327	1772060240_H10	microglia	1
1369	1772063063_B01	endothelial-mural	1
1330	1772063063_A03	microglia	1
1689	1772063061_D09	endothelial-mural	1
1496	1772066104_A09	endothelial-mural	1
1690	1772063068_D01	endothelial-mural	1
1390	1772062111_D07	endothelial-mural	1
1685	1772063078_G10	endothelial-mural	1
512	1772062114_G09	pyramidal SS	1
1486	1772060240_B11	endothelial-mural	1
1674	1772067064_D03	endothelial-mural	1
1368	1772062113_D11	endothelial-mural	1
1660	1772062111_B09	endothelial-mural	1
1365	1772062113_G08	endothelial-mural	1

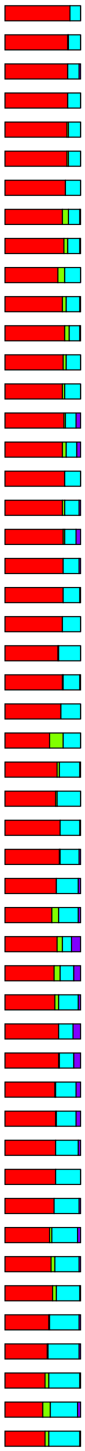

1463	1772062111_A11	endothelial-mural	1
1461	1772062109_E02	endothelial-mural	1
1351	1772063061_G03	endothelial-mural	1
1437	1772063065_D10	endothelial-mural	1
1429	1772063070_A05	endothelial-mural	1
1359	1772067070_H08	endothelial-mural	1
1462	1772060224_F01	endothelial-mural	1
1663	1772062111_D02	endothelial-mural	3
1447	1772062116_H04	endothelial-mural	3
1658	1772063062_D04	endothelial-mural	3
1405	1772063063_C11	endothelial-mural	1
1651	1772060240_C12	endothelial-mural	3
1676	1772067063_E08	endothelial-mural	3
1673	1772062116_E06	endothelial-mural	3
1666	1772063077_B05	endothelial-mural	3
1454	1772062116_H07	endothelial-mural	3
1403	1772062113_C01	endothelial-mural	3
1398	1772062114_A09	endothelial-mural	3
1147	1772063070_D01	oligodendrocytes	3
1408	1772063063_G05	endothelial-mural	3
1475	1772062115_G08	endothelial-mural	3
1419	1772067074_H09	endothelial-mural	3
1497	1772060240_G06	endothelial-mural	3
1455	1772063070_F02	endothelial-mural	3
1404	1772067073_A12	endothelial-mural	3
1358	1772060240_A03	endothelial-mural	3
1460	1772067074_D07	endothelial-mural	3
1493	1772067063_E01	endothelial-mural	3
1389	1772062109_D05	endothelial-mural	3
1498	1772062109_B04	endothelial-mural	3
1668	1772062113_H10	endothelial-mural	3
1664	1772063077_A05	endothelial-mural	3
1483	1772062128_E01	endothelial-mural	3
1421	1772067073_F07	endothelial-mural	3
1406	1772062109_B07	endothelial-mural	3
1681	1772062111_F01	endothelial-mural	3
1656	1772063062_D11	endothelial-mural	3
1436	1772063070_G07	endothelial-mural	3
1414	1772067074_H04	endothelial-mural	3
1432	1772060240_D07	endothelial-mural	3
1412	1772067063_A07	endothelial-mural	3
1450	1772067063_D05	endothelial-mural	3
1420	1772071041_H04	endothelial-mural	3
1427	1772067074_F11	endothelial-mural	3
1501	1772067069_H12	endothelial-mural	3
1653	1772067069_E08	endothelial-mural	3
1361	1772063071_A01	endothelial-mural	3
1411	1772071040_F09	endothelial-mural	3
1409	1772067069_E07	endothelial-mural	3
1400	1772063070_B04	endothelial-mural	3

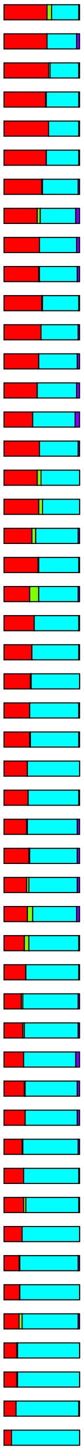

1478	1772060225_H04	endothelial-mural	3
1363	1772067070_C05	endothelial-mural	3
1669	1772067074_G05	endothelial-mural	3
1410	1772067063_A11	endothelial-mural	3
1433	1772067063_B03	endothelial-mural	3
1671	1772067074_C09	endothelial-mural	3
1503	1772063071_G02	endothelial-mural	3
1350	1772063078_H11	endothelial-mural	3
1378	1772058177_F10	endothelial-mural	3
1476	1772062114_H08	endothelial-mural	3
1381	1772058177_F12	endothelial-mural	3
1440	1772062115_F01	endothelial-mural	3
1500	1772063071_D02	endothelial-mural	3
1417	1772063071_D11	endothelial-mural	3
1397	1772063070_F11	endothelial-mural	3
1467	1772063071_F12	endothelial-mural	3
1492	1772063071_B06	endothelial-mural	3
1387	1772063079_B05	endothelial-mural	3
1418	1772071040_E11	endothelial-mural	3
1352	1772063071_D01	endothelial-mural	3
1499	1772063071_B12	endothelial-mural	3
1355	1772063062_H12	endothelial-mural	3
1360	1772063079_C02	endothelial-mural	3
1395	1772063079_H12	endothelial-mural	3
1667	1772058171_E08	endothelial-mural	3
1456	1772060224_C09	endothelial-mural	3
1407	1772071040_D01	endothelial-mural	3
1394	1772063079_C11	endothelial-mural	3
1453	1772063079_A07	endothelial-mural	3
1382	1772058177_G12	endothelial-mural	3
1424	1772067074_H08	endothelial-mural	3
1269	1772060225_E03	microglia	3
1473	1772063064_A08	endothelial-mural	3
1662	1772063061_C07	endothelial-mural	3
1452	1772058177_F11	endothelial-mural	3
1348	1772063064_A11	endothelial-mural	3
1495	1772062111_F09	endothelial-mural	3
1354	1772062111_E02	endothelial-mural	3
1391	1772063077_D11	endothelial-mural	3
1469	1772058177_A08	endothelial-mural	3
1647	1772063079_E03	endothelial-mural	3
1399	1772062111_H12	endothelial-mural	3
1426	1772063070_A07	endothelial-mural	3
1659	1772058177_G01	endothelial-mural	3
1402	1772058177_G07	endothelial-mural	3
1428	1772063064_B04	endothelial-mural	3
1649	1772063078_E07	endothelial-mural	3
1661	1772062113_E06	endothelial-mural	3
1386	1772062128_D06	endothelial-mural	3
1373	1772058177_G06	endothelial-mural	3

1396	1772060224_E05	endothelial-mural	3
1431	1772063070_E06	endothelial-mural	3
1375	1772058177_H02	endothelial-mural	3
1370	1772058171_E12	endothelial-mural	3
1449	1772058171_G04	endothelial-mural	3
1377	1772058171_E11	endothelial-mural	3
1384	1772058171_H05	endothelial-mural	3
1380	1772058171_G07	endothelial-mural	3
1430	1772071040_B11	endothelial-mural	3
1494	1772058171_A01	endothelial-mural	3
1416	1772067073_B09	endothelial-mural	3
1451	1772058177_A05	endothelial-mural	3
1458	1772058171_A12	endothelial-mural	3
1379	1772058171_G09	endothelial-mural	3
1362	1772063079_A09	endothelial-mural	3
1477	1772063071_G12	endothelial-mural	3
1459	1772067073_H10	endothelial-mural	3
1464	1772058171_A06	endothelial-mural	3
1364	1772063079_A11	endothelial-mural	3
1347	1772063071_E02	endothelial-mural	3
1376	1772058177_D01	endothelial-mural	3
1392	1772062113_H07	endothelial-mural	3
1471	1772058177_B01	endothelial-mural	3
1356	1772058177_C08	endothelial-mural	3
1470	1772060224_H01	endothelial-mural	3
1371	1772063064_F06	endothelial-mural	3
1401	1772063064_E04	endothelial-mural	3
1353	1772063064_B05	endothelial-mural	3
1479	1772058177_H03	endothelial-mural	3
1457	1772067073_H05	endothelial-mural	3
1385	1772058171_G05	endothelial-mural	3
1650	1772058177_B10	endothelial-mural	3
1679	1772063074_C04	endothelial-mural	3
1435	1772060224_A05	endothelial-mural	3
1425	1772058148_F12	endothelial-mural	3
1415	1772058148_E02	endothelial-mural	3
1268	1772058177_A07	microglia	3
1288	1772058177_E01	microglia	3
1434	1772058148_F05	endothelial-mural	3
1366	1772062128_G09	endothelial-mural	3
1393	1772062111_G12	endothelial-mural	3
1665	1772063079_F03	endothelial-mural	3
1648	1772058177_B08	endothelial-mural	3
1677	1772058148_G02	endothelial-mural	3
1349	1772060224_B09	endothelial-mural	3
1465	1772060224_F04	endothelial-mural	3
1442	1772058148_F10	endothelial-mural	3
1263	1772058171_F04	microglia	3
1657	1772058148_F01	endothelial-mural	3
1491	1772058177_H04	endothelial-mural	3

1691	1772058148_F03	endothelial-mural	3
1487	1772063078_A02	endothelial-mural	3
1472	1772062111_B12	endothelial-mural	3
1357	1772058148_A07	endothelial-mural	3
1438	1772060226_E09	endothelial-mural	3
1652	1772067073_A03	endothelial-mural	3
1374	1772058177_H06	endothelial-mural	3
1331	1772060226_H12	microglia	3
1332	1772062118_F03	microglia	3
1282	1772060226_E03	microglia	3
1321	1772062111_F12	microglia	3
1278	1772058148_D12	microglia	3
1295	1772062111_H06	microglia	3
1423	1772058148_E09	endothelial-mural	3
1590	1772058148_G05	astrocytes_ependymal	3
1334	1772062128_H04	microglia	3
1588	1772062113_E12	astrocytes_ependymal	3
1474	1772063064_B02	endothelial-mural	3
1341	1772060224_B07	microglia	3
1340	1772058177_E03	microglia	3
1466	1772060224_H12	endothelial-mural	3
1542	1772062128_B12	astrocytes_ependymal	3
1284	1772058177_A12	microglia	3
1654	1772058148_D03	endothelial-mural	3
1655	1772058148_D10	endothelial-mural	3
1343	1772062118_D09	microglia	3
1309	1772062111_A05	microglia	3
1273	1772058177_C03	microglia	3
1638	1772058148_B12	astrocytes_ependymal	3
1275	1772062128_F05	microglia	3
1292	1772063074_B03	microglia	3
1317	1772062111_G06	microglia	3
1383	1772058171_F01	endothelial-mural	3
1298	1772062115_A07	microglia	3
1289	1772058177_B11	microglia	3
1293	1772058171_F11	microglia	3
1310	1772060224_B12	microglia	3
1314	1772063074_F08	microglia	3
1299	1772062115_C02	microglia	3
1286	1772058171_C05	microglia	3
1344	1772062118_F11	microglia	3
1336	1772060225_F05	microglia	3
1547	1772058171_A08	astrocytes_ependymal	3
1481	1772060224_D12	endothelial-mural	3
1335	1772062118_E06	microglia	3
1306	1772062118_A11	microglia	3
1290	1772058177_F07	microglia	3
1422	1772058148_C04	endothelial-mural	3
1413	1772058148_D04	endothelial-mural	3
1315	1772058177_F04	microglia	3

1448	1772063062_G06	endothelial-mural	3
1280	1772066104_B07	microglia	3
1296	1772063061_F10	microglia	3
1311	1772067064_D12	microglia	3
1319	1772062109_A06	microglia	3
1301	1772062109_D06	microglia	3
1485	1772062128_C11	endothelial-mural	3
1502	1772063062_A05	endothelial-mural	3
1318	1772062109_D12	microglia	3
1297	1772062116_D02	microglia	3
1620	1772063062_E03	astrocytes_ependymal	3
1482	1772063062_B10	endothelial-mural	3
1333	1772063062_D07	microglia	3
1614	1772058148_E06	astrocytes_ependymal	3
1291	1772058177_G04	microglia	3
1312	1772060240_F05	microglia	3
1324	1772067064_B09	microglia	3
1316	1772067074_D04	microglia	3
1266	1772058148_F06	microglia	3
1287	1772062128_A07	microglia	3
1372	1772058148_H05	endothelial-mural	3
1329	1772063077_D08	microglia	3
1303	1772062118_F04	microglia	3
1264	1772060226_D02	microglia	3
1271	1772058177_D11	microglia	3
1345	1772062111_D12	microglia	3
1313	1772063077_E03	microglia	3
1285	1772062128_C02	microglia	3
1672	1772063077_B06	endothelial-mural	3
1323	1772063074_H12	microglia	3
1326	1772067070_C02	microglia	3
1300	1772062109_E12	microglia	3
1328	1772062116_G09	microglia	3
1270	1772058177_D10	microglia	3
1468	1772062111_A08	endothelial-mural	3
1484	1772062128_A02	endothelial-mural	3
1639	1772063063_C12	astrocytes_ependymal	3
1342	1772060226_H02	microglia	1
1490	1772062116_C03	endothelial-mural	3
1488	1772058148_B03	endothelial-mural	3
1294	1772062113_A11	microglia	1
1367	1772060240_D11	endothelial-mural	1
1267	1772058177_A09	microglia	1
1320	1772062113_E05	microglia	1
1388	1772062109_H08	endothelial-mural	3
1272	1772062109_D01	microglia	1
1265	1772062109_F11	microglia	1
421	1772063061_C11	pyramidal SS	1
1325	1772060224_H07	microglia	1
1337	1772060226_F12	microglia	1

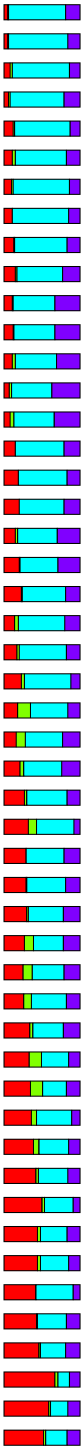

1276	1772067070_C04	microglia	1	
1304	1772063078_G02	microglia	1	
1480	1772063064_F09	endothelial-mural	1	
1307	1772060224_F07	microglia	1	
1641	1772063065_A11	astrocytes_ependymal	1	
1553	1772063062_D09	astrocytes_ependymal	1	
1598	1772063077_B01	astrocytes_ependymal	1	
1283	1772060240_H11	microglia	3	
1339	1772062109_C05	microglia	3	
1308	1772063077_F06	microglia	4	
1613	1772058148_G03	astrocytes_ependymal	3	
1346	1772062128_B03	microglia	3	
1624	1772060225_C06	astrocytes_ependymal	3	
1602	1772063063_F12	astrocytes_ependymal	4	
1279	1772058148_A05	microglia	3	
1580	1772063063_F04	astrocytes_ependymal	4	
1631	1772058148_B11	astrocytes_ependymal	4	
1551	1772063071_H09	astrocytes_ependymal	4	
1534	1772062114_B05	astrocytes_ependymal	4	
1540	1772063062_B05	astrocytes_ependymal	4	
1509	1772071015_H09	astrocytes_ependymal	4	
1515	1772071015_A08	astrocytes_ependymal	4	
1569	1772062114_D04	astrocytes_ependymal	4	
1549	1772062128_E04	astrocytes_ependymal	4	
1530	1772062109_F07	astrocytes_ependymal	4	
1554	1772063068_D06	astrocytes_ependymal	4	
1533	1772062114_D03	astrocytes_ependymal	4	
1543	1772062114_E01	astrocytes_ependymal	4	
1581	1772063074_D02	astrocytes_ependymal	4	
1593	1772062128_C04	astrocytes_ependymal	4	
1524	1772071014_D07	astrocytes_ependymal	4	
1579	1772063061_C12	astrocytes_ependymal	4	
1618	1772062118_C05	astrocytes_ependymal	4	
1607	1772063063_G08	astrocytes_ependymal	4	
1586	1772062114_F06	astrocytes_ependymal	4	
1592	1772058148_C05	astrocytes_ependymal	4	
1512	1772071014_D06	astrocytes_ependymal	4	
1565	1772062115_F11	astrocytes_ependymal	4	
1513	1772071014_F02	astrocytes_ependymal	4	
1505	1772062109_D11	astrocytes_ependymal	4	
1572	1772060240_B02	astrocytes_ependymal	4	
1528	1772063077_F03	astrocytes_ependymal	4	
1560	1772062128_F01	astrocytes_ependymal	4	
1511	1772071014_H07	astrocytes_ependymal	4	
1566	1772060240_F07	astrocytes_ependymal	4	
1609	1772062116_B01	astrocytes_ependymal	1	
1635	1772060240_H06	astrocytes_ependymal	1	
1510	1772071014_G04	astrocytes_ependymal	4	
1583	1772060240_H12	astrocytes_ependymal	4	
1621	1772067074_F02	astrocytes_ependymal	1	

1619	1772063061_D08	astrocytes_ependymal	4
1617	1772062111_A04	astrocytes_ependymal	4
1582	1772062109_E11	astrocytes_ependymal	1
1603	1772067073_H07	astrocytes_ependymal	4
1599	1772063077_E12	astrocytes_ependymal	4
1576	1772060240_B05	astrocytes_ependymal	4
1539	1772071015_G05	astrocytes_ependymal	1
1625	1772062109_G12	astrocytes_ependymal	4
1567	1772062113_F04	astrocytes_ependymal	1
1571	1772063077_F11	astrocytes_ependymal	4
1575	1772060240_D05	astrocytes_ependymal	4
1573	1772058148_C01	astrocytes_ependymal	4
1507	1772071014_C07	astrocytes_ependymal	4
1504	1772071014_H04	astrocytes_ependymal	4
1605	1772067073_E04	astrocytes_ependymal	4
1610	1772067063_A06	astrocytes_ependymal	4
1589	1772067063_G08	astrocytes_ependymal	4
1591	1772063064_E06	astrocytes_ependymal	4
1595	1772060224_E12	astrocytes_ependymal	4
1626	1772063078_G04	astrocytes_ependymal	4
1537	1772063064_D08	astrocytes_ependymal	4
1632	1772062128_E08	astrocytes_ependymal	4
1508	1772067063_E05	astrocytes_ependymal	4
1587	1772063062_C11	astrocytes_ependymal	4
1606	1772067064_C11	astrocytes_ependymal	4
1585	1772071041_B08	astrocytes_ependymal	4
1604	1772067066_C06	astrocytes_ependymal	4
1596	1772058148_E08	astrocytes_ependymal	4
1520	1772067069_C11	astrocytes_ependymal	4
1525	1772071014_A02	astrocytes_ependymal	4
1597	1772058177_C04	astrocytes_ependymal	4
1521	1772071014_E01	astrocytes_ependymal	4
1608	1772067063_E07	astrocytes_ependymal	4
1564	1772062109_A08	astrocytes_ependymal	4
1563	1772062128_C08	astrocytes_ependymal	2
1642	1772058148_D11	astrocytes_ependymal	2
1643	1772058148_C07	astrocytes_ependymal	2
1644	1772058148_B02	astrocytes_ependymal	4
1615	1772062128_G01	astrocytes_ependymal	4
1561	1772062118_A08	astrocytes_ependymal	4
1611	1772071040_B09	astrocytes_ependymal	4
1518	1772071015_H10	astrocytes_ependymal	4
1555	1772063065_E10	astrocytes_ependymal	4
1516	1772071015_D10	astrocytes_ependymal	4
1517	1772067064_A10	astrocytes_ependymal	4
1526	1772063071_H08	astrocytes_ependymal	4
1522	1772071015_D02	astrocytes_ependymal	4
1514	1772058171_H01	astrocytes_ependymal	4
1531	1772058177_D07	astrocytes_ependymal	2
1600	1772058148_C09	astrocytes_ependymal	4

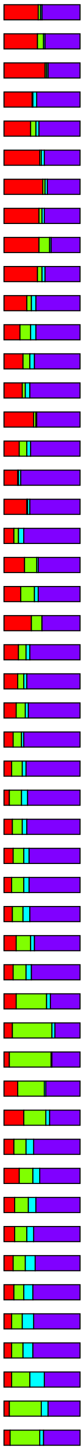

1535	1772058177_B03	astrocytes_ependymal	4	
1552	1772062118_G01	astrocytes_ependymal	4	
1577	1772060224_D09	astrocytes_ependymal	2	
1544	1772063068_F04	astrocytes_ependymal	4	
1574	1772060224_G08	astrocytes_ependymal	4	
1558	1772063071_H02	astrocytes_ependymal	4	
1536	1772058171_B03	astrocytes_ependymal	4	
1556	1772062115_D08	astrocytes_ependymal	4	
1538	1772071015_A12	astrocytes_ependymal	4	
1550	1772063071_H07	astrocytes_ependymal	4	
1601	1772063074_C07	astrocytes_ependymal	4	
1548	1772063071_B11	astrocytes_ependymal	4	
1532	1772058171_F07	astrocytes_ependymal	4	
1584	1772062118_G03	astrocytes_ependymal	4	
1578	1772062109_E04	astrocytes_ependymal	4	
1277	1772058148_E12	microglia	4	
1541	1772063061_F08	astrocytes_ependymal	4	
1678	1772063074_F11	endothelial-mural	3	
1684	1772058148_D02	endothelial-mural	3	
1523	1772062128_D05	astrocytes_ependymal	4	
1570	1772063077_G09	astrocytes_ependymal	4	
1557	1772060226_B09	astrocytes_ependymal	4	
1527	1772062128_E02	astrocytes_ependymal	3	
1622	1772060226_C10	astrocytes_ependymal	4	
1545	1772063065_D11	astrocytes_ependymal	4	
1338	1772062115_H11	microglia	3	
1529	1772058177_E09	astrocytes_ependymal	3	
1640	1772060226_E06	astrocytes_ependymal	3	
1274	1772062109_E05	microglia	4	

Table 3: Branch 1.2

node.label	cell.name	cell.group	main.topic	topics
486	1772060225_E04	pyramidal SS	1	
383	1772060225_E01	pyramidal SS	1	
78	1772062128_G08	interneurons	1	
508	1772063078_G09	pyramidal SS	1	
273	1772062128_E10	pyramidal SS	1	
80	1772062114_H03	interneurons	1	
121	1772063077_H05	interneurons	1	
326	1772062113_E01	pyramidal SS	1	
147	1772058148_H03	interneurons	1	
101	1772063061_B03	interneurons	1	
103	1772058177_G09	interneurons	1	
515	1772062128_F12	pyramidal SS	1	
116	1772062128_F10	interneurons	1	
434	1772058177_C10	pyramidal SS	1	
110	1772058148_F09	interneurons	1	
91	1772063064_G03	interneurons	1	
1645	1772058148_A12	astrocytes_ependymal	1	

1616	1772058148_F08	astrocytes_ependymal	1
393	1772063077_G04	pyramidal SS	1
501	1772063068_C05	pyramidal SS	1
391	1772063068_B10	pyramidal SS	1
371	1772071041_E04	pyramidal SS	1
523	1772063068_E03	pyramidal SS	1
1627	1772062128_E07	astrocytes_ependymal	1
1546	1772063064_H03	astrocytes_ependymal	1
534	1772063077_A07	pyramidal SS	1
429	1772063061_C02	pyramidal SS	1
447	1772062128_E06	pyramidal SS	1
558	1772062116_F11	pyramidal SS	1
521	1772063068_D02	pyramidal SS	1
246	1772067066_B03	pyramidal SS	1
410	1772062109_D03	pyramidal SS	1
222	1772071041_D04	pyramidal SS	1
455	1772062114_F10	pyramidal SS	1
331	1772063068_A06	pyramidal SS	1
291	1772062116_G05	pyramidal SS	1
84	1772062113_F08	interneurons	1
317	1772063068_G11	pyramidal SS	1
351	1772067064_A11	pyramidal SS	1
268	1772063063_E04	pyramidal SS	1
88	1772063078_F01	interneurons	1
423	1772063061_E06	pyramidal SS	1
1076	1772060224_C08	oligodendrocytes	1
479	1772062111_E07	pyramidal SS	1
1262	1772063061_B04	oligodendrocytes	1
81	1772062114_E02	interneurons	1
514	1772063078_C10	pyramidal SS	1
1116	1772062111_C03	oligodendrocytes	1
368	1772062111_H10	pyramidal SS	1
895	1772063061_D12	oligodendrocytes	1
415	1772062115_C09	pyramidal SS	1
618	1772063063_F10	oligodendrocytes	1
929	1772063078_F02	oligodendrocytes	1
396	1772062111_H11	pyramidal SS	1
1506	1772062111_B03	astrocytes_ependymal	1
102	1772063061_B05	interneurons	1
468	1772062111_H03	pyramidal SS	1
1081	1772063078_B08	oligodendrocytes	1
701	1772058148_B08	oligodendrocytes	1
513	1772060224_C05	pyramidal SS	1
969	1772063077_C01	oligodendrocytes	1
430	1772063061_C06	pyramidal SS	1
1054	1772060225_H10	oligodendrocytes	1
417	1772063061_H12	pyramidal SS	1
174	1772071040_B04	pyramidal SS	1
185	1772067064_C09	pyramidal SS	1
370	1772071040_A11	pyramidal SS	1

422	1772063061_D11	pyramidal SS	1
196	1772067069_B02	pyramidal SS	1
254	1772071041_A02	pyramidal SS	1
954	1772063061_F11	oligodendrocytes	1
218	1772071041_F09	pyramidal SS	1
605	1772067069_H05	oligodendrocytes	1
684	1772071041_D10	oligodendrocytes	1
483	1772071040_G12	pyramidal SS	1
261	1772067073_C04	pyramidal SS	1
436	1772063061_F06	pyramidal SS	1
865	1772063062_D05	oligodendrocytes	1
379	1772062111_B01	pyramidal SS	1
1061	1772062115_F05	oligodendrocytes	1
760	1772062113_F02	oligodendrocytes	1
1160	1772062109_D04	oligodendrocytes	1
419	1772058177_G03	pyramidal SS	1
994	1772060225_H12	oligodendrocytes	1
1107	1772063062_F09	oligodendrocytes	1
83	1772062115_A12	interneurons	1
791	1772062113_C03	oligodendrocytes	1
919	1772062115_C04	oligodendrocytes	1
792	1772062116_B12	oligodendrocytes	1
710	1772063062_B03	oligodendrocytes	1
711	1772071040_A06	oligodendrocytes	1
1194	1772063061_E12	oligodendrocytes	1
676	1772071040_C12	oligodendrocytes	1
931	1772062115_A02	oligodendrocytes	1
1145	1772062115_G11	oligodendrocytes	1
652	1772063077_B07	oligodendrocytes	1
603	1772058171_H07	oligodendrocytes	1
925	1772063078_C09	oligodendrocytes	1
99	1772063063_F01	interneurons	1
930	1772063078_B11	oligodendrocytes	1
613	1772063061_A11	oligodendrocytes	1
878	1772063063_C10	oligodendrocytes	1
564	1772067064_G07	oligodendrocytes	1
963	1772060225_F09	oligodendrocytes	1
833	1772063063_A01	oligodendrocytes	1
611	1772063063_F03	oligodendrocytes	1
815	1772063063_D06	oligodendrocytes	1
86	1772063064_G08	interneurons	1
1080	1772063078_D05	oligodendrocytes	1
896	1772063078_B01	oligodendrocytes	1
824	1772062113_F03	oligodendrocytes	1
843	1772060240_C04	oligodendrocytes	1
1097	1772062111_G04	oligodendrocytes	1
1039	1772062111_B05	oligodendrocytes	1
1092	1772062109_C11	oligodendrocytes	1
610	1772060240_C09	oligodendrocytes	1
1196	1772062113_H11	oligodendrocytes	1

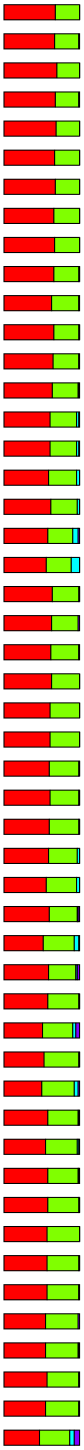

1114	1772062111_E08	oligodendrocytes	1
980	1772060224_H11	oligodendrocytes	1
937	1772060225_G06	oligodendrocytes	1
728	1772063063_H07	oligodendrocytes	2
690	1772063070_G01	oligodendrocytes	1
820	1772058148_A04	oligodendrocytes	2
888	1772071041_A10	oligodendrocytes	1
956	1772063078_G08	oligodendrocytes	1
1067	1772060224_F03	oligodendrocytes	1
1009	1772060226_G06	oligodendrocytes	2
1109	1772060226_H10	oligodendrocytes	1
803	1772062113_B03	oligodendrocytes	2
1087	1772063068_B05	oligodendrocytes	2
1106	1772063065_A02	oligodendrocytes	2
821	1772062113_A12	oligodendrocytes	2
1088	1772063077_D10	oligodendrocytes	2
1091	1772063065_B03	oligodendrocytes	2
1042	1772062111_C02	oligodendrocytes	2
898	1772062115_A04	oligodendrocytes	2
1167	1772062115_B11	oligodendrocytes	2
1074	1772063068_E05	oligodendrocytes	2
672	1772063070_H07	oligodendrocytes	2
1038	1772063068_F10	oligodendrocytes	2
1120	1772063063_E02	oligodendrocytes	2
782	1772063061_A07	oligodendrocytes	2
641	1772063077_C03	oligodendrocytes	2
982	1772063064_D09	oligodendrocytes	2
1066	1772063068_A09	oligodendrocytes	2
730	1772062111_B02	oligodendrocytes	2
1193	1772062113_G06	oligodendrocytes	2
1133	1772062128_G02	oligodendrocytes	2
906	1772063078_C07	oligodendrocytes	2
1043	1772060225_B06	oligodendrocytes	2
1099	1772067070_C09	oligodendrocytes	2
827	1772063062_G01	oligodendrocytes	2
1098	1772063064_G09	oligodendrocytes	2
920	1772062111_B04	oligodendrocytes	2
712	1772058148_F02	oligodendrocytes	2
1629	1772063078_G06	astrocytes_ependymal	2
1118	1772062118_C07	oligodendrocytes	2
1002	1772060226_D03	oligodendrocytes	2
1084	1772063062_D10	oligodendrocytes	2
948	1772063061_C03	oligodendrocytes	2
1131	1772062128_E09	oligodendrocytes	2
1028	1772060226_B02	oligodendrocytes	2
645	1772062116_H06	oligodendrocytes	2
802	1772062116_E07	oligodendrocytes	2
1073	1772063062_F08	oligodendrocytes	2
1058	1772062109_F08	oligodendrocytes	2
1056	1772060224_A01	oligodendrocytes	2

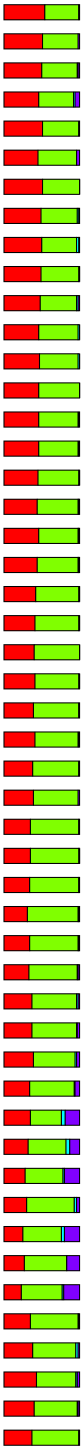

1195	1772062113_E02	oligodendrocytes	2
858	1772063074_G07	oligodendrocytes	2
1108	1772063062_F01	oligodendrocytes	2
988	1772063063_H08	oligodendrocytes	2
1144	1772067064_A09	oligodendrocytes	2
1093	1772063078_D09	oligodendrocytes	2
905	1772063068_G06	oligodendrocytes	2
1095	1772063062_D08	oligodendrocytes	2
829	1772063062_C01	oligodendrocytes	2
575	1772062111_F07	oligodendrocytes	2
576	1772067070_A05	oligodendrocytes	2
1192	1772063070_D05	oligodendrocytes	2
1094	1772063065_H08	oligodendrocytes	2
655	1772062113_D01	oligodendrocytes	2
766	1772062114_F03	oligodendrocytes	2
921	1772060225_C07	oligodendrocytes	2
1077	1772063061_D05	oligodendrocytes	2
816	1772062116_D04	oligodendrocytes	2
867	1772060240_F02	oligodendrocytes	2
1173	1772062113_C02	oligodendrocytes	2
819	1772062113_A02	oligodendrocytes	2
866	1772063061_D01	oligodendrocytes	2
609	1772067069_F06	oligodendrocytes	2
565	1772067070_D08	oligodendrocytes	2
614	1772063063_C02	oligodendrocytes	2
1117	1772062111_D09	oligodendrocytes	2
911	1772063077_E09	oligodendrocytes	2
1016	1772060226_G05	oligodendrocytes	2
570	1772063063_C07	oligodendrocytes	2
989	1772060225_G07	oligodendrocytes	2
1035	1772060226_B12	oligodendrocytes	2
1179	1772062111_B07	oligodendrocytes	2
908	1772062113_A08	oligodendrocytes	2
607	1772067069_B03	oligodendrocytes	2
854	1772060240_F04	oligodendrocytes	2
619	1772060225_E07	oligodendrocytes	2
699	1772063070_C06	oligodendrocytes	2
608	1772067074_E07	oligodendrocytes	2
761	1772062113_B01	oligodendrocytes	2
1070	1772060225_D09	oligodendrocytes	2
637	1772062109_A03	oligodendrocytes	2
774	1772062113_D09	oligodendrocytes	2
1171	1772062113_D12	oligodendrocytes	2
668	1772071041_G10	oligodendrocytes	2
1165	1772062118_G11	oligodendrocytes	2
901	1772060225_C05	oligodendrocytes	2
801	1772062116_E08	oligodendrocytes	2
951	1772062114_B12	oligodendrocytes	2
936	1772062116_E01	oligodendrocytes	2
965	1772063079_A12	oligodendrocytes	2

973	1772062114_H04	oligodendrocytes	2
1143	1772062128_D09	oligodendrocytes	2
926	1772060240_F10	oligodendrocytes	2
785	1772058171_D10	oligodendrocytes	2
938	1772060240_A12	oligodendrocytes	2
1178	1772062109_H02	oligodendrocytes	2
571	1772062114_G10	oligodendrocytes	2
950	1772062115_A06	oligodendrocytes	2
606	1772063063_E07	oligodendrocytes	2
656	1772062109_E08	oligodendrocytes	2
975	1772063064_E03	oligodendrocytes	2
1159	1772062109_B05	oligodendrocytes	2
1079	1772063077_A12	oligodendrocytes	2
811	1772062109_A12	oligodendrocytes	2
859	1772063063_G02	oligodendrocytes	2
1129	1772058148_E07	oligodendrocytes	2
773	1772062113_B04	oligodendrocytes	2
579	1772060226_F05	oligodendrocytes	2
877	1772063078_H04	oligodendrocytes	2
1157	1772062111_C04	oligodendrocytes	2
823	1772063063_B06	oligodendrocytes	2
588	1772062115_C01	oligodendrocytes	2
893	1772062118_B03	oligodendrocytes	2
1082	1772063071_A05	oligodendrocytes	2
884	1772063063_B11	oligodendrocytes	2
581	1772062109_A05	oligodendrocytes	2
847	1772063062_B04	oligodendrocytes	2
781	1772062109_A07	oligodendrocytes	2
879	1772062113_G09	oligodendrocytes	2
913	1772063074_G04	oligodendrocytes	2
648	1772062114_B01	oligodendrocytes	2
612	1772063063_B04	oligodendrocytes	2
834	1772063063_A12	oligodendrocytes	2
585	1772063063_F08	oligodendrocytes	2
654	1772062113_B09	oligodendrocytes	2
740	1772060240_F06	oligodendrocytes	2
752	1772060240_B12	oligodendrocytes	2
825	1772062109_C03	oligodendrocytes	2
1075	1772062114_C06	oligodendrocytes	2
686	1772067069_D04	oligodendrocytes	2
1057	1772060225_C02	oligodendrocytes	2
1021	1772062114_A06	oligodendrocytes	2
714	1772067074_D12	oligodendrocytes	2
732	1772063063_D08	oligodendrocytes	2
734	1772063063_G10	oligodendrocytes	2
863	1772062111_E04	oligodendrocytes	2
891	1772071041_B04	oligodendrocytes	2
615	1772060240_F11	oligodendrocytes	2
627	1772060225_H11	oligodendrocytes	2
1053	1772063077_F04	oligodendrocytes	2

837	1772067069_G04	oligodendrocytes	2
736	1772062118_C04	oligodendrocytes	2
681	1772067064_H01	oligodendrocytes	2
738	1772062109_H04	oligodendrocytes	2
674	1772067066_B04	oligodendrocytes	2
598	1772063061_A09	oligodendrocytes	2
904	1772062118_F10	oligodendrocytes	2
907	1772063078_B07	oligodendrocytes	2
694	1772067064_D10	oligodendrocytes	2
636	1772062111_C08	oligodendrocytes	2
1047	1772060225_A07	oligodendrocytes	2
835	1772071014_D12	oligodendrocytes	2
703	1772067073_C02	oligodendrocytes	2
693	1772067066_E12	oligodendrocytes	2
943	1772063061_C05	oligodendrocytes	2
731	1772067074_F04	oligodendrocytes	2
735	1772071040_D04	oligodendrocytes	2
1187	1772063061_F12	oligodendrocytes	2
1180	1772062111_B10	oligodendrocytes	2
876	1772062128_A01	oligodendrocytes	2
729	1772067074_A11	oligodendrocytes	2
1174	1772062114_A01	oligodendrocytes	2
754	1772062114_H12	oligodendrocytes	2
836	1772062109_A09	oligodendrocytes	2
1245	1772063064_F07	oligodendrocytes	2
616	1772063063_F05	oligodendrocytes	2
942	1772062114_D06	oligodendrocytes	2
723	1772062111_B11	oligodendrocytes	2
1184	1772063070_C10	oligodendrocytes	2
1185	1772062111_A07	oligodendrocytes	2
638	1772062111_C01	oligodendrocytes	2
1559	1772063077_A11	astrocytes_ependymal	2
922	1772062114_B09	oligodendrocytes	2
1208	1772063064_E07	oligodendrocytes	2
644	1772062128_B11	oligodendrocytes	2
1113	1772067074_A01	oligodendrocytes	2
1568	1772062118_A10	astrocytes_ependymal	2
1025	1772062115_E11	oligodendrocytes	2
1137	1772062128_F09	oligodendrocytes	2
983	1772062116_F10	oligodendrocytes	2
624	1772060226_F10	oligodendrocytes	2
678	1772058148_G09	oligodendrocytes	2
883	1772063062_E01	oligodendrocytes	2
1065	1772062113_E10	oligodendrocytes	2
849	1772063077_D02	oligodendrocytes	2
620	1772060225_D10	oligodendrocytes	2
717	1772067064_B06	oligodendrocytes	2
992	1772063064_H11	oligodendrocytes	2
1063	1772062115_B03	oligodendrocytes	2
874	1772062118_C01	oligodendrocytes	2

1060	1772060225_D08	oligodendrocytes	2
848	1772062114_E05	oligodendrocytes	2
623	1772060226_G12	oligodendrocytes	2
746	1772063061_B12	oligodendrocytes	2
1128	1772058148_G01	oligodendrocytes	2
726	1772060240_H04	oligodendrocytes	2
953	1772063061_B11	oligodendrocytes	2
1040	1772062111_C06	oligodendrocytes	2
583	1772060240_A10	oligodendrocytes	2
934	1772063078_F11	oligodendrocytes	2
1049	1772062118_G06	oligodendrocytes	2
1146	1772063063_A11	oligodendrocytes	2
765	1772062113_F07	oligodendrocytes	2
1045	1772058148_B06	oligodendrocytes	2
642	1772062116_A12	oligodendrocytes	2
993	1772062114_D10	oligodendrocytes	2
660	1772062116_F01	oligodendrocytes	2
807	1772058148_A06	oligodendrocytes	2
1048	1772063074_A12	oligodendrocytes	2
981	1772063074_H10	oligodendrocytes	2
597	1772058177_E11	oligodendrocytes	2
961	1772060224_A02	oligodendrocytes	2
928	1772063078_C08	oligodendrocytes	2
685	1772058148_F07	oligodendrocytes	2
621	1772060225_B07	oligodendrocytes	2
817	1772063064_D06	oligodendrocytes	2
574	1772067063_D03	oligodendrocytes	2
1020	1772060226_B10	oligodendrocytes	2
702	1772058148_D07	oligodendrocytes	2
1096	1772063074_E01	oligodendrocytes	2
1183	1772062113_C11	oligodendrocytes	2
688	1772058148_G08	oligodendrocytes	2
861	1772063061_D10	oligodendrocytes	2
1068	1772062128_G11	oligodendrocytes	2
957	1772063074_A09	oligodendrocytes	2
639	1772062111_E06	oligodendrocytes	2
894	1772063061_G11	oligodendrocytes	2
705	1772062116_C06	oligodendrocytes	2
640	1772062109_H10	oligodendrocytes	2
1201	1772063061_D02	oligodendrocytes	2
716	1772067074_E11	oligodendrocytes	2
790	1772062111_D05	oligodendrocytes	2
663	1772062118_G05	oligodendrocytes	2
762	1772062111_C09	oligodendrocytes	2
753	1772062113_B06	oligodendrocytes	2
572	1772063061_G05	oligodendrocytes	2
1124	1772063063_C08	oligodendrocytes	2
667	1772071040_D02	oligodendrocytes	2
691	1772067070_H09	oligodendrocytes	2
1202	1772063078_F04	oligodendrocytes	2

1036	1772060225_E12	oligodendrocytes	2	
748	1772062116_C11	oligodendrocytes	2	
721	1772071040_H06	oligodendrocytes	2	
1052	1772062128_C05	oligodendrocytes	2	
1037	1772062115_E07	oligodendrocytes	2	
999	1772060225_D07	oligodendrocytes	2	
741	1772071040_C11	oligodendrocytes	2	
1041	1772063065_H06	oligodendrocytes	2	
873	1772063062_E11	oligodendrocytes	2	
1182	1772062113_C06	oligodendrocytes	2	
886	1772060225_E02	oligodendrocytes	2	
682	1772067073_D10	oligodendrocytes	2	
1071	1772062113_C09	oligodendrocytes	2	
653	1772062114_B10	oligodendrocytes	2	
850	1772060240_G11	oligodendrocytes	2	
695	1772067063_B07	oligodendrocytes	2	
689	1772067064_B10	oligodendrocytes	2	
697	1772067074_F05	oligodendrocytes	2	
733	1772071040_G11	oligodendrocytes	2	
742	1772067064_C10	oligodendrocytes	2	
719	1772067069_A09	oligodendrocytes	2	
725	1772067069_A02	oligodendrocytes	2	
1207	1772062115_C07	oligodendrocytes	2	
1168	1772062115_G12	oligodendrocytes	2	
1155	1772062115_E03	oligodendrocytes	2	
1051	1772062115_C10	oligodendrocytes	2	
626	1772063079_C10	oligodendrocytes	2	
1216	1772058171_A02	oligodendrocytes	2	
955	1772062114_A08	oligodendrocytes	2	
763	1772062111_G02	oligodendrocytes	2	
949	1772062114_H10	oligodendrocytes	2	
968	1772067073_A08	oligodendrocytes	2	
709	1772067073_E11	oligodendrocytes	2	
1229	1772058171_D01	oligodendrocytes	2	
1059	1772062115_G04	oligodendrocytes	2	
715	1772062116_F05	oligodendrocytes	2	
1258	1772058171_C04	oligodendrocytes	2	
1190	1772062115_E04	oligodendrocytes	2	
814	1772058171_F06	oligodendrocytes	2	
959	1772063079_A06	oligodendrocytes	2	
910	1772060225_C11	oligodendrocytes	2	
772	1772058171_G08	oligodendrocytes	2	
1203	1772062113_F12	oligodendrocytes	2	
780	1772058171_C03	oligodendrocytes	2	
784	1772058171_C12	oligodendrocytes	2	
768	1772058171_F05	oligodendrocytes	2	
903	1772063078_B06	oligodendrocytes	2	
812	1772062115_B06	oligodendrocytes	2	
1062	1772060224_B08	oligodendrocytes	2	
875	1772071040_A08	oligodendrocytes	2	

677	1772071040_C10	oligodendrocytes	2
1121	1772058171_E06	oligodendrocytes	2
909	1772062115_C06	oligodendrocytes	2
664	1772071041_E05	oligodendrocytes	2
670	1772071041_F05	oligodendrocytes	2
880	1772062115_E01	oligodendrocytes	2
698	1772071040_C04	oligodendrocytes	2
635	1772062113_G05	oligodendrocytes	2
897	1772062113_B11	oligodendrocytes	2
844	1772067074_F06	oligodendrocytes	2
778	1772062114_A03	oligodendrocytes	2
1223	1772063074_D10	oligodendrocytes	2
669	1772067066_C04	oligodendrocytes	2
739	1772071040_G05	oligodendrocytes	2
915	1772060225_G04	oligodendrocytes	2
679	1772067066_H05	oligodendrocytes	2
680	1772067064_G04	oligodendrocytes	2
1198	1772062113_D04	oligodendrocytes	2
666	1772067065_G08	oligodendrocytes	2
868	1772062114_F05	oligodendrocytes	2
1175	1772062118_D05	oligodendrocytes	2
750	1772060225_D12	oligodendrocytes	2
683	1772067073_F06	oligodendrocytes	2
813	1772071040_D12	oligodendrocytes	2
970	1772060225_A12	oligodendrocytes	2
846	1772071041_A08	oligodendrocytes	2
789	1772062128_H11	oligodendrocytes	2
1050	1772062114_F08	oligodendrocytes	2
589	1772060225_A11	oligodendrocytes	2
1132	1772067070_C12	oligodendrocytes	2
722	1772071040_H10	oligodendrocytes	2
869	1772071041_A12	oligodendrocytes	2
862	1772062114_E06	oligodendrocytes	2
964	1772063074_G10	oligodendrocytes	2
1177	1772062115_B08	oligodendrocytes	2
718	1772067073_D01	oligodendrocytes	2
1163	1772062118_C10	oligodendrocytes	2
743	1772063070_G06	oligodendrocytes	2
1003	1772060226_D04	oligodendrocytes	2
584	1772062115_D07	oligodendrocytes	2
1005	1772060226_B11	oligodendrocytes	2
651	1772062118_E08	oligodendrocytes	2
1176	1772062118_G02	oligodendrocytes	2
590	1772063078_B09	oligodendrocytes	2
996	1772060226_A11	oligodendrocytes	2
917	1772060224_B03	oligodendrocytes	2
902	1772062115_D04	oligodendrocytes	2
995	1772060226_A01	oligodendrocytes	2
818	1772063074_G12	oligodendrocytes	2
1162	1772063074_A01	oligodendrocytes	2

871	1772063062_G05	oligodendrocytes	2	
857	1772071040_D05	oligodendrocytes	2	
977	1772060224_E09	oligodendrocytes	2	
783	1772062111_G01	oligodendrocytes	2	
708	1772062118_D02	oligodendrocytes	2	
853	1772060224_H04	oligodendrocytes	2	
914	1772062115_B10	oligodendrocytes	2	
851	1772063074_C12	oligodendrocytes	2	
1055	1772063074_H05	oligodendrocytes	2	
839	1772063074_A11	oligodendrocytes	2	
1104	1772063068_H06	oligodendrocytes	2	
881	1772063074_F10	oligodendrocytes	2	
749	1772063061_F04	oligodendrocytes	2	
594	1772060225_A05	oligodendrocytes	2	
658	1772062113_G12	oligodendrocytes	2	
755	1772063062_A06	oligodendrocytes	2	
578	1772062114_A12	oligodendrocytes	2	
1015	1772060226_B03	oligodendrocytes	2	
1072	1772062128_B04	oligodendrocytes	2	
657	1772062113_D08	oligodendrocytes	2	
661	1772062115_C03	oligodendrocytes	2	
617	1772060225_F06	oligodendrocytes	2	
1069	1772060225_C04	oligodendrocytes	2	
604	1772067073_B03	oligodendrocytes	2	
602	1772058177_E10	oligodendrocytes	2	
633	1772062111_F06	oligodendrocytes	2	
1110	1772062118_H05	oligodendrocytes	2	
1210	1772062113_E07	oligodendrocytes	2	
631	1772062128_F06	oligodendrocytes	2	
805	1772062114_E11	oligodendrocytes	2	
900	1772063078_E11	oligodendrocytes	2	
1123	1772066104_C11	oligodendrocytes	2	
1215	1772058177_C01	oligodendrocytes	2	
1230	1772058177_F08	oligodendrocytes	2	
990	1772060226_D12	oligodendrocytes	2	
1158	1772062115_H05	oligodendrocytes	2	
1212	1772058177_E07	oligodendrocytes	2	
1222	1772058171_A04	oligodendrocytes	2	
1214	1772058171_E07	oligodendrocytes	2	
1228	1772058171_D04	oligodendrocytes	2	
771	1772060224_C04	oligodendrocytes	2	
759	1772062113_G01	oligodendrocytes	2	
757	1772063079_C12	oligodendrocytes	2	
600	1772058171_F09	oligodendrocytes	2	
601	1772058171_B01	oligodendrocytes	2	
840	1772063062_G11	oligodendrocytes	2	
767	1772062111_D11	oligodendrocytes	2	
997	1772060226_D01	oligodendrocytes	2	
1001	1772060226_A07	oligodendrocytes	2	
650	1772063074_C02	oligodendrocytes	2	

1032	1772060226_D06	oligodendrocytes	2
625	1772062109_B08	oligodendrocytes	2
1004	1772060226_E10	oligodendrocytes	2
595	1772062115_F08	oligodendrocytes	2
587	1772060224_B04	oligodendrocytes	2
967	1772063074_F12	oligodendrocytes	2
828	1772062116_E02	oligodendrocytes	2
751	1772058177_C06	oligodendrocytes	2
872	1772063062_E08	oligodendrocytes	2
580	1772062115_A09	oligodendrocytes	2
860	1772063061_G08	oligodendrocytes	2
1024	1772060226_B07	oligodendrocytes	2
1254	1772058177_B09	oligodendrocytes	2
1170	1772063062_H01	oligodendrocytes	2
889	1772062118_A03	oligodendrocytes	2
707	1772062116_G03	oligodendrocytes	2
1008	1772060226_H11	oligodendrocytes	2
1148	1772058148_B01	oligodendrocytes	2
985	1772060224_B05	oligodendrocytes	2
1010	1772060226_H04	oligodendrocytes	2
659	1772062115_H08	oligodendrocytes	2
764	1772060224_A07	oligodendrocytes	2
1150	1772067063_G11	oligodendrocytes	2
1151	1772062128_F08	oligodendrocytes	2
569	1772058177_D09	oligodendrocytes	2
1085	1772063065_D03	oligodendrocytes	2
1225	1772058177_E06	oligodendrocytes	2
1189	1772062118_E01	oligodendrocytes	2
870	1772062116_H05	oligodendrocytes	2
1014	1772060226_C07	oligodendrocytes	2
1029	1772060226_E05	oligodendrocytes	2
706	1772062116_A02	oligodendrocytes	2
795	1772062118_F08	oligodendrocytes	2
798	1772063062_C10	oligodendrocytes	2
1206	1772058171_B09	oligodendrocytes	2
986	1772060224_D01	oligodendrocytes	2
1102	1772062115_A10	oligodendrocytes	2
1139	1772062118_A05	oligodendrocytes	2
1243	1772058171_B07	oligodendrocytes	2
744	1772062128_H02	oligodendrocytes	2
1211	1772058171_B04	oligodendrocytes	2
890	1772063079_F04	oligodendrocytes	2
1219	1772058171_D03	oligodendrocytes	2
1248	1772058177_E12	oligodendrocytes	2
1241	1772058177_B06	oligodendrocytes	2
1261	1772062128_C10	oligodendrocytes	2
1186	1772062111_H04	oligodendrocytes	2
1218	1772062111_E05	oligodendrocytes	2
1122	1772062128_H08	oligodendrocytes	2
628	1772062111_F10	oligodendrocytes	2

960	1772063077_D09	oligodendrocytes	2
593	1772063078_A11	oligodendrocytes	2
1000	1772060226_F06	oligodendrocytes	2
573	1772058171_C02	oligodendrocytes	2
947	1772062114_H05	oligodendrocytes	2
622	1772063063_E10	oligodendrocytes	2
864	1772060224_H05	oligodendrocytes	2
916	1772060224_A11	oligodendrocytes	2
1205	1772062116_H02	oligodendrocytes	2
1161	1772060224_D10	oligodendrocytes	2
885	1772063062_A08	oligodendrocytes	2
1250	1772058177_B04	oligodendrocytes	2
1237	1772058177_F06	oligodendrocytes	2
1224	1772058171_G01	oligodendrocytes	2
599	1772058177_A06	oligodendrocytes	2
1217	1772058171_B05	oligodendrocytes	2
1247	1772058171_B06	oligodendrocytes	2
1234	1772058171_G11	oligodendrocytes	2
856	1772062115_D05	oligodendrocytes	2
1249	1772058171_D02	oligodendrocytes	2
1235	1772058171_A09	oligodendrocytes	2
1246	1772058171_E10	oligodendrocytes	2
1252	1772058171_D08	oligodendrocytes	2
788	1772058171_H02	oligodendrocytes	2
1209	1772063079_D11	oligodendrocytes	2
1260	1772058171_C10	oligodendrocytes	2
1253	1772058171_E05	oligodendrocytes	2
1257	1772058171_G10	oligodendrocytes	2
1251	1772062128_F04	oligodendrocytes	2
971	1772063079_F09	oligodendrocytes	2
1259	1772058171_C08	oligodendrocytes	2
1086	1772063079_G07	oligodendrocytes	2
962	1772062118_B05	oligodendrocytes	2
1119	1772063079_H01	oligodendrocytes	2
1232	1772058171_F03	oligodendrocytes	2
831	1772063079_D07	oligodendrocytes	2
1239	1772058171_G03	oligodendrocytes	2
720	1772071041_D06	oligodendrocytes	2
830	1772063074_H11	oligodendrocytes	2
974	1772062115_D03	oligodendrocytes	2
799	1772058171_C09	oligodendrocytes	2
696	1772062118_D03	oligodendrocytes	2
978	1772063079_H03	oligodendrocytes	2
1101	1772062109_B11	oligodendrocytes	2
1153	1772063074_B06	oligodendrocytes	2
1111	1772062118_B06	oligodendrocytes	2
1012	1772060226_F04	oligodendrocytes	2
713	1772058148_A10	oligodendrocytes	2
1006	1772060226_A08	oligodendrocytes	2
1166	1772062111_C05	oligodendrocytes	2

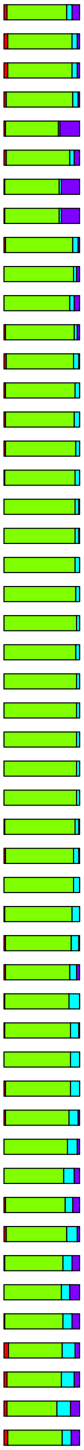

756	1772062109_G05	oligodendrocytes	2
1083	1772063078_A09	oligodendrocytes	2
838	1772062111_E11	oligodendrocytes	2
665	1772063061_H11	oligodendrocytes	2
1136	1772062128_E12	oligodendrocytes	2
800	1772063062_C07	oligodendrocytes	2
1244	1772058177_G05	oligodendrocytes	2
1033	1772060226_A10	oligodendrocytes	2
1233	1772058171_A11	oligodendrocytes	2
1125	1772062118_H03	oligodendrocytes	2
727	1772062116_B06	oligodendrocytes	2
1017	1772060226_A12	oligodendrocytes	2
1026	1772060226_G10	oligodendrocytes	2
1064	1772063074_C03	oligodendrocytes	2
940	1772060224_A06	oligodendrocytes	2
1220	1772058177_A03	oligodendrocytes	2
945	1772063061_E08	oligodendrocytes	2
1126	1772063074_E07	oligodendrocytes	2
1226	1772058177_C02	oligodendrocytes	2
804	1772062116_A09	oligodendrocytes	2
1636	1772063078_G03	astrocytes_ependymal	2
692	1772058148_G11	oligodendrocytes	2
1027	1772060226_A06	oligodendrocytes	2
1149	1772058148_A11	oligodendrocytes	2
826	1772062116_F04	oligodendrocytes	2
952	1772063079_E08	oligodendrocytes	2
806	1772062128_H05	oligodendrocytes	2
1240	1772058177_A01	oligodendrocytes	2
1169	1772062118_F01	oligodendrocytes	2
646	1772062113_D10	oligodendrocytes	2
976	1772063079_D08	oligodendrocytes	2
855	1772063062_F12	oligodendrocytes	2
912	1772063074_A04	oligodendrocytes	2
662	1772062115_G07	oligodendrocytes	2
1112	1772062116_F08	oligodendrocytes	2
704	1772067073_G10	oligodendrocytes	2
1238	1772058171_B08	oligodendrocytes	2
918	1772060224_C10	oligodendrocytes	2
1172	1772062111_E12	oligodendrocytes	2
832	1772071040_G04	oligodendrocytes	2
632	1772062111_F11	oligodendrocytes	2
966	1772063079_D01	oligodendrocytes	2
724	1772067064_C08	oligodendrocytes	2
1255	1772058177_C12	oligodendrocytes	2
1044	1772062128_E11	oligodendrocytes	2
1221	1772058171_E02	oligodendrocytes	2
673	1772071041_F06	oligodendrocytes	2
1018	1772063065_F12	oligodendrocytes	2
779	1772058177_A02	oligodendrocytes	2
629	1772062111_F05	oligodendrocytes	2

1231	1772058171_C01	oligodendrocytes	2
939	1772063079_C01	oligodendrocytes	2
797	1772058171_D06	oligodendrocytes	2
892	1772062116_A11	oligodendrocytes	2
887	1772063061_F05	oligodendrocytes	2
1227	1772058177_B05	oligodendrocytes	2
1115	1772063078_D12	oligodendrocytes	2
1256	1772058177_D05	oligodendrocytes	2
769	1772062111_E03	oligodendrocytes	2
1164	1772063062_E06	oligodendrocytes	2
808	1772062111_G11	oligodendrocytes	2
899	1772060225_G01	oligodendrocytes	2
1188	1772063079_H02	oligodendrocytes	2
1236	1772058171_F08	oligodendrocytes	2
972	1772063078_A03	oligodendrocytes	2
941	1772063061_H10	oligodendrocytes	2
1135	1772058177_F03	oligodendrocytes	2
1204	1772063079_C04	oligodendrocytes	3
1141	1772062128_C01	oligodendrocytes	2
1100	1772060225_D06	oligodendrocytes	2
1242	1772058148_G12	oligodendrocytes	2
630	1772058177_D02	oligodendrocytes	2
1034	1772060226_E11	oligodendrocytes	2
1138	1772058177_D08	oligodendrocytes	2
1156	1772062118_H12	oligodendrocytes	2
1046	1772063079_E02	oligodendrocytes	2
1105	1772062111_A10	oligodendrocytes	2
1007	1772060226_G04	oligodendrocytes	2
1281	1772062111_G10	microglia	3
932	1772060224_D03	oligodendrocytes	2
1181	1772062109_F06	oligodendrocytes	2
810	1772060224_G06	oligodendrocytes	2
747	1772058148_C02	oligodendrocytes	2
1213	1772058177_D12	oligodendrocytes	2
987	1772063079_D09	oligodendrocytes	2
1322	1772062118_H01	microglia	2
1562	1772062114_B04	astrocytes_ependymal	2
1011	1772060226_G11	oligodendrocytes	2
777	1772062113_G11	oligodendrocytes	2
745	1772062128_D03	oligodendrocytes	2
758	1772062116_G06	oligodendrocytes	2
566	1772058177_D03	oligodendrocytes	2
1019	1772060226_B04	oligodendrocytes	2
591	1772063062_E07	oligodendrocytes	2
991	1772063062_D01	oligodendrocytes	2
933	1772062111_A12	oligodendrocytes	2
882	1772063068_D10	oligodendrocytes	2
775	1772062109_C07	oligodendrocytes	2
786	1772062109_B01	oligodendrocytes	2
687	1772058148_A01	oligodendrocytes	2

582	1772062116_B05	oligodendrocytes	2
776	1772062109_F01	oligodendrocytes	2
596	1772062128_B02	oligodendrocytes	2
1200	1772063079_E09	oligodendrocytes	2
568	1772063070_G03	oligodendrocytes	2
592	1772062115_G09	oligodendrocytes	2
1022	1772060226_C04	oligodendrocytes	2
634	1772062109_C06	oligodendrocytes	2
842	1772062109_F04	oligodendrocytes	2
1031	1772060226_E12	oligodendrocytes	2
998	1772060226_D11	oligodendrocytes	2
944	1772060225_F12	oligodendrocytes	2
1154	1772058177_E04	oligodendrocytes	2
841	1772062118_H04	oligodendrocytes	2
700	1772058148_A03	oligodendrocytes	2
852	1772062118_H10	oligodendrocytes	2
946	1772062118_E02	oligodendrocytes	2
935	1772060240_G10	oligodendrocytes	2
958	1772060225_B12	oligodendrocytes	2
586	1772060224_G07	oligodendrocytes	2
979	1772062111_H07	oligodendrocytes	2
845	1772063063_B10	oligodendrocytes	2
1127	1772063063_A04	oligodendrocytes	2
1078	1772062109_D10	oligodendrocytes	2
1191	1772062109_F12	oligodendrocytes	2
1152	1772058148_A09	oligodendrocytes	2
577	1772062111_H05	oligodendrocytes	2
1197	1772062111_H02	oligodendrocytes	2
643	1772062109_E07	oligodendrocytes	2
822	1772063062_H08	oligodendrocytes	2
647	1772062109_G03	oligodendrocytes	2
794	1772062114_C07	oligodendrocytes	2
1130	1772063061_E07	oligodendrocytes	2
671	1772063070_E07	oligodendrocytes	2
1023	1772060226_H01	oligodendrocytes	2
1103	1772062118_D10	oligodendrocytes	2
737	1772062109_C12	oligodendrocytes	2
1199	1772063079_D02	oligodendrocytes	2
675	1772067074_F09	oligodendrocytes	2
567	1772067074_C07	oligodendrocytes	2
770	1772062113_A05	oligodendrocytes	2
1142	1772063063_E05	oligodendrocytes	2
796	1772062114_B03	oligodendrocytes	2
984	1772063078_E03	oligodendrocytes	2
1030	1772060226_C03	oligodendrocytes	2
1302	1772062128_D02	microglia	1
1013	1772060226_F08	oligodendrocytes	2
1140	1772063063_E06	oligodendrocytes	2
793	1772062118_F02	oligodendrocytes	2
1134	1772058177_D06	oligodendrocytes	3

809	1772062113_A01	oligodendrocytes	2	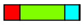
924	1772063078_F05	oligodendrocytes	1	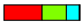
787	1772063079_F07	oligodendrocytes	2	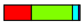
923	1772063062_F03	oligodendrocytes	1	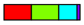
1090	1772063078_G01	oligodendrocytes	1	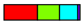
927	1772062116_E04	oligodendrocytes	2	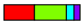
649	1772062113_H02	oligodendrocytes	2	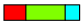
